# Genome-wide Comparative Analysis of Annexin Superfamily in Plants

**DOI:** 10.1371/journal.pone.0047801

**Published:** 2012-11-02

**Authors:** Sravan Kumar Jami, Greg B. Clark, Belay T. Ayele, Paula Ashe, Pulugurtha Bharadwaja Kirti

**Affiliations:** 1 Department of Plant Science, University of Manitoba, Winnipeg, Manitoba, Canada; 2 Section of Molecular Cell and Developmental Biology, University of Texas, Austin, Texas, United States of America; 3 National Research Council Canada, Saskatoon, Saskatchewan, Canada; 4 Department of Plant Sciences, School of Life Sciences, University of Hyderabad, Hyderabad, Andhra Pradesh, India; Children’s Hospital Los Angeles, United States of America

## Abstract

Most annexins are calcium-dependent, phospholipid-binding proteins with suggested functions in response to environmental stresses and signaling during plant growth and development. They have previously been identified and characterized in *Arabidopsis* and rice, and constitute a multigene family in plants. In this study, we performed a comparative analysis of annexin gene families in the sequenced genomes of Viridiplantae ranging from unicellular green algae to multicellular plants, and identified 149 genes. Phylogenetic studies of these deduced annexins classified them into nine different arbitrary groups. The occurrence and distribution of bona fide type II calcium binding sites within the four annexin domains were found to be different in each of these groups. Analysis of chromosomal distribution of annexin genes in rice, *Arabidopsis* and poplar revealed their localization on various chromosomes with some members also found on duplicated chromosomal segments leading to gene family expansion. Analysis of gene structure suggests sequential or differential loss of introns during the evolution of land plant annexin genes. Intron positions and phases are well conserved in annexin genes from representative genomes ranging from *Physcomitrella* to higher plants. The occurrence of alternative motifs such as K/R/HGD was found to be overlapping or at the mutated regions of the type II calcium binding sites indicating potential functional divergence in certain plant annexins. This study provides a basis for further functional analysis and characterization of annexin multigene families in the plant lineage.

## Introduction

Annexins are an evolutionarily conserved multigene family of Ca^2+^-dependent and phospholipid binding proteins [1]. They are a primitive group of proteins, dating their existence back ∼1–1.5 billion years ago in the unicellular protist, *Giardia lambia* [2]. The distribution of these proteins occurs widely in plants, animals and microorganisms [3], [4], and the early studies in plants indicated that they comprise a small and relatively simple gene family in maize [5], bell pepper [6] and tobacco [7]. Subsequently, with the availability of whole genome sequences, a total of eight and ten annexin genes, were identified in *Arabidopsis* and *Oryza sativa* L. ssp. *japonica* cv. Nipponbare, respectively [8], [9].

Studies on annexin gene structures have revealed conserved exon-intron positions with variable intron number among the different groups of organisms [10], [11]. Previous studies have indicated that duplication events might have caused the divergence and expansion of annexin genes in several groups of organisms including invertebrates, vertebrates, protists and plants [4], [12]. They represent a monophyletic cluster and were classified as a separate plant-specific family (D) among the five families representing vertebrates (A), invertebrates (B), fungi and some unicellular eukaryotes (C) and protists (E). Up to 17 subfamilies of annexins have been classified in plants and comparative analysis of plant annexins by phylogenetic relationship also showed their relatedness within the plant kingdom [4], [13]. Phylogenetic analysis of annexins of *Arabidopsis* and rice led to the identification of orthologous sequences [14], and conserved gene structures were observed among *Arabidopsis* and mustard (*Brassica juncea*) annexins, except for annexin 1 [15]. The expression patterns of plant annexin genes are often cell or tissue-specific and are regulated developmentally or by various biotic and abiotic stresses [3]. Several lines of evidence based on genetic and transgenic approaches have indicated that annexins play a significant role in protecting plants from both abiotic and biotic stresses [16]–[19].

Structurally, vertebrate annexins typically contain four similar domains in the carboxy-terminal end. Each domain contains a characteristic type II motif for binding calcium ions, represented as GxGT-(38 residues)-D/E, which is known to be important for phospholipid binding [20]. They also contain a variable long amino-terminal region that harbors sites for post-translational modifications and protein-protein interactions [4]. Biochemical analysis has shown that plant annexins including maize, wheat, bell pepper and *Mimosa* exhibit Ca^2+^-dependent phospholipid membrane binding [21]–[24]. Recent crystal structure studies indicated that Ca^2+^-dependent phospholipid binding of cotton annexin (GhANN1) occurs via domains I and IV [25]. Plants annexins have a small amino-terminal region and carboxy-terminal core domains, which are thought to be sites of post-translational modifications [26]. *In silico* analysis of rice and mustard annexins also identified many amino acid residues that might be the targets for post-translational modifications [9], [15]. Post-translational modification of AnnAt1 by phosphorylation modulated its *in vitro* peroxidase activity [27] while, *S*-glutathionylation caused a decrease in Ca^2+^-binding affinity thereby affecting membrane interaction [18]. A rice annexin, Os05g31750 was shown to interact with various kinases including MAPKK suggesting its involvement in Ca^2+^-dependent MAPK signaling [28]. In addition, a recent report showed that the Ca^2+^-dependent interaction of AnnAt1 and AnnAt4 regulate drought and salt stress responses in *Arabidopsis* [29]. Theoretical molecular docking studies have indicated that mammalian annexins may interact with C2 domain-containing proteins via a K/H/RGD motif [30]. Certain plant annexins also have a K/H/RGD motif that could allow them to interact with protein ligands containing C2 domains that are involved in plant signaling pathways [3], [9].

**Table 1:** Annexin genes identified from 16 sequenced plant genomes.

Lineage	Organism	Genome size (Mb)	No. of predicted genes	No. of annexin genes
Algae	*Micromonas* sp. RCC299	21.0	10,056	1
	*Ostreococcus tauri*	12.6	7,892	1
Moss	*Physcomitrella patens*	500	35,938	7
Lycophytes	*Selaginella moellendorffii*	100	22,285	5
Gymnosperms	*Picea sitchensis*	NA	NA	3
Dicots	*Arabidopsis thaliana*	120	25,498	8
	*Medicago truncatula*	500	19,000	10
	*Populus trichocarpa*	485	45,555	12
	*Vitis vinifera*	490	30,434	14
	*Carica papaya*	372	24,746	12
	*Glycine max*	1,100	46,430	22
	*Cucumis sativus*	367	26,682	11
Monocots	*Oryza sativa*	389	37,544	10
	*Sorghum bicolor*	730	34,686	10
	*Zea mays*	2,800	32,000	12
	*Brachypodium distachyon*	272	26,500	11
Total				149

Thus far, only limited information of annexin gene families is available from *Arabidopsis,* mustard, rice and tomato [8], [9], [14], [15], [31]. The recent availability of whole genome sequences of various plant species in the public databases ranging from unicellular algae to multicellular plants provides an opportunity for detailed molecular, evolutionary and functional insights in relation to annexin gene families. In this study, we performed a genome-wide survey of annexin multigene families in 16 plant species. Comparative analyses were performed to determine their phylogenetic relationships, the gene organization with respect to exon-intron conservation and the role of gene duplications in expansion of gene families. This was followed by structural analyses of the annexin protein domains and the sequence motifs to better understand the functional role that these proteins might possess.

## Materials and Methods

### Identification of Annexin Multigene Families in Public Databases

Annexin multigene families were identified from 16 completely sequenced genomes representing the plant lineage (Viridiplantae) including members from unicellular green algae to multicellular plants (Table 1). The search was performed using “annexin” as a keyword in SUPERFAMILY (http://supfam.cs.bris.ac.uk/SUPERFAMILY/), Plaza (http://bioinformatics.psb.ugent.be/plaza/news/index) and Phytozome (http://www.phytozome.org) databases and the sequences were retrieved from the corresponding plant genome annotation resources and analyzed. Partial and redundant sequences were excluded. The sequences were obtained from species ranging from unicellular green algae-*Micromonas* sp. RCC299 (http://bioinformatics.psb.ugent.be/plaza/organism/view/Micromonassp.RCC299) and *Ostreococcus tauri* (http://genome.jgi-psf.org/cgi-bin/dispGeneModel?db=Ostta4&tid=24272); bryophyte (moss)-*Physcomitrella patens* (http://www.phytozome.net/physcomitrella); lycophyte (spike moss)-*Selaginella moellendorffii* (http://genome.jgi-psf.org/Selmo1/Selmo1.home.html); dicotyledonous angiosperms-*Arabidopsis thaliana* (http://www.arabidopsis.org/), *Medicago truncatula* (http://bioinformatics.psb.ugent.be/plaza/organism/view/Medicagotruncatula), *Populus trichocarpa* (http://bioinformatics.psb.ugent.be/plaza/organism/view/Populustrichocarpa), *Vitis vinifera* (http://bioinformatics.psb.ugent.be/plaza/organism/view/Vitisvinifera), *Carica papaya* (http://bioinformatics.psb.ugent.be/plaza/organism/view/Caricapapaya), *Glycine max* (http://bioinformatics.psb.ugent.be/plaza/organism/view/glycinemax) and *Cucumis sativus* (http://supfam.cs.bris.ac.uk/SUPERFAMILY/cgi-bin/gen_list.cgi?genome=CU). *The* monocotyledonous angiosperms included- *O. sativa* L. ssp. *japonica* cv. Nipponbare (http://rice.plantbiology.msu.edu/), *Sorghum bicolor* (http://bioinformatics.psb.ugent.be/plaza/organism/view/Sorghumbicolor), *Zea mays* (http://bioinformatics.psb.ugent.be/plaza/organism/view/Zeamays) *and Brachypodium distachyon* (http://supfam.cs.bris.ac.uk/SUPERFAMILY/cgi-bin/gen_list.cgi?genome=BD). A protein name search was performed against the NCBI protein database (http://www.ncbi.nlm.nih.gov/protein) to obtain annexin sequences from a gymnosperm, *Picea sitchensis*. The genomic coordinates and open reading frame (ORF) regions were obtained from the above genome sequence browsers. The deduced protein sequences of the annexin gene families from the 16 genomes were analyzed for conserved domains, calcium binding sites (CBS) and any additional motifs using ‘Simple Modular Architechture Research Tool’ (SMART, http://smart.embl-heidelberg.de) and Prosite (http://ca.expasy.org/tools/scanprosite/) databases, respectively.

**Figure 1:**
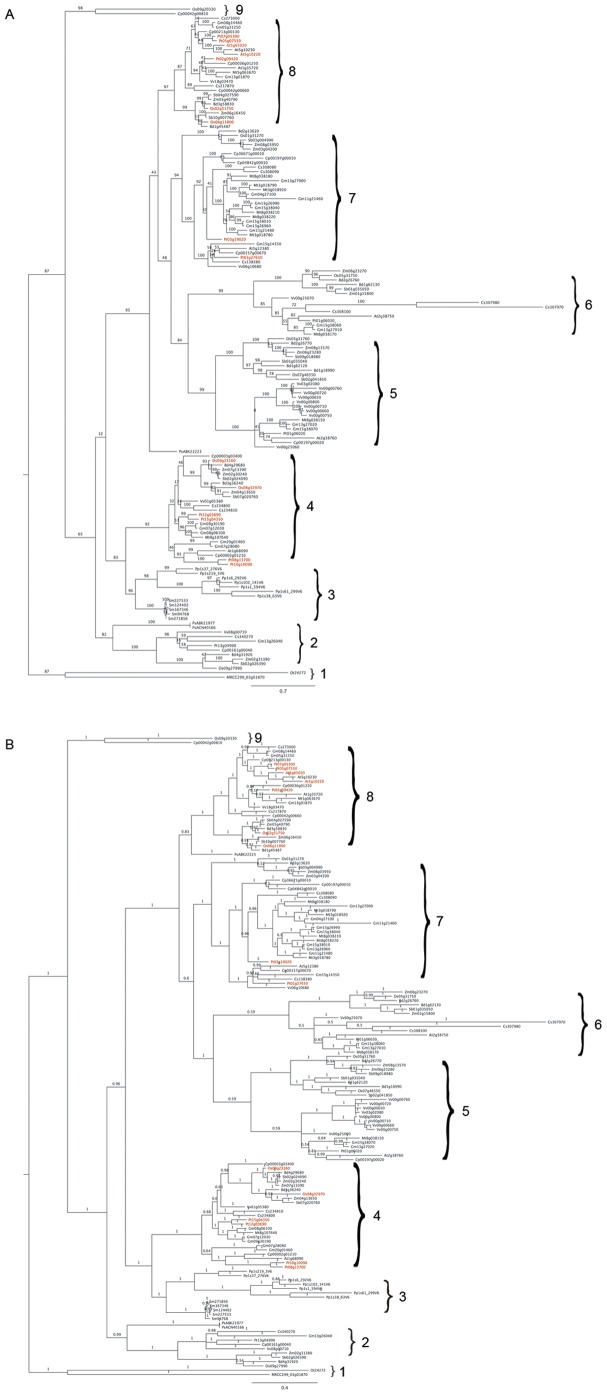
Phylogenetic trees showing the evolutionary relationship of 149 deduced annexin proteins from Viridiplantae by maximum likelihood method in RaxML (A) and Bayesian inference in MrBayes (B). The multiple sequence alignment was done for the deduced protein sequences using multiple sequence and structure alignment program PROMALS3D. The numbers at the nodes indicates the statistical support as obtained by 100 bootstrap RaxML replicates (likelihood of −51862.57) and Bayesian posterior probabilities (likelihood of −52897.07). The red lettered taxon labels represent the segmentally duplicated paralogous annexin sequences. We used algae as outgroup. The bar indicates amino acid substitutions per site.

### Phylogenetic Analysis

To investigate the evolutionary relationship of annexins among various plant species, the highly diverged deduced protein sequences that were identified from all the genomes were aligned in multiple sequence and structure alignment program PROMALS3D server (http://prodata.swmed.edu/promals3d) [32]. The alignment in Figure S1 was used to build a maximum likelihood (ML) tree by employing RAxML BlackBox (http://phylobench.vital-it.ch/raxml-bb/index.php) using Jones-Taylor-Thornton (JTT) substitution matrix model [33]. The bootstrap analysis was performed using 100 replicates and the branch length corresponded to phylogenetic distances. By using the same alignment, the phylogenetic tree was also inferred by Bayesian analysis implemented in MrBayes version 3.2 [34] using mixed amino acid models in default setting and ran for 7,500,000 generations and then used to estimate the posterior probabilities for each nodes. The phylogenetic trees were visualized using FigTree (http://tree.bio.ed.ac.uk/software/figtree/).

**Figure 2:**
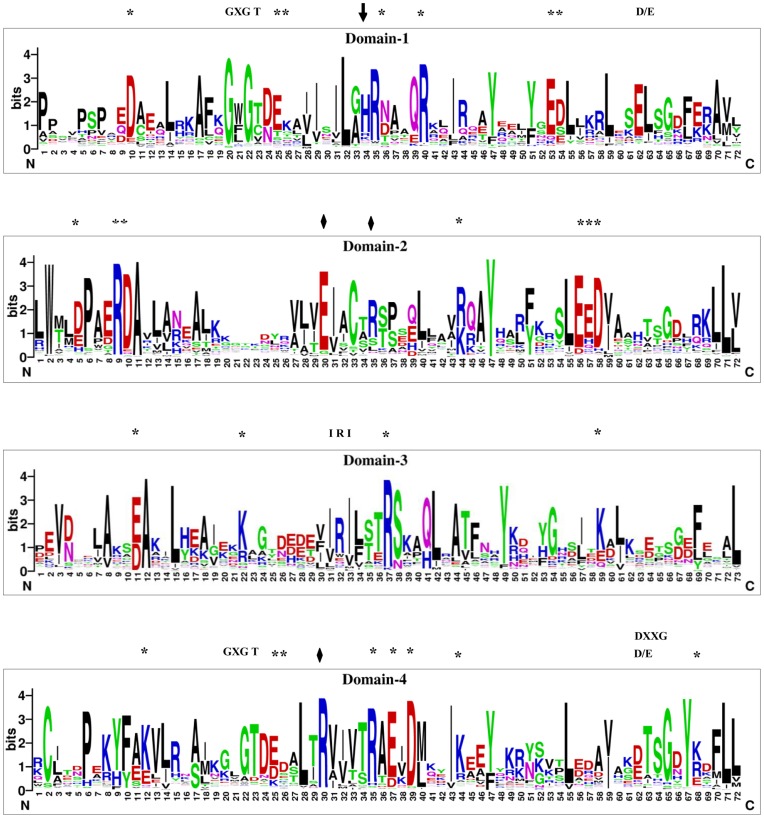
Sequence logos of four annexin domains. The sequence logos were generated by amino acid alignment of individual domains from 149 annexins using WebLogo. The taxon-specific indels were removed to optimize the alignments. The height of letter designating the amino acid residue at each position represents the degree of conservation. The GXGT and D/E, IRI and DXXG motifs are represented on the top of each plot. The conserved His residue in the heme motif is indicated by an arrow. The residues thought to be involved in ion channel activity are represented as diamonds. Asterisks (*) indicated the conserved residues observed in the alignment of 149 annexins. The numbers on the x-axis represent the sequence positions in annexin domains. The y-axis represents the information content measured in bits.

### Analysis of Annexin Genes for Exon-intron Structure

The exon-intron structures of annexin genes were analyzed in the plant lineage ranging from non-vascular to vascular land plants (*Physcomitrella*, *Selaginella*, *Arabidopsis*, and rice) by comparing the genomic and coding or cDNA sequence information obtained from aforementioned genome databases. The annexins from green algal species *Micromonas* sp. and *O. tauri* were not included in the analysis as the corresponding genomic sequences are intronless. Intron phases in between exon-intron junctions were also obtained by using the online tool, Gene Structure Display Server (http://gsds.cbi.pku.edu.cn/).

**Figure 3:**
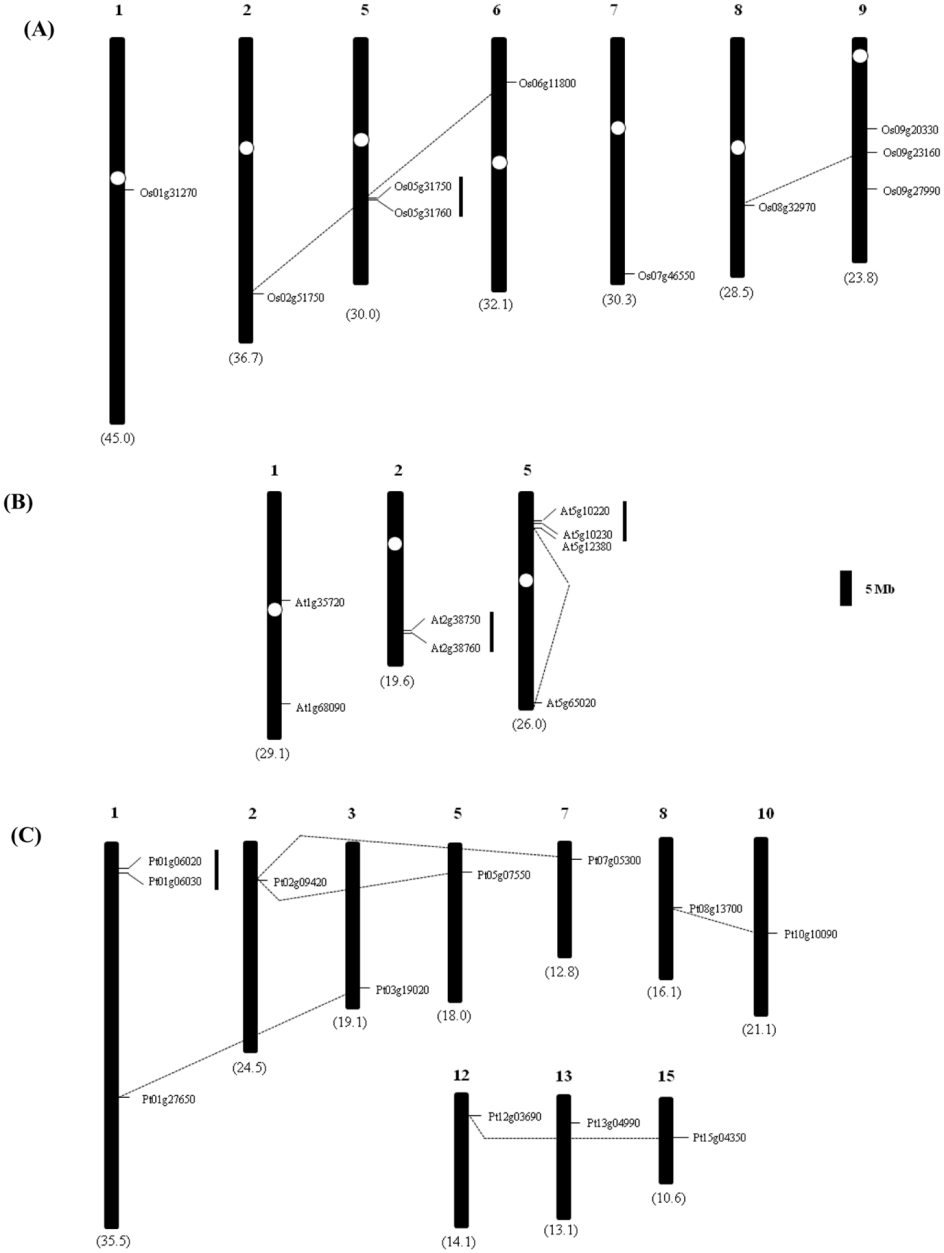
Chromosomal localization of annexin genes in (A) rice, (B) *Arabidopsis* and (C) poplar. The number indicated at the top represents the chromosome number. The tandemly duplicated genes are indicated as vertical lines and the segmental duplicated genes by dotted lines. The scale represents a 5 Mb chromosomal distance. The numbers in brackets represents the corresponding chromosome size.

### Chromosomal Distributions and Duplication of Annexin Genes

The location of annexin genes was determined based on their physical positions on chromosomes corresponding to their locus numbers in the Rice Genome Annotation Project (RAP) database for rice (http://rice.plantbiology.msu.edu/), TAIR for *Arabidopsis* (http://www.arabidopsis.org/) and Plaza for *Populus* (http://bioinformatics.psb.ugent.be/plaza/organism/view/Populustrichocarpa). The duplication of annexin genes on segmentally duplicated regions was determined using “Paralogons in *Arabidopsis*” (http://wolfe.gen.tcd.ie/athal/dup), segmental genome duplication database for rice (http://rice.plantbiology.msu.edu/segmental_dup/500kb/segdup_500kb.shtml) at a maximum length distance permitted between collinear gene pairs of 500 kb and the information on chromosome mapping on collinear regions at Plaza database for poplar and soybean (http://bioinformatics.psb.ugent.be/plaza_v1/dotplot/). The annexin genes separated by a maximum of two to five genes were identified as tandemly duplicated regions.

**Table 2:** Distribution of annexin genes in duplicated genomic regions in various plant species.

Organism	Segmentally duplicated genes (%)	Tandem duplicated genes (%)
*A. thaliana*	25	50
*M. truncatula*	0	70
*P. trichocarpa*	75	17
*V. vinifera*	0	64
*C. papaya*	0	33
*G. max*	50	50
*O. sativa*	40	20

The segmental duplicated annexin genes for *Arabidopsis* and rice were obtained from (http://wolfe.gen.tcd.ie/athal/dup) and (http://rice.plantbiology.msu.edu/segmental_dup/500kb/segdup_500kb.shtml), respectively. For poplar and soybean, the segmental duplications were identified based on the chromosome mapping in collinear regions in Plaza database (http://bioinformatics.psb.ugent.be/plaza_v1/dotplot/). The annexin genes separated by two to five genes were identified as tandemly duplicated genes.

### Estimating the Age of Duplicated Paralog Gene Pairs

In order to calculate the age of segmentally duplicated annexin paralogs, the pairwise alignment of annexin gene pairs from *Arabidopsis*, poplar and rice was performed using Clustal X 1.83. The duplication age was estimated by number of synonymous substitution per synonymous site (*K*s). The *K*s values of the duplicate annexin gene pairs were estimated by the program K-Estimator 6.1 [35]. Based on the synonymous substitutions per year (λ) of 1.5×10^−8^ for *Arabidopsis* [36], 6.5×10^−9^ for rice [37] and 9.1×10^−9^ for poplar [38] and by substituting the calculated *K*s values, the approximate age of duplicated events of the duplicate annexin gene pairs was estimated (T = *K*s/2λ). The selection pressure for these duplicate annexin paralog gene pairs was calculated as *K*a/*K*s ratio.

**Table 3:** Estimation of the age of segmentally duplicated annexin genes in *A. thaliana*, *O. sativa* and *P. trichocarpa*.

Organism	Duplicated annexin gene 1	Duplicated annexin gene 2	*K*s	*Ka/K*s	Age (MYA)
***A. thaliana***	*At5g10220*	*AT5G65020*	0.62125	0.222	20.70
***O. sativa***	*LOC_Os02g51750*	*LOC_Os06g11800*	0.63143	0.154	48.57
	*LOC_Oso9g23160*	*LOC_Os08g32970*	1.18455	0.194	91.07
***P. trichocarpa***	*PT02G09420*	*PT05G07550*	0.95465	0.163	52.40
	*PT02G09420*	*PT07G05300*	1.04892	0.153	57.63
	*PT08G13700*	*PT10G10090*	0.18138	0.414	9.90
	*PT01G27650*	*PT03G19020*	1.15991	0.237	63.73
	*PT12G03690*	*PT15G04350*	0.31043	0.256	17.0

## Results and Discussion

### Identification of Annexin Multigene Family in Plants

Annexins are found in various taxa including invertebrates, vertebrates, plants, fungi and some lower organisms such as yeast and prokaryotes [1], [4], [39]. The first evidence for the existence of plant annexins as multigene families has come from the model plant, *Arabidopsis* [8]. Though considerable progress has been made during the past decade in the characterization of plant annexins, studies on multigene families are still scarce except for their recent identification and characterization in mustard, rice and tomato [3], [9], [15], [31], [40]. In order to identify annexin multigene families in different plant species, a genome-wide search was performed in 16 different genomes in Viridiplantae. The sequences were surveyed by annotation search from complete draft genome sequences except for the gymnosperm, *P. sitchensis* whose complete sequence information is not available yet. Because sequences were identified by annotation search it is unlikely that we have obtained all plant annexin sequences currently available. A total of 378 sequences were identified in the Superfamily, Plaza and Phytozome databases. Further analysis of their respective genome resources resulted in 149 non-redundant annexins after excluding partial and redundant sequences based on 100% sequence similarity which were subsequently analyzed for conserved domain architecture in SMART database. The sequence features of all the retrieved annexins are presented in Table S1. Our analysis shows that the number of annexin (Anx) domain containing members ranged from 1 to 22 across the different plant species, existing as multigene families except for annexins from green algae (Table 1). In addition, a survey of genome databases suggests that alternative splicing (AS) events that could increase proteome diversity might occur predominantly in monocot annexins with the exception of sorghum. *In silico* analysis of rice annexins indicates that two primary annexin transcripts (*Os09g23160* and *Os02g51750*) might undergo AS generating additional transcripts [9]. The maize genome contains a maximum of five annexin genes that may undergo AS, while, *Brachypodium* has one. In dicots, the *Arabidopsis* annexin (*At5g65020*; *AnnAt2*) may undergo AS to generate additional transcripts (data not shown). Reports from vertebrates showed that annexins ANXA6 and ANXA7 undergo AS and their corresponding isoforms were regulated in a tissue-specific manner [41], [42], whereas ANXA11 exhibited isoform-specific vesicle formation or calcium-dependent binding to calcyclin, a member of S100 protein family [43], [44].

**Figure 4:**
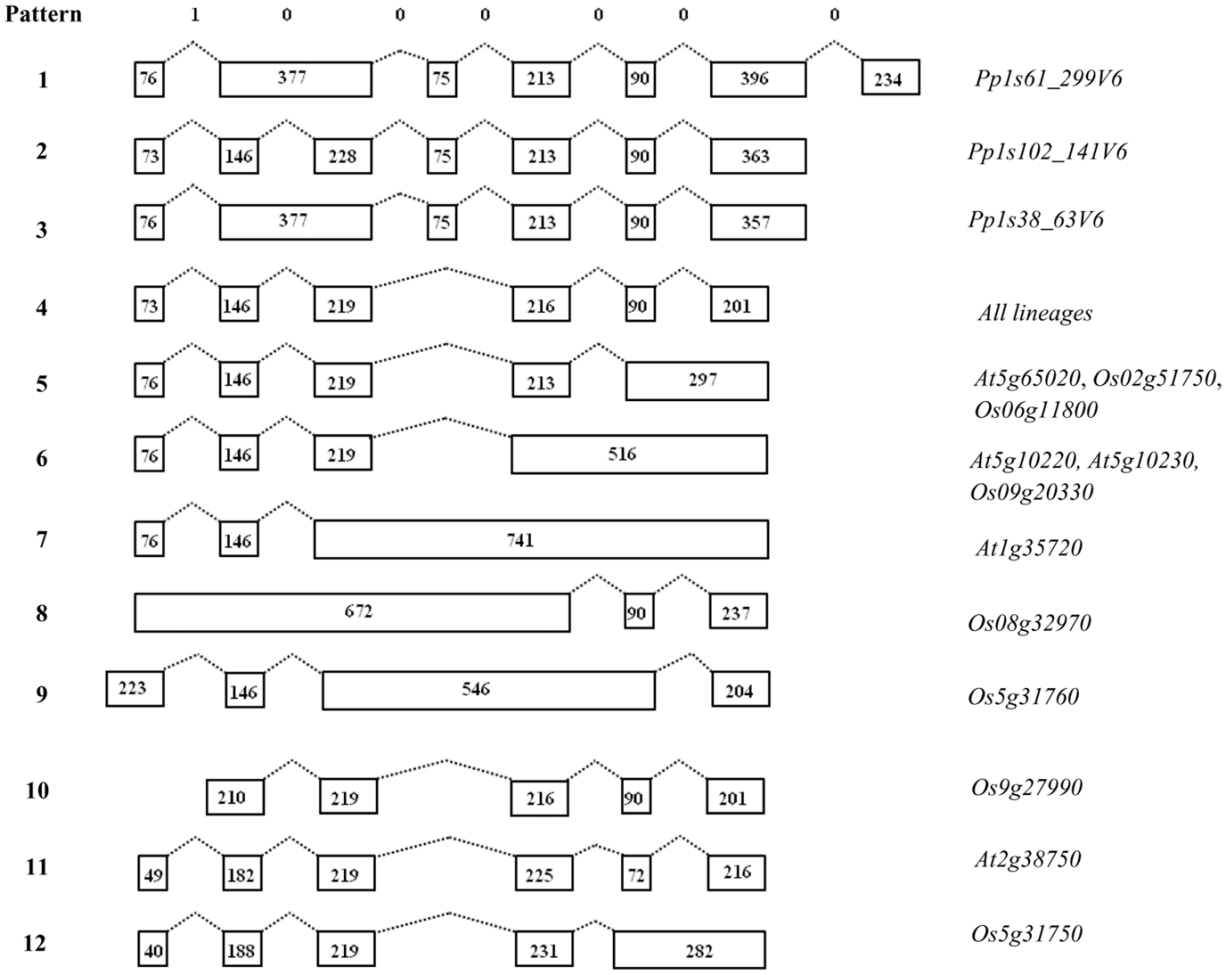
Exon-intron organizations of annexin genes from genomes of moss, spike moss, *Arabidopsis* and rice. Exons and introns are indicated as open boxes and dotted lines respectively. The intron phases are depicted as 0 and 1 at the top. Numbers at the left show intron-exon patterns and those at the right show the type of pattern observed in different genes across genomes. Numbers within the boxes represent exon sizes. The exons and introns are not drawn to scale.

### Origin of Plant Annexins

The earliest annexins in photosynthetic organisms possibly originated ∼one billion years ago in green algae during their evolution into land plants [4], [45]. In the division Chlorophyta of green algae, the genomes of *Micromonas* sp. and *O. tauri*, which belong to the class Prasinophyceae, possess one annexin gene each. Our search for annexins in the model organism of the Chlorophycean member, *Chlamydomonas reinhardtii* did not result in any positive match. It has been suggested that members of Prasinophyceae were the primitive species in Viridiplantae from which all other green algae and land plants have evolved [46]. This suggests that the origin of annexin genes in land plants could be traced back to the primitive Prasinophytes. However, the availability of complete genome sequence of the Charaophycean green algae, believed to be the closest relatives of land plants, might provide detailed information on the divergence of plant annexin genes [47]. The expansion of annexin gene family was evident during the colonization into land plants by early bryophytes ∼450 million years ago (MYA) [48], [49] prior to the divergence of monocot and dicots (angiopserms) ∼150 MYA. The annexin multigene family appeared to expand with the complexity of the genome (Table 1), possibly by duplication events, which is consistent with the findings of Vogel and Chothia [50]. For example, the non-vascular bryophyte, *P. patens* has seven annexin genes, which might have resulted through duplication. The expansion of annexin gene family continued with soybean exhibiting 22 paralogous gene sequences representing ∼15% of the total 149 identified annexins, which might be due to recent genome duplications that occurred ∼13 MYA [51]. The conservation and expansion of plant annexins during the course of evolution implies that this multigene family may have important physiological roles during plant adaptation to environment.

**Figure 5:**
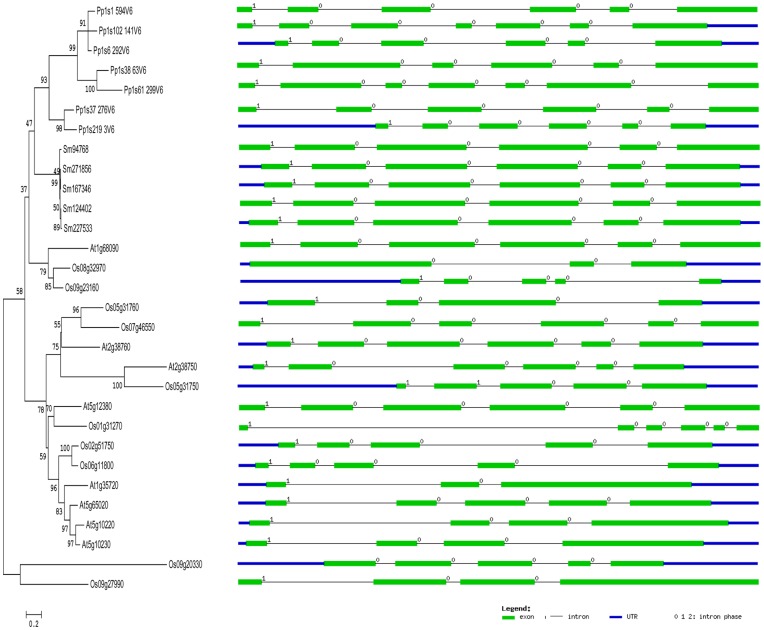
Gene structures of annexins from four representative genomes (moss, spike moss, *Arabidopsis* and rice) in plant lineage. The intron phases are highly conserved in all the genomes and represented in the maximum likelihood phylogeny tree. As shown in the legend, the intron phases in between exon-intron junctions are given as 0 and 1, exons are represented by green filled boxes, introns by black lines and untranslated regions (UTR) by blue filled boxes. The scale bar represents 0.2 amino acid substitutions per site. The gene structures were drawn using online tool Gene Structure Display Server (http://gsds.cbi.pku.edu.cn/).

### Phylogenetic Relatedness of Annexin Gene Families

Previous phylogenetic analyses, based on studies with more limited datasets in angiosperms have indicated that plant annexins exist as a separate monophyletic cluster (Plant-specific family type-D), when compared with annexins outside the plant-lineage [4], [11], [14], [15]. In order to gain better insights into the evolutionary relationships, we performed phylogenetic analysis of 149 annexins identified in Viridiplantae. Given its high sequence divergence, we first aligned all these deduced annexins in the multiple sequence and structure alignment program PROMALS3D to generate phylogenetic trees using two methods, maximum likelihood bootstrap using RaxML and Bayesian inference (BI) using MrBayes (Figure 1A,B). The tree classified all of the 149 deduced protein sequences into nine different arbitrary groups of related protein clades designated as 1 to 9. Both methods resulted in trees with similar topologies and statistical support at each of the nodes represented by bootstap values of 82–99% (Figure 1A) and posterior probabilities of 0.59–1 (Figure 1B) among the groups.

**Figure 6:**
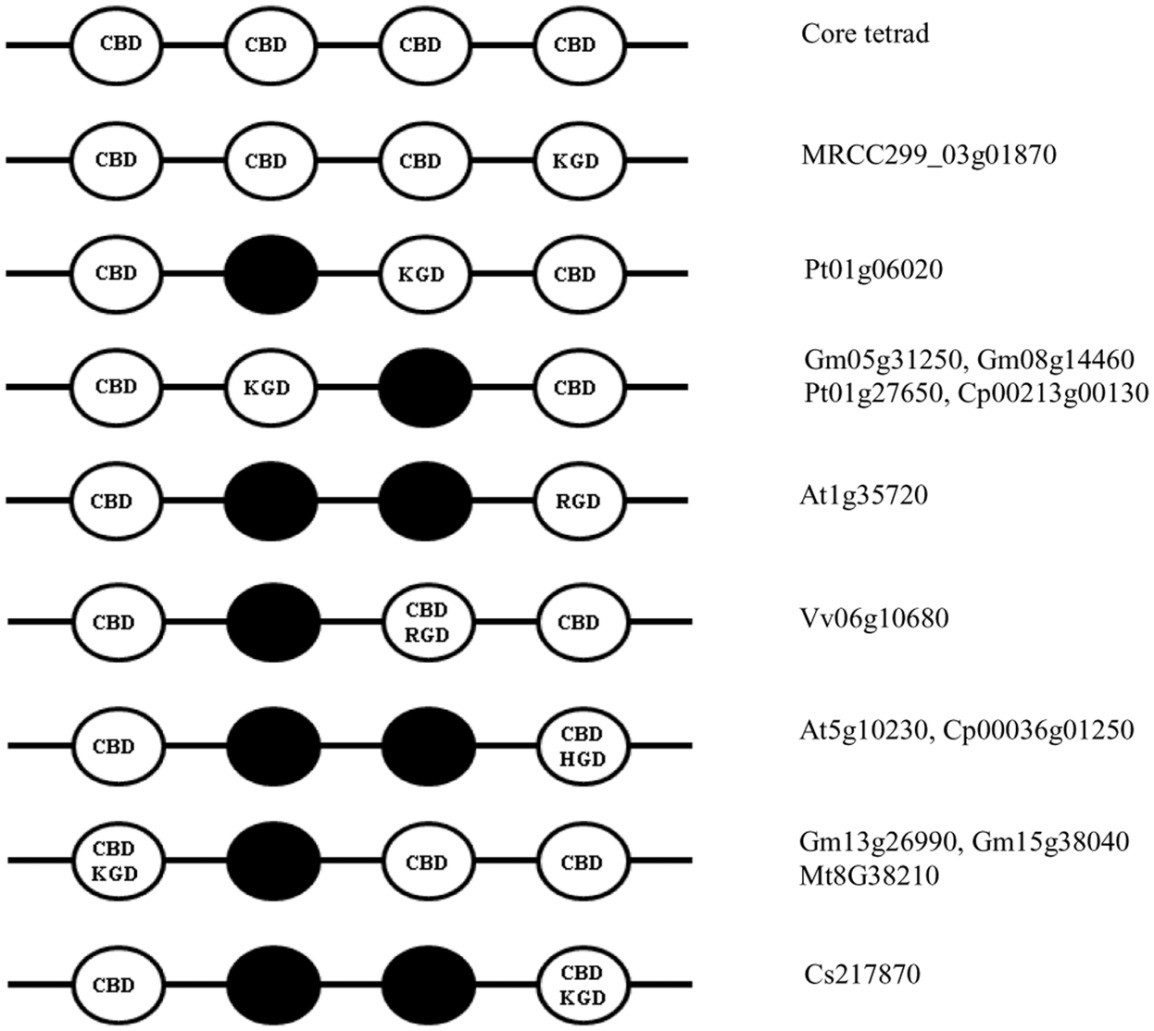
Domain organizations showing the presence or absence of CBS. Anx domain structures showing CBS and K/H/RGD motifs. The occurrence of these motifs in different genomes was analyzed from all deduced proteins. Dark circles represent the absence of CBS or K/H/RGD motifs. Domain structures are not drawn to scale.

Most of the groups were dominated by annexins from angiosperms (monocots and eudicots), except for groups 1 and 3. Group 1 was assigned to annexins from unicellular green algae, *O. tauri* and *Micromonas* sp. Annexins from non-vascular land plants such as the bryophyte *Physcomitrella* and vascular lycophyte *Selaginella* were classified in group 3 indicating that the annexins in this group shared a common ancestor after their divergence probably from the Charophycean green algae [47]. Group 2 was represented by annexins from multicellular land plants, such as gymnosperms and angiosperms indicating that they shared a common ancestor before their divergence ∼300–400 MYA [52]. Similarly, 64% of grape vine annexins were present in group 5, while most of the legume-specific annexins were clustered in group 7. Annexins from the angiosperm flowering plants were classified in groups 4, 5, 6, 7 and 8, suggesting that this group showed conservation for ∼150 MYA before diverging into monocots and dicots. The phylogenetic analysis (Figure 1A,B) indicated that several tandem duplicated genes pairs distributed in groups 5 and 6 as orthologous sequences. Additionally, several paralogous sequences of *Arabidopsis*, rice and poplar are present in segmentally duplicated regions and found to be distributed among groups 4, 7 and 8 (Figure 1A,B red taxon labels), which we discussed later in more detail.

### Diversity of Calcium Binding Sites in Plant Annexins

In contrast to vertebrates, plant annexins lack a long N-terminal region and predicted CBS in the second and third domains [4]. Also, one of the functions of plant annexins is Ca^2+^-dependent phospholipid-binding, which occurs through the first and fourth annexin domains through CBS [25]. Analysis of all plant annexins in each of the nine groups in the phylogenetic tree (Figure 1A,B) for the presence of predicted CBS in the core tetrad domains indicated that these proteins might have different calcium binding specificities. Our analysis revealed that six plant annexins in group 3 (one member from *Physcomitrella*, Pp1s37_276V6 and all the five members of *Selaginella*) have a core tetrad CBS similar to that found in vertebrate annexins [4]. The presence of CBS in all four domains of these six annexins may indicate their Ca^2+^-dependent phospholipid membrane-binding through a Ca^2+^-bridging mechanism similar to that observed in mammalian annexin A5 [53]. In group 4, annexins from angiosperms contain exclusively three CBS except for soybean (Gm08g06100) and *Arabidopsis* (At1g68090) annexins, which contain two CBS. The presence of a higher number of binding sites in group 4 annexins suggests that these land plants once acquired CBS from their ancestors, but after the divergence from green algae. Among the 149 plant annexin proteins, 15% were classified in group 8 possessing two CBS found in the first and fourth domains, which is a characteristic feature of plant annexins. In group 5, all the *Vitis-*specific annexins lacked CBS, while other annexins within the same group possess 1 and 2 CBS. All the annexins in group 6 also lacked the critical amino acid residue for binding calcium ions. The deduced annexin proteins having none or one CBS were found in group 2. The absence of CBS in group 6 and *Vitis* annexins suggests that these proteins might bind phospholipids independent of calcium and might have different biological functions. Ca^2+^-independent phospholipid membrane-binding has been shown for plant annexins [13]. The presence or absence of the CBS in annexin tetrad domains might affect interactions with other annexins. A study by Huh et al. [29] showed that mutations within CBS in both AnnAt1 and AnnAt4 affected their physical interaction *in vitro* whereas, their *in vivo* interaction was shown to be regulated by response to drought and salt stresses.

Since all plant annexins are devoid of additional predicted non-annexin functional domains and annexins of land plants are multigene families occurring primarily with intradomain combinations, detailed studies in various plant species are required to gain a better insight into their (non)-redundant function(s). Comparative sequence analyses for the conservation of amino acid residues in each of the four annexin domain sequences of the 149 plant annexins were performed based on the structural alignment obtained through PROMALS3D (Figure S2A–D). The sequence logos of each of the annexin domains of ∼70 aa were generated using WebLogo program (http://weblogo.berkeley.edu/) to validate the conservation of residues in the domains (Figure 2). The taxon-specific indels were removed to optimize the alignments. We observed the relative conservation of two glycine residues and CBS in the first and fourth annexin domains, IRI-motif for binding actin in the third domain, DXXG-motif for GTPase activity in the fourth domain [8] and the residues thought to be involved in ion channel activity [3]. The histidine residue essential for maintaining the secondary structure of the annexin protein is also present [18]. In addition, several highly conserved charged residues are found in all the annexin domains. It is important to note that although this analysis identified conserved structural features in plant annexins likely to have functional significance, there are many non-conserved individual features responsible for functional diversity within the annexin gene family that are not highlighted by this approach.

### Chromosomal Distribution and Duplication of Annexin Family Genes

To determine the chromosomal distribution of annexin family genes in *Arabidopsis*, rice and poplar, the 5′- and 3′- coordinates of each of the gene models were obtained from their respective genome databases. The 10 annexin members of rice were located on chromosomes 1, 2, 5, 6, 7, 8 and 9 (Figure 3A), while the eight annexin genes of *Arabidopsis* were distributed over chromosomes 1, 2 and 5 (Figure 3B). As shown in Figure 3C, all 12 annexin genes of poplar were localized on 10 chromosomes (chromosomes 1, 2, 3, 5, 7, 8, 10, 12, 13 and 15). In *Arabidopsis*, chromosome 5 contained the largest number of annexin genes (four out of eight genes, 50%), while in rice three annexins were located on chromosome 9, two on chromosome 5 and one each on chromosomes 1, 2, 6, 7 and 8. In poplar, chromosome 1 has three annexin genes while the other genes were distributed uniformly, one on each chromosome.

We further investigated whether duplication events contributed to annexin gene family expansion (Figure 3A–C). In *Arabidopsis*, rice and poplar genomes, 75%, 60% and 92% of the annexin genes had undergone both tandem and segmental duplications, respectively. In *Arabidopsis*, two gene clusters representing 50% (four out of eight) are located in tandem on chromosomes 2 and 5. Similarly, one gene pair each in rice and poplar genomes was tandemly duplicated and found on chromosome 5 (20%) and chromosome 1 (17%), respectively. The tandem duplicate annexin gene pair members of *Arabidopsis* (*At2g38760* and *At2g38750*), poplar (*Pt01g06020* and *Pt01g06030*) and soybean (*Gm13g27020* and *Gm13g27010*; *Gm15g38070* and *Gm15g38060*) placed in groups 5 and 6 in the present analysis were direct orthologues of rice (*Os05g31760* and *Os05g31750)*, *Sorghum* (*Sb01g035040* and *Sb01g035050*) and *Brachypodium* (*Bd1g62120* and *Bd1g62130*; *Bd2g26770* and *Bd2g26760*) annexins, respectively (Figure 1A,B). These tandemly duplicated annexin members showed a sequence identity of 29–35% at the amino acid level (data not shown) indicating more divergence in protein sequence and function(s). Another tandemly duplicated annexin gene pair from *Arabidopsis* (*At5g10220* and *At5g10230*) placed in group 8 showed 82% identity at the amino acid level and was expected to show less protein divergence and function, possibly due to a recent-lineage specific duplication [14]. This feature may also be possible with some of the tandemly duplicated members in other groups of the phylogenetic tree (Figure 1A,B). The physical distribution of genes on duplicated chromosomal segments was also compared. In *Arabidopsis*, two out of the eight genes are located on intra-segmental duplicated regions of chromosome 5 (*At5g10220* and *At5g65020*). In rice, four out of 10 members are localized to segmentally-duplicated regions. The gene pair (*Os02g51750* and *Os06g11800*) is located on a duplicated segment between chromosomes 2 and 6, while the other gene pair (*Os08g32970* and *Os09g23160*) on chromosomes 8 and 9. The segmental duplications of annexin genes in *Arabidopsis* and rice might have occurred in the corresponding genomes due to large-scale segmental duplication events during evolution; at least four large-scale duplications might have occurred during the divergence of monocots and dicots ∼100–200 MYA [54]–[56]. The genome of poplar contained a maximum of five gene pairs on segmentally duplicated regions, in which a single annexin gene *Pt02g09420* paired with two genes (*Pt05g07550* and *Pt07g05300*) on segmentally duplicated regions between chromosomes 5 and 7. Another gene pair (*Pt08g13700* and *Pt10g10090*) is located on duplicated regions of chromosomes 8 and 10, and the remaining two gene pairs (*Pt01g27650* and *Pt03g19020*; *Pt12g03690* and *Pt15g04350*) are distributed over chromosomes 1 and 3; 12 and 15, respectively. In the genome of poplar, 75% of the annexin genes are segmentally duplicated and this might be due to the Salicoid duplication that occured ∼65 MYA [57]. We also observed that the number of annexin genes in poplar is ∼1.5 times more than that of *Arabidopsis.* This is consistent with the previous findings where the total number of protein-coding genes in *Populus* is 1.4 to 1.6 times more than that of *Arabidopsis* [57]. A summary of the tandemly and segmentally duplicated annexin genes from the various genomes is shown in Table 2. It is noteworthy to mention that members of annexin gene families in segmental duplicated regions of all the organisms analyzed in this study shared 60–85% identity at the amino acid level (data not shown). The genome of *Selaginella* did not show any evidence of duplication or polyploidy [58].

We further estimated the approximate age of segmentally duplicated annexin paralogous gene pairs from *Arabidopsis*, rice and poplar (Table 3). The number of synonymous substitutions per synonymous site (*K*s) is usually used to estimate the evolutionary age of duplicate gene pairs [59]. The nucleotide sequences of duplicated gene pair-*At5g10220* and *At5g65020* from *Arabidopsis* showed a *K*s value of 0.62125 indicating that its duplication might have occurred 20.7 MYA consistent with its divergence from the genus *Brassica* ∼12–20 MYA [60], but after the emergence of crucifers ∼24–40 MYA [61]. Similarly, the segmentally duplicated pair- *Os08g32970* and *Os09g23160* in rice was estimated to have evolved ∼91.07 MYA, subsequent to the divergence between monocots and dicots (100–200 MYA), while another gene pair-*Os02g51750* and *Os06g11800* was due to a recent duplication at ∼48.57 MYA after the divergence of poaceae from the common ancestor ∼55–70 MYA [56]. In the genome of poplar, 75% of the annexin genes are segmentally duplicated and this might have resulted between 9.9 to 63.73 MYA. The gene pair, *Pt08g13700* and *Pt10g10090,* might have undergone a recent duplication corroborating a recent duplication of poplar ∼8–13 MYA, while the other gene pairs were duplicated after the separation of poplar from salix ∼60–65 MYA [57], [62].

It has also been suggested by Lynch and Conery [38] that during the process of evolution, the duplicate genes might have undergone a loss of function (nonfunctionalization), acquired a new function by natural selection (neofunctionalization) or show the ancestral gene function (subfunctionalization). Hence, it can be speculated that the duplicated annexin genes in plants might have evolved and expanded due to neofunctionalization or subfunctionalization during their adaptation to land and survival under harsh environmental stress conditions. This holds true especially for the bryophyte, *Physcomitrella,* during its colonization of land [48] as 33% of its annexin genes are segmentally duplicated (data not shown).

The *K*a/*K*s ratio (synonymous substitutions to non-synonymous substitutions) indicates selection pressure of the duplicated genes [63]. If *K*a/*K*s = 1, the gene pair is said to be undergoing neutral evolution, while, a *K*a/*K*s>1 and <1 indicates the positive and purifying selections, respectively. All the segmentally duplicated annexin paralog gene pairs from *Arabidopsis*, rice and poplar showed *K*a/*K*s<1, indicating a purifying selection.

### Structural Organization of Annexin Genes in Plants

Analysis of annexin gene structure for exon-intron organization in Viridiplantae revealed that the number of introns per gene varied from 0 to 8. Among all the genes analyzed, four intronless genes were observed in green algae (one each in *O. tauri* and *Micromonas* sp.) and monocot species, sorghum (*Sb07g020760*) and maize (*Zm04g13650*). Annexins from two dicot species, soybean (*Gm11g21460*) and grape vine (*Vv03g02080*) possessed a maximum of eight introns each.

Four representative genomes across the plant lineage that included moss (bryophyte), spike moss (lycophyte), *Arabidopsis* (dicot) and rice (monocot) were further analyzed for annexin gene structure organization (Table S2). The annexin gene sequences from a gymnosperm representative, *P. sitchensis,* were not included due to the non-availability of genomic sequence information. Annexins from green algae are intronless like the gene structure exhibited by the primitive *Giardia* annexins [11]. Comparative analysis of gene structures in these genomes resulted in 12 splicing patterns (Figure 4). Among all the patterns, the rice annexin, *Os09g27990,* lacked the conserved first exon. The splicing patterns-1, 2 and 3 were exclusively present in moss, *Physcomitrella* represented by a single annexin gene each (*Pp1s61_299V6*, *Pp1s102_141V6* and *Pp1s38_63V6*). The *Pp1s61_299V6* and *Pp1s102_141V6* genes showed seven exons interrupted by six introns, while *Pp1s38_63V6* was formed by the loss of intron 2 corresponding to pattern-2. Pattern-4 occurred by the loss of the fourth exon and, introns 3 and 4 corresponding to pattern-2. All genomes possessed this gene splicing pattern including the *Selaginella* annexins. The sizes of most of the exons and the number of introns were found to be highly congruent, and this gene structure is evident in 15 out of the 30 (50%) annexins studied, indicating the conserved nature of this splicing pattern in the plant lineage. Differential or sequential loss of introns corresponding to splicing pattern-2 resulted in the rest of splicing patterns. Splicing patterns-5 and 6 are present in *Arabidopsis* and rice annexins. Among the different splicing patterns observed, four are solely present in rice annexins (splicing patterns-8, 9, 10 and 12). Annexins from *Arabidopsis* also possessed specific gene organizations due to intron loss. For example, *At5g65020* (*AnnAt2)* is formed by the loss of last intron belonging to pattern-5, while the tandem duplicated gene pair, *At5g10220* (*AnnAt6*) and *At5g10230* (*AnnAt7)* resulted from the loss of third and fourth introns as in pattern-6, which is also exhibitied by the rice annexin, *Os9g20330*. The *At1g35720* (*AnnAt1*) in pattern-7 was formed by the sequential loss of the last three introns. This is probably a characteristic feature of a recent lineage-specific duplication [14], which was further supported by the observation that *AnnBj1* gene from a crucifer relative, *B. juncea* (Indian mustard) contains four introns rather than the two found in its *Arabidopsis* homolog, *AnnAt1* [15]. This difference in the gene structure between the orthologs might be due to the divergence of *Arabidopsis*-*Brassica* genomes that might have occurred ∼24 MYA [60]. Thus, it appears that intron loss might be a predominant factor in the evolution of annexin genes in land plants. Consistent with this, recent findings showed that the occurrence of intron loss in *Arabidopsis* and rice is 12.6 and 9.8 times more common than that of intron gains, respectively and that intron loss dominated the evolution of plants [64].

Analysis of annexin genes for intron phases in land plants including moss, spike moss and angiosperms (*Arabidopsis* and rice) revealed that their first exons are flanked by an intron in phase 1 (after the first base of a codon), while the rest of their exons are present in phase 0 (between codons) in the exon-intron junctions (Figure 5). The positions of introns in each group in the phylogeny and their phases with symmetric exons are well conserved indicating that all these annexin genes from the land plants might have a common ancestor. The conserved intron phases in the gene structure may have provided stability during evolution similar to that observed in vertebrate annexins [65].

### Other Significant Motifs in Plant Annexins

Apart from CBS, annexins showed the structural replacement of calcium-coordinating residues and contain 11% of annexin domains as a novel KGD or 10% as RGD motifs at AB and DE interhelical regions [39]. The RGD motif is a cell attachment sequence present in proteins in the extracellular matrix (ECM) and acts as a binding site for cell surface receptors such as integrins for signaling in cell adhesion [66]. Theoretical docking studies predicted that the KGD motif in mammalian annexins (ANX1, ANX5, ANX6 and ANXA13b) acts as a ligand for interaction with C2 domain containing proteins involved in signal transduction [30].

Based on the above studies, we analyzed plant annexins containing four annexin domains for the presence of K/R/HGD motifs (Figure 6). The KGD motif was found adjacent to the mutated CBS in the second domain of annexins from soybean (Gm05g31250 and Gm08g14460), poplar (Pt01g27650) and papaya (Cp00213g00130), and in the third domain of poplar annexin (Pt01g06020). It also existed as an overlapping sequence in the first domain of annexins from legumes (Gm13g26990, Gm15g38040 and Mt8g38210) and in the fourth domain from *Cucumis* (Cs217870). Similarly, the RGD motif is present adjacent to the CBS in the fourth domain of *Arabidopsis* annexin 1 (At1g35720) and as an overlapping sequence in the third domain of *Vitis* annexin (Vv06G10680). Overlapping sequence in the form of HGD was also present in the fourth domain of annexins from *Arabidopsis* (At5g10230) and papaya (Cp00036g01250). So far attempts to identify plant integrin-like proteins that might bind to RGD motifs have been unsuccessful [67]–[69]. However, the RGD motif in Cardosin A was shown to interact with the C2 domain of phospholipase Dα [70].

The *Micromonas* annexin also possesses a KGD motif adjacent to the mutated CBS in the fourth domain, whereas the *O. tauri* annexin was devoid of this motif. However, an annexin from the related species, *O. lucimarinus,* has two RGD motifs in the second and fourth domains, and a HGD motif in the amino terminal end [30]. ScanProsite analysis showed that the annexin in *O. tauri* instead carries a proline-rich region (residues 344–425) at the carboxy terminus with penta domains of the heptapeptide PPPQGYA. Tandemly domained proline-rich sequences in general are known to be involved in protein-protein interactions [71]. Two mammalian annexins, A7 and A11 that contain proline, glycine and tyrosine residues (P^4^GYPPTGYPP^13^ and P^4^GYPPPPGGYPP^15^) were reported to bind to the penta-EF-hand domain of ALG-2 (apoptosis-linked gene 2) protein in a calcium-dependent manner [72]. Thus, it can be inferred from the presence of RGD/KGD motifs in certain plant annexins that protein-protein interactions may occur either with integrin-like proteins or C2 domain containing proteins. Certain plant annexins may also utilize proline-rich domain sequences to interact with other proteins during Ca^2+^-mediated signaling.

In conclusion, this study identified annexin superfamilies in 16 completely sequenced plant genomes. The comparative genome analysis of these sequences provided an insight into their origin, as well as their structural and phylogenetic relationships. Our analysis on the diversity of CBS sites and the occurence of K/H/RGD motifs indicated the complexity of plant annexin function(s). This study provides a basis for further systematic analysis of members of annexin multigene families in each of the plant lineages by using genetic (overexpression or gene knockouts) and biochemical approaches to determine their biological roles.

## Supporting Information

Figure S1 **The alignment with secondary structure, conservation and consensus sequence information used to build the phylogenetic trees (** **Figure 1A,B** **) and for sequence logos (** **Figure 2** **) from 149 identified annexin sequences.** Taxon-specific indels are removed to optimize the alignments. The numbers adjascent to amino acid sequence alignment does not represent the actual sequence lengths. The first line in each block shows conservation indices for positions with a conservation index above 5. The last two lines show consensus amino acid sequence (Consensus_aa) and consensus predicted secondary structures (Consensus_ss). Representative sequences were denoted by the abbreviated species names followed by locus names or the protein ID. Amino acids in the alignment are colored according to predicted secondary structures (red: alpha-helix, blue: beta-strand). Consensus predicted secondary structure symbols: alpha-helix: “h” and beta-strand: “e”. Conserved amino acids represented in bold and uppercase letters such as M, A, G, L, W, R etc., aliphatic (I, V, L): “l”, aromatic (Y, H, W, F): “@”, hydrophobic (W, F, Y, M, L, I, V, A, C, T, H): “h”, alcohol (S, T): “o”, polar residues (D, E, H, K, N, Q, R, S, T): “p”, tiny (A, G, C, S): “t”, small (A, G, C, S, V, N, D, T, P): “s”, bulky residues (E, F, I, K, L, M, Q, R, W, Y): “b”, positively charged (K, R, H): “+”, negatively charged (D, E): “−”, charged (D, E, K, R, H): “ c”.
(DOC)

Figure S2 A–D Alignments used to detect calcium binding sites and also to build the sequence logos for the four annexin domains. Taxon-specific indels are removed to optimize the alignments.
(DOC)

Table S1 **Summary of 149 annexin genes identified in Viridiplantae and their sequence features.**
(DOC)

Table S2 **Gene structure organizations in Spike moss, moss,** ***Arabidopsis*** **and rice showing the total number of exons.**
(DOC)
