# Supplementary material for: Genome-wide Comparative Analysis of Annexin Superfamily in Plants
Source: PLoS One. 2012 Nov 2;7(11):e47801. doi: 10.1371/journal.pone.0047801 (PMC3487801; doi:10.1371/journal.pone.0047801)
Supplement: Figure S1 — The alignment with secondary structure, conservation and consensus sequence information used to build the phylogenetic trees ( Figure 1A,B ) and for sequence logos ( Figure 2 ) from 149 identified annexin sequences. Taxon-specific indels are removed to optimize the alignments. The numbers adjascent to amino acid sequence alignment does not represent the actual sequence lengths. The first line in each block shows conservation indices for positions with a conservation index above 5. The last two lines show consensus amino acid sequence (Consensus_aa) and consensus predicted secondary structures (Consensus_ss). Representative sequences were denoted by the abbreviated species names followed by locus names or the protein ID. Amino acids in the alignment are colored according to predicted secondary structures (red: alpha-helix, blue: beta-strand). Consensus predicted secondary structure symbols: alpha-helix: “h” and beta-strand: “e”. Conserved amino acids represented in bold and uppercase letters such as M, A, G, L, W, R etc., aliphatic (I, V, L): “l”, aromatic (Y, H, W, F): “@”, hydrophobic (W, F, Y, M, L, I, V, A, C, T, H): “h”, alcohol (S, T): “o”, polar residues (D, E, H, K, N, Q, R, S, T): “p”, tiny (A, G, C, S): “t”, small (A, G, C, S, V, N, D, T, P): “s”, bulky residues (E, F, I, K, L, M, Q, R, W, Y): “b”, positively charged (K, R, H): “+”, negatively charged (D, E): “−”, charged (D, E, K, R, H): “ c”. (DOC) [file pone.0047801.s001.doc]

**Figure S1**

Conservation: 8 5 7 6 6 5 5

Bd4g31920 1 MASQ-------SPF-ENDCREIHGMCD------EPCRLSRLLAH--RSSSERQQIKVTY--RAMFG-EDL 51

Os09g27990 1 MASRCL---VTTGF-EDECREIHDACN------QPRRLSVLLAH--RSPSERQKIKATY--RTVFG-EDL 55

Zm02g31380 1 MASA-------AGS-EEACREIRRTCG------APRRLGLLLAP--RSPAERQQIRAAY--RARFG-EDL 51

Sb02g026390 1 MASAG--SSEEEEE-EEACREIRGASG------TPRRLGLLLAP--RSPAERQQIRAAY--RATFG-EDL 56

Cs340270 1 MATKIH--NMSIND-LGIENDCRDIHDS--WDQRSNVMVRVLAG--RNAMERQQTRRIY--KEIYG-EDL 60

Pt13g04990 1 MGTKIL-TSSSHGF-ENECKEIHDSWG------RLNHLVRSLAG--RSKLERQQIRETY--KAMYG-EDM 57

Cp00161g00040 1 MGTAVL-PSSSHGF-ENECREIHECWG------RTNRLIRALAN--RTRVERKQIRETY--KTMYG-EDL 57

Vv08g00710 1 ---MPW-PSSHGGL-YIYATALADQTE----G-HSSQLVQPLGG--RARLELGYIQEAF--MELVG-EVP 55

Gm13g26040 1 MANNIL-TMTNLNF-ELDCK---RTHDS--LGSLS-QLITSLAC--VTHHERQQLRETF--KAVNG-EDL 57

Os09g20330 1 MASIR---DFAKRY-EADCRHLNQFFSGNVSPNNARPVLEIFTA--RSSQEMKQICRAY--SSMYR-QDL 61

Cp00042g00810 ----------------------------------------------------------------------

Os09g23160 1 MASLTL-PPAPTNP-RQDAIDLHKAFKG--FGCDSTTVINILTH--RDSMQRALIQQEY--RTMYS-EDL 61

Bd4g29680 1 MASLTV-PPGPPNP-RQDAIELHKAFKG--FGCDSTAVTNILGH--RDSMQRGYIQHEY--KTMYS-EEL 61

Sb02g024090 1 MASLTL-PPAPPNP-RQDAIDLHKAFKG--FGCDSTAVINILTH--RDSVQRGLIQQEY--RAMYH-EEL 61

Zm02g30240 1 MASLTL-PPAPPNP-RQDAIDLHKAFKG--FGCDSTTVINILTH--RDSVQRGLIQQEY--RAMYH-EEL 61

Zm07g13390 1 MASLTL-PPAPPNP-RQDAIDLQKAFKG--FGCDSTTVINILTH--RDSVQRGLIQQEY--RAMYH-EEL 61

Bd3g36240 1 MASLSV-PPVLTSP-RQDAAALHKAFKG--FGCDSTTVINILAH--RNATQRALIMQEY--RAIYH-QDL 61

Zm04g13650 1 MASLTM-PPVPAWP-RQDAIDLHKAFRG--FGCDSTTVTNILAH--RDATQRSLIQQEY--RAVFN-QDL 61

Sb07g020760 1 MASLTV-PPVPTWP-RQDAIDLHKAFRG--FGCDSTTVISILAH--RDAAQRAAIAQEY--RAVFN-QDL 61

Os08g32970 1 MASLSV-PPVPTDP-RRDAIDLHRAFKG--FGCDATAVTAILAH--RDASQRALIRRHY--AAVYH-QDL 61

Cs234810 1 MC----------------------VYIG--FGCDNGAVVNILAH--RDAAQRSLIQREY--KAMYH-KDL 41

Cs234800 1 MSSLTI-PPLLTSP-RDDAALLYRAFKG--FGCDTAAVINVLAH--RDAAQRALIQQEY--RAIYS-EEL 61

Vv01g05380 1 MSSVTV-PPVLTSP-RDDAIQLYRAFKG--LGCDTAAVVHILAH--RDVTQRGLIQQEY--RAMYS-EDL 61

Gm08g06100 1 MATLNV-PPLPPSP-RDDAIQLYAAFKG--FGCDTSVVINILAH--RDATQRAYIQQEY--KAMYS-GDL 61

Mt8g107640 1 MSTLNV-PPIPPSP-RDDAMQLHRAFKG--FGCDTSAVINILAH--RDATQRAYLQQEY--RATYS-EDL 61

Gm07g12030 1 MATLTV-PPVPPSP-RDDAMQLYRAFKG--FGCDTSAVINILAH--RDATQRAYIQQEY--RSMYS-EEL 61

Gm09g30190 1 MATLTV-PPVPPSP-RDDAMQLYRAFKG--FGCDTSAVINILAH--RDATQRAYIQQEY--KAMYS-EEL 61

Pt15g04350 1 MATLSV-PPVLSSP-RDDAMQLFRAFKG--LGTDTSAVINILAH--RDAAQRSLIQHEY--RTLYS-EDL 61

Pt12g03690 1 MSTLIV-PPLLSSP-RDDAMHLYRAFKG--FGTDTSAVISILAH--RDAAQRALIQHEY--RALYA-EDL 61

Cp00003g03400 1 MSSLSV-PPFPTNP-KDDAIQLYRAFKG--LGCDTPVVINILAH--RDATQRALIQQEY--RLMYS-EDL 61

Gm07g28080 1 MTTLSV-PPVIPSP-REDAIKLHKAFKG--LGCDTSKVIKILAH--RNAEQRSLIQQEF--ETNYS-ELL 61

Gm20g01460 1 MATFHL------------PVSKHHAFSA-SLGCDTSKVIKILAH--RNAEQRSLIQQEF--ETNYS-ELL 52

Cp00002g01210 1 MATLKT-PANSPSP-RDDAMQLYQAFKG--RGCETSVIINILAH--RNATQRGLIEQEY--ETKYS-EEL 61

At1g68090 1 MATMKI-PMTVPSP-RVDADQLFKAFKG--RGCDTSVIINILAH--RNATQRALIEQEY--ETKFS-DDL 61

Pt10g10090 1 MSTLPK-PSMQTSS-RDDAVQLNRAFKG--LGCDTAVVVNVLGN--RNASQRDSIQQEY--ETLFS-DDL 61

Pt08g13700 1 MSTFTK-PSMQKSS-RDDAEQLNRAFKG--LGCDAAVVVNILAL--RNASQRDSIQQEY--ETLFS-DDL 61

Sm271856 1 MSTITV-PPMLPPV-QQDCQALHHAFKG--FGCDEKHVIQILAH--RNYLQRRELVNAY--RSMYG-EDL 61

Sm167346 1 MSTITV-PPMLPPV-QQDCQALHHAFKG--FGCDEKHVIQILAH--RNYLQRRELVNAY--RSMYG-EDL 61

Sm124402 1 MSTITV-PPMLPPV-QQDCQALHHAFKG--FGCNEKHVIQILAH--RNYLQRRELVNAY--RSMYG-EDL 61

Sm94768 1 MSTITL-PPMPPPV-QQDCQALHHAFKG--FGCDEKHVIQILAH--RNYLQRRELVNAY--RSMYG-EDL 61

Sm227533 1 MSTITV-PPMLPPV-QQDCQALHHAFKG--FGCNEKHVIQILAH--RNYLQRRELVNAY--RSMYG-EDL 61

Pp1s219_3V6 1 MATISL--PSYLNM-GEDVRELHRAFKG--FGCDEKKVIQILAH--RTQSQRLAIADAY--HHQYG-ESI 60

Pp1s37_276V6 1 MSTITV--PPYLSM-SDDVHALHRAFRG--FGCDEKRVIQILAH--RTQPQRDAIADAY--QRQYG-ESI 60

Pp1s6_292V6 1 MGTLTL--PPYFNL-QEDCKDLRSSFKG--LGCNEKRVIEILAR--RTQAQRLEIAQAY--QTVYG-ESL 60

Pp1s102_141V6 1 MGTLTL--PPCFNL-QEDCKELRSSLKG--LGSNEKKVIEILGR--RTQAQRLEIAQAY--QTVYG-ESL 60

Pp1s1_594V6 1 MGTLTL--PPYFNL-QEDCKELRLSFKG--LGCNEKRVIEILGR--RTQSQRLEIAQAY--QTVYG-ESL 60

Pp1s61_299V6 1 MGGVKQ-FQQYPGL-EDDIRDLRYALSG--LFPNERKVVEILGK--RSQAHRESIAEGY--KLLFA-ESL 61

Pp1s38_63V6 1 MGTTNV-QQSYPNL-HEDCKDLRNALRG--ISSNEKKVIEILGQ--RNQSQRDSLSEAY--KLVFG-EDL 61

PsABK21977 1 MAPFASTSCSSTQD-IKDCEAVYNCCKG--IAASKGRLEHILAS--RNATERKELGDLF--YALYK-EDL 62

PsACN40166 1 MAPFASTSCSSTQD-IKDCEAVYNCCKG--IAASKGRLEHILAS--RNATERKELGDLF--YALYK-EDL 62

Cp00213g00130 1 MATLQV-PANVPSP-AEDADQLHKAFQG--WGTNEGLIISILAH--RNAAQRNLIRQTY--AETYG-EDL 61

Bd3g58830 1 MATLSV-PAAVPPV-AEDCEQLRKAFQG--WGTNERLIISILAH--RDAAQRRAIRAAY--AEQYG-EEL 61

Os02g51750 1 MATLTV-PAAVPPV-AEDCEQLRKAFKG--WGTNEKLIISILAH--RDAAQRRAIRRAY--AEAYG-EEL 61

Sb04g027590 1 MATLTV-PSSVPAV-AEDAEQLHKAFEG--WGTNEKLIISILAH--RNAAQRRAIRRAY--AEAYG-KEL 61

Zm05g40790 1 MATLTV-PSSVPAV-AEDCEQLHKAFEG--WGTNEKLIISILAH--RNAAQRRAIRRGY--AEAYG-KEL 61

Bd1g45487 1 MATLTV-PSSVPAI-TDDCEQLRKAFQG--WGTNEALIISILGH--RDAAQRRAIRRAY--AETYG-EEL 61

Os06g11800 1 MATLTV-PSAVPPV-ADDCDQLRKAFQG--WGTNEALIISILAH--RDAAQRRAIRRAY--ADTYG-EEL 61

Zm06g16450 1 MATLKV-PATVPPV-ADDCDQLRKAFQG--WGTNEALIISILGH--RDAAQRRAIRRAY--AEAYG-EEL 61

Sb10g007760 1 MATLKV-PATVPPV-ADDCEQLRKAFQG--WGTNEALIISILGH--RDAAQRRAIRRAY--AEAHG-EEL 61

Cs273000 1 MSSIKA-PDHLPSP-AEDCEQLRKAFQG--WGTNEDLIISILAH--RNAAQRSLIRKAY--AETYG-EDL 61

At5g65020 1 MASLKV-PSNVPLP-EDDAEQLHKAFSG--WGTNEKLIISILAH--RNAAQRSLIRSVY--AATYN-EDL 61

Gm13g01870 1 MATLKV-PQPLPPV-ADDCEQLRKAFSG--WGTNEELIVSILAH--RNAAQRKLIRETY--AQTYG-EDL 61

Mt5g063670 1 MSTLSV-PHPLPPV-SDDVEQLRKAFSG--WGTNENLIISILGH--RNEVQRKVIREAY--AKTYE-EDL 61

Vv18g03470 1 MATLSV---------TEDCEQLRKAFAG--WGTNEGLIISILAH--RNAAQIKSIRQTY--AQTYG-EDL 54

Pt02g09420 1 MSTLTV-PQQVPPV-SEDVEQLRKAFSG--WGTNEGLIISILGH--RNAAQRKLIRQAY--AEAYG-EDL 61

Pt07g05300 1 MASLKV-PASVPPP-YEDAEQLHKAFEG--WGTNEGLIISILAH--RNAAQRNLIRKVY--AEAYG-QDL 61

Pt05g07550 1 MASLKV-PASVPPP-YEDAEQLNKAFKG--WGTNEGLIMSILAH--RNAAQRNLIRQVY--AEAYG-QDL 61

Gm05g31250 1 MATLKV-PAQLPSP-VEDSEQLRKAFQG--WGTNEGLIISILGH--RNAAQRKLIREAY--SATHG-EDL 61

Gm08g14460 1 MATLKV-PAQLPSP-LEDSEQLRKAFQG--WGTNEGLIISILGH--RNAAQRKLIREAY--STTHG-EDL 61

At1g35720 1 MATLKV-SDSVPAP-SDDAEQLRTAFEG--WGTNEDLIISILAH--RSAEQRKVIRQAY--HETYG-EDL 61

At5g10230 1 MASLKV-PATVPLP-EEDAEQLYKAFKG--WGTNERMIISILAH--RNATQRSFIRAVY--AANYN-KDL 61

At5g10220 1 MASLKI-PANIPLP-EEDSEQLHKAFKG--WGTNEGMIISILAH--RNATQRSFIRAVY--AANYN-KDL 61

Cp00036g01250 1 MSTLRV-PESVPSV-TEDCEQLNKAFSG--WGTNEGPIISILAH--INPNQCKLICQTY--AETYG-EDL 61

Cs217870 1 MATLSV-PDQLPPV-AEDCDRLHSAFQG--WGTDEGAIVSILAH--RNAKQRSLIRQTY--AETYG-EDL 61

Cp00042g00660 ----------------------------------------------------------------------

PsABK22223 1 MSTIIV-PTPTPTP-VEDSESLRKAFEG--WGTNEKLIIEILGH--RTAAQRRAIRQAY--TQLYE-EDF 61

Zm03g04200 1 MATIAV-PRVVPSP-AEDAAALLKAFQG--WGTDEQAVIGILAH--RDATQREQIALEY--EHKYG-ESL 61

Bd2g13620 1 MATITV-PQVIPSP-TEDADALMKAFQG--WGTDEQAVISILAY--RDAEQRKQIRLAY--QEKYD-ESL 61

Os01g31270 1 MATIVV-PPVTPSP-AEDADALLKAFQG--WGTDEQAVIGVLAH--RDATQRKQIRLTY--EENYN-ENL 61

Zm08g03950 1 MATITL-PRVVPSP-AEDAAALLKAFQG--WGTDEQAVISILAH--RDATQRKQIALEY--EHEYS-ESL 61

Sb03g004990 1 MATITV-PRVVPSP-AEDAAALLKAFQG--WGTDEQAVISILAH--RDATQRKQIALEY--EHKYS-ESL 61

Gm13g26960 1 MATLV--APNQKSP-VEDVEALHKAFKG--WGTDEKTVIAILGH--RNVHQRQQIRKVY--EEIYQ-EDL 60

Gm15g38010 1 MATLV--APNQKSP-VEDVEALHKAFKG--WGTDEKTVIAILGH--RNVHQRQQIRKIY--EEIYQ-EDL 60

Cs308090 1 MATLI--VPRDVPSANVDAEALRTAFKG--WGSDEKAIISILAH--RNAIQRRHIRIAY--EQLFQ-EDL 61

Cs308080 1 MATLL--VPHDVPPPNVDAEAIKAAFRG--WGTDEKAIVAVLGY--RNAPQRRQIRIAY--EQLFE-EDL 61

Gm13g26990 1 MATLI--APSNHSP-QEDAEALRKAFEG--WGTDENTVIVILGH--RTVYQRQQIRRVY--EEIYQ-EDL 60

Gm15g38040 1 MATLI--APSNHSP-QEDAEALRKAFEG--WGTDEKTVIVILGH--RTVYQRQQIRRVY--EEIFQ-EDL 60

Mt8g038210 1 MATLV--VQDLKVP-NEDAEALRKAFEG--WGTDEKTVITILGH--RNSNQIQQIRKAY--EGIYN-EDL 60

Mt8g038220 1 MATLI--APINHSP-VADAEALHGAFKG--WGTDEKSVITILGH--RNVYQRQQIRKSY--QEIYQ-EDI 60

Gm11g21480 1 MATLI--APSHHSR-VEDAEALRNAFKG--WGADDKAIIAILGH--RNVHQRQEIRKAY--EEIYQ-EDL 60

Mt3g018780 1 MATIV--VHSQTSP-VQDAEALRLAFKG--WGADNKAIIAILGH--RNVHQRQQIRKAY--EELFE-EDL 60

Gm04g27100 1 MS----------------------------WGADGKAIIAILGH--RNATQRTLIREAY--QNLFQ-EDL 37

Mt8g038180 1 MATLI--APMNHSP-KEDADVLWKAVKG--WGTDESAIIAIMGQ--RNAVQRQQIRQAY--QDIYQ-EDL 60

Cp04842g00010 1 MASLV--VPSQVSV-VEDAEALNKAFKG--WGTDEKTVIVILGH--RNAAQRKQIRMAY--EEIYQ-EDL 60

Cp36671g00010 1 MASII--VPSHVSV-VEDAEALRKAVQG--WGTDEKAIILLLGH--RNSAQRKQIRIAY--EEIYQ-EDL 60

Pt03g19020 1 MATLV--VPENVSY-ADDAQALRKACQG--WGTNEKAIISILGH--RNAAQRKQIRLAY--SELFQ-EDL 60

Mt3g018920 1 MT-------------------------------------------------------------------- 2

Mt3g018790 1 MASLI--APSNHSP-VEDAEALQRAVKG--WGADEKAIIAILGH--RNGTQRTQIRQAY--YELYQ-EDL 60

Cp00157g00670 1 MASLV--APKQFSP-VEDAENIKKACLG--WGTDERAIISILGH--RNVFQRKLLRLAY--QEIYQ-EDL 60

Cs138380 1 MATLI--TPKYFSP-VEDAENIKKACLG--LGTDENAIISILGH--RNATQRKLIRLAY--EEIYN-EDL 60

Pt01g27650 1 MATVV--APKDFSP-VEDAETIKKACLG--LGTDEKAIISVLGN--RNSFQRKLIRLAY--EEIYH-EDL 60

At5g12380 1 MATIV--SPPHFSP-VEDAENIKAACQG--WGTNENAIISILGH--RNLFQRKLIRQAY--QEIYH-EDL 60

Vv06g10680 1 MATLV--APEDFSP-GEDALAINRACQG--WGTDEKAIISILGH--RNAAQRKQIRLAY--QEIYL-EDL 60

Cp00197g00010 1 MASLI--VHAQASA-VEDAEALRKAFAG--LGTNEKAIISILGH--RNAAQRKQIRVEY--ELLYK-EDF 60

Gm11g21460 1 MATLI--APSNHPP-VEDTESLRKAVKA--FSHK--NRVQLVLP--KDSNQRSTYSRIS--AKRIS-SNA 58

Gm13g27000 1 MATLV--APRNHFP-QEDAEALWKAVKG--WGTDEKTIIKILGH--RNASQRQQIRLVF--QDIHL-EDL 60

Gm15g14350 1 MATLI--AAKHSSS-IEDAENIKKACKG--LGTDETALISILAH--RNVAQRKLVRMAY--EELYQ-EDL 60

Os05g31750 1 ----------MADE-IQHLTRAFSGLGG--LGVDEPAMVSALAKWRRQPEKLSGFRKSF--NGFFK-DHE 54

Bd2g26760 1 ----------MADE-VQALTKAFSGLGG--LGVDETTMVSTLAQWRKQPEKRSGFRKSF--RGLFK-EHE 54

Zm06g23270 1 ----------MADE-VQQLTRAFSGLGG--LGVDEPAMVSALARWRGQPEKRSAFRKGF--PGFFS-SHE 54

Bd1g62130 1 ----------MADE-QQELTRAFSGLGG--LGVEETALVSALGRWRKQPEKRASFRRGF--PGFFS-PAE 54

Zm01g15800 1 KEAPPP----MADE-HQDLTRAFAGLGG--LGVDETALVSALGRWRREPEKRAQFRRGF--PGFFS-SSE 60

Sb01g035050 1 KEAPPA---AMADE-HQDLTRAFAGLGG--LGVDETALVSVLGRWRRQPEKRAQFRRGF--LGFFSASAG 62

Mt8g038170 1 MAF------------NQELEAITQAFSG--HGVDEKSLIAVLGK--WDPLERETYRKKT--SHFFI-EDH 51

Cs308100 1 MA-------------DSAVEVLTRALSG--HGINENAMIETLGK--WDHEEKKLFRKKS--SHFFS-EDH 50

Gm13g27010 1 MAF------------NQELEAVTQAFSG--HGVDEKSLVTLLGK--WDPLERESFRKKT--PHLFS-EDQ 51

Gm15g38060 1 MAF------------NQELEAVTQAFSG--HGVDEKSLVTLLGK--WDPLERESFRKKT--PHLFS-EDQ 51

Pt01g06030_ 1 MA---------------NLEALAKAFTG--LGVDEKSLIENLGK--SHPEQRTLFRKKT--PQLFI-EDH 48

Vv00g25070 1 MAL------------SHEFQALTKSFSG--FGVDEKSMISILGK--WHQDDRKSYRKGC--PQFFT-QDR 51

At2g38750 1 MA--------LPLE-LESLTEAISAGMG--MGVDENALISTLGK--SQKEHRKLFRKAS--KSFFV-EDE 54

Zm08g13570 1 MASISV-PDPVPSA-TEDAENIRKAAVQ-GWGPDKKALMEILGH--RTAAQRAEIAAAY--AGRYN-ESL 62

Bd2g26770 1 MASISV-PDPVPAP-TEDAENIRKAVEG--WGTDEKALIEILGH--RTAAQRAEIAVAY--EGLYD-QPL 61

Zm06g23280 1 MASISV-PSRAPSA-AEDAENIRKAVQG--WGTDEKALIEILGH--RTAAQRAEIAVAY--EGLYN-EPI 61

Sb09g018980 1 MASISV-PNPVPSA-TQDAENIRKAVQG--WGTDEKALIEILGH--RTAAQRAEIAVAY--EGLCN-ESL 61

Os05g31760 1 MASISV-PNPAPSP-TEDAESIRKAVQG--WGTDENALIEILGH--RTAAQRAEIAVAY--EGLYD-ETL 61

Sb02g041850 1 MSTVAV-PSPPPTA-SEDAESLRTALQ--------AALIGLNCF--RYKLSRHCI--------------- 43

Bd1g62120 1 MSTITV-PTPLPSP-AADAESLWNAVQ------------------------------------------- 25

Sb01g035040 ----------------------------------------------------------------------

Bd1g18990 1 MSTIAV-PSPVPSP-AEDAEGIWKALQ----G-----KYACNCI--VFSFQLYHI--------------- 42

Os07g46550 1 MSINAV-PSPVPSA-SDDAESLRKALQ---------VRHGLNCF--RYKLSRHCL--------------- 42

Pt01g06020 1 MATLRV-PEVVPSP-TQDCEKLRDAVQG--LGTDEKAIIWILGH--RNASQRKKIRETY--QQLYN-ESL 61

Gm15g38070 1 MASLKL-PEVVPSP-TQDSERLRKAFQG--FGTDEKAVILVLGH--RNAQQRKKIGETY--QQLYN-ESL 61

Gm13g27020 1 MASLKL-PEVVPSP-TQDSERLRKAFQG--YGTDEKAVILVLGH--RNAQQRKEIRETY--QQLYN-ESL 61

Mt8g038150 1 MASLKL-PEIVPSP-NTDTERLRNAFQG--IGTNEKELILVLGH--RNAQQRREIRETY--QKLYN-ESL 61

Vv00g25060 1 MSTLRV-PDAVPPP-AQDCEKLQKAFQG--WGTDEKAIIWVLGH--RNASQRRIIRDTY--QHLYN-ESL 61

Cp00197g00020 1 MSTIRV-SDTVPSP-TEDSHTLKEAFQG--FGTDEKAIIKVLGA--RTARQRREIRDTY--QQLYN-ENL 61

At2g38760 1 MATIRV-PNEVPSP-AQDSETLKQAIRG--WGTDEKAIIRVLGQ--RDQSQRRKIRESF--REIYG-KDL 61

Vv00g00800 1 MASLRP-PDSIPSP-AQDSERLSLALQG--RGVDEKVIVWILGH--RNAIQRKRIKDTY--QQLYK-ESI 61

Vv03g02080 1 MASLRL-PDSIPSP-AQDSERLNLALQG--RGVDEQVIVWILGH--RNAIQRKQIKDTY--QQLYK-ESI 61

Vv00g00650 1 MASLRL-PDSIPSP-VQDSERLNQALQG--RGVDEKVIVWILGH--RNAIQRKQIKDTY--QQLYK-ESI 61

Vv00g00720 1 M--------------------------------------------------------------------- 1

Vv00g00760 1 MC-------IIPGP-SNGSE--------------AGLLLFI----------------------------- 19

Vv00g00750 1 MATLRL-PDVVPSP-TQDSERLRVALQG--WGVDQEVIIWILGH--RKAVQRKKIKETY--QQLFK-ESI 61

Vv00g00710 1 MATLTL-PAVAPSP-TQDSERLRVALQG--WGVDQEVIIWILGH--RNAVQRKKIKETY--QQLFK-ESI 61

Vv00g00660 1 MATLRL-PDVAPSS-TQDSERLRVALQG--WGVDQEVIIWILGH--RNAVQRKKIKETY--QQLFK-ESI 61

Ot24272 1 KRVLLL-AGCDGVG-ESYAKVINSAVSG--LGTDTSAIIRLMVT--ATPEQLDATREAY--SRIYK-KDL 61

MRCC299_03g01870 1 LRDVLD-ALLTEQF-DFEAQVLHRAMKG--WGCDEDTLTTILCT--LDEADIFKLQNAY--SSRFE-KSL 61

Cs307980 1 -------------------MILPEAGLG--IGIDEKKLVDMVRR---SDFNPGNIKRRR--ELIMI---- 40

Cs307970 1 LFSFHCAKNHSPFL-RAAYDAFEQSLAG--VGINENGIVKTLTN--FDADEYRLMSSNFKYDQSLG-YIW 64

[Consensus_aa:](http://prodata.swmed.edu/promals3d/info/consensus.html) **M**to*h*...ss...s...pc*h*p.*l*.pt*h*p**G**..*h***G***h*sp..*ll*.*l***L**tp..ps..p**R**..*l*p..*@*....*h@*..cs*h*

[Consensus_ss:](http://prodata.swmed.edu/promals3d/info/consensus_ss.html) h hhhhhhhhhhhh hhhhhhhhh hhhhhhhhhhh hhhh h

Conservation: 7 768 65

Bd4g31920 52 V---GRLRNT--LLPDQD-----NELCNLL-YLWMLDP--AERDAIMARDAIESGLT---GY--RALVEI 103

Os09g27990 56 A---GEVQKI--LMVNQE-----DELCKLL-YLWVLDP--SERDAIMARDAVENGGA-TDYR---VLVEI 108

Zm02g31380 52 A---ATLHGT--LAAPNTN--QVDELSKLL-YLWALEP--AERDAVVAREAVEGGVT-AAGY--RALVEV 108

Sb02g026390 57 A---ATLHG------NQD-----DELCKLLYLWGALEP--AERDAVVAREAVEGGVT-VAGY--RALVEV 107

Cs340270 61 V---DRLGTI--DVEPIN-----RALSL-----WMLDS--HERDAVFAREALEPGDT-NFK----ALIEI 108

Pt13g04990 58 A---ILLQKM--QFSKVC-----AALYR-----WMMDT--YERDAIVAREAFGQGDV-NYK----ALVEI 105

Cp00161g00040 58 A---SFLFRR--SNAKAS-----AALLM-----WMLDS--CERDAVVAREALKQDDI-DFK----ALVEI 105

Vv08g00710 56 I---NRFQNG--YMITQR-----NEPGG-------LDP--HPSDAVVVREALEQGDT-NYK----VLVEI 101

Gm13g26040 58 I---SHLQRY--EDAFYSM--NCSAISL-----WMLDT--HDRDAVVAREALQQDET-NFK----ALVEI 108

Os09g20330 62 I---QLLSQQ--LSGSIA-----IRVACL----RASEP--CVRDADIARDALFGRRI-DGD----VLVEV 110

Cp00042g00810 1 -----MATNQ--GNSPFA-----RVAYL-----RMSKP--QERDAEIMRHSLFGGRI-NLH----NLIEV 46

Os09g23160 62 S---RRISSE--LSGHHK-----KAMLL-----WILDP--AGRDATVLREALSGDTI-DLR----AATEI 109

Bd4g29680 62 S---RRISSE--LSGNHK-----KAMSL-----WILDP--AGRDATVLREALSADSL-DLR----AATDI 109

Sb02g024090 62 F---HRISSE--LSGNHK-----KAMSL-----WILDP--AGRDATVLREALSGDTM-DLR----AATEI 109

Zm02g30240 62 S---HRISSE--LSGNHK-----KAMSL-----WILDP--AGRDATVLREALNGDTM-DLR----AATEI 109

Zm07g13390 62 S---HRISSE--LNGNHK-----KAMLL-----WILDP--AGRDATVLREALSVDTM-DLR----AATDI 109

Bd3g36240 62 Y---HRLSTE--LTGNHKN--SFKAMLL-----WILDP--AGRDATILNQALNSDIP-DLR----AATEI 112

Zm04g13650 62 A---RRIASE--LSGHHK-----RAMLL-----WILDP--ATRDATILKQALTGDIT-NLR----AATEI 109

Sb07g020760 62 A---RRLASE--LSGNHK-----RAMLL-----WVLDP--ATRDATVLKQALTGDVT-DLR----AATEV 109

Os08g32970 62 L---HRLAAE--LSGHHK-----RAVLL-----WVLDP--ASRDAAVLHQALNGDVT-DMR----AATEV 109

Cs234810 42 I---KHLKSE--LSGNLE-----KAILL-----WMYDP--GTRDAVIVKEALSGDTI-HLR----RATEV 89

Cs234800 62 T---KRLKSE--LSGKLE-----DAILL-----WMYDP--ATRDAILVKNAIYGETS-TLR----AATEV 109

Vv01g05380 62 V---KRLSSE--LSGNVK-----RAVLL-----WVQDP--AGRDASIVRQALSGNVV-DLK----AATEV 109

Gm08g06100 62 L---KRLSSE--LSGKLE-----TALLL-----WMHDP--AGRDAIILRQSLTLPK--NLE----AATQL 108

Mt8g107640 62 L---KRLSSE--LSGKFE-----NAILL-----WMHDP--ATRDAIILKQTLTVSK--NLE----ATTEV 108

Gm07g12030 62 S---KRLASE--LSGKLE-----TAVLL-----WLHDP--AGRDATIIRKSLTADNR-SIE----GATEV 109

Gm09g30190 62 S---KRLASE--LSGKLE-----TAVLL-----WLHDP--AGRDATIIRKSLTADNK-TLE----GATEV 109

Pt15g04350 62 F---KRLSSE--LTGNLE-----TAVLF-----WMHDL--PGRDAIIVRQALMMNTM-NLE----AATEV 109

Pt12g03690 62 L---KRLTSE--LTGKLE-----TAVLL-----WMHDL--PGRDAIIVRQALIADIL-NLE----TATEV 109

Cp00003g03400 62 L---KRLASE--LHGKLE-----RAVLL-----WILDP--AARDATAIRQAYVAA---DLR----ALTEI 107

Gm07g28080 62 S---KRLSKE--LRGHVK-----KAVLL-----WLHDP--ATRDAKVVRKALTISVV-DNQ----AITEI 109

Gm20g01460 53 S---KRLSKE--LRGHVK-----KAMLL-----WLHDP--ATRDAKVVRKALTASVV-DNQ----ALTEI 100

Cp00002g01210 62 S---KRLYSE--LHGHLK-----KAVVL-----WMNDP--VTRDAKILAQALRGSLT-DHK----AVTEV 109

At1g68090 62 R---KRLHSE--LHGHLK-----KAVLL-----WMPEA--VERDASILKRSLRGAVT-DHK----AIAEI 109

Pt10g10090 62 K---KQLALE--LHGHLK-----KAVLL-----WMKSP--VERDVTTLRQALTGPII-DIK----TATEI 109

Pt08g13700 62 K---KQLAHE--LHGHLK-----KAVLL-----WMKSP--IERDVTTLRQALTGPLF-DVK----AATEI 109

Sm271856 62 L---RRLEKE--LHGNLE-----QAVLL-----WMMEP--AERDAVLIRDAMKGLGT-KDK----TLIEI 109

Sm167346 62 L---RRLEKE--LHGNLE-----QAVLL-----WMMEP--AERDAVLIRDAMKGLGT-KDK----TLIEI 109

Sm124402 62 L---RRLEKE--LHGKLE-----QAVLL-----WMMEP--AERDAVLLRDAMKGLGT-KDK----TLIEI 109

Sm94768 62 L---RRLEKE--LHGNLE-----QAVLL-----WMLEP--AERDAVLIRDAMKGLGT-KDK----TLIEI 109

Sm227533 62 L---RRLEKE--LHGKLE-----QAVLL-----WMMEP--AERDAVLLRDAMKGLGT-KDK----TLIEI 109

Pp1s219_3V6 61 H---KRLKSE--LHGKLE-----EVMLL-----WMMGP--AQRDAILIYDSMKGLGT-KDS----ALIGI 108

Pp1s37_276V6 61 H---KRLKSE--LHGKLE-----KAVLL-----WMMTP--AQRDATLVNESMNGLGT-TDH----ALVGI 108

Pp1s6_292V6 61 H---KRLKSA--FSGKLE-----KCILL-----WMMDS--AERDAILLYELMKVGGR-KADR---AFIGI 109

Pp1s102_141V6 61 H---KRLKSA--FSGKLE-----KCILL-----WMMDS--AERDAILMHELMKVGGK-ADR----SLIGL 108

Pp1s1_594V6 61 H---KRLKAA--FNGKLE-----KCILL-----WMMDS--AERDAILMYELMKIGGR-KADR---ALIGI 109

Pp1s61_299V6 62 P---KRLKAS--MSCKAE-----RCLML-----WMMDP--SERDAVLLYEALSQGGP-KKDR---AVIGM 110

Pp1s38_63V6 62 R---KRLKSS--ISGKLE-----KCLTL-----WMMDP--FDRDAVLLNEALREGGP-KKDR---VIIGM 110

PsABK21977 63 S---TLLHAE--LWGNLE-----KAVVL-----WMHDP--AERDAIIAKTELRSQYP-DFR----ALTEI 110

PsACN40166 63 S---TLLHAE--LWGNLE-----KAVVL-----WMHDP--AERDAIIAKTELRSQYP-DFR----ALTEI 110

Cp00213g00130 62 L---KALDKE--LSSDFE-----RAVLL-----WTLDP--PERDAYLANESTKRFTS-SNW----VLLEI 109

Bd3g58830 62 L---RALGDE--IHGKFE-----RAVIQ-----WTLDP--AERDAVLASEEARKWHP-GGR----ALVEI 109

Os02g51750 62 L---RALNDE--IHGKFE-----RAVIQ-----WTLDP--AERDAVLANEEARKWHP-GGR----ALVEI 109

Sb04g027590 62 L---RALGDE--IHGKFE-----RTVIL-----WTLDP--AERDAVLANEEAKKWHP-GGR----ALVEI 109

Zm05g40790 62 L---RALGDE--IHGKFE-----RAVIL-----WTLDP--AERDAVLANEEAKKSHP-GGR----ALVEI 109

Bd1g45487 62 L---RSITDE--ISGDFE-----RAVIL-----WTLDP--AERDAVLANEGAKKWHP-GSP----VLVEI 109

Os06g11800 62 L---RSITDE--ISGDFE-----RAVIL-----WTLDP--AERDAVLANEVARKWYP-GSGS--RVLVEI 111

Zm06g16450 62 L---RSITDE--ISGDFE-----RAVIL-----WTLDP--AERDAVLANEAARKWKP-GNR----VLVEI 109

Sb10g007760 62 L---RSITDE--ISGDFE-----RAVIL-----WTLDP--AERDAVLANEAARKWQP-GNR----VLVEI 109

Cs273000 62 L---KALDKE--LSSDFEARLCCRIVLL-----WTLEP--ADRDAFMVNEATKRLTS-NNL----VIVEV 114

At5g65020 62 L---KALDKE--LSSDFE-----RAVML-----WTLDP--PERDAYLAKESTKMFTK-NNW----VLVEI 109

Gm13g01870 62 L---KALDKE--LTSDFE-----RLVHL-----WTLDS--AERDAFLANEATKKWTS-SNQ----VLVEI 109

Mt5g063670 62 I---KALNKE--LTSDFE-----RLVHL-----WTLES--AERDAFLANEATKRWTS-SNQ----VLVEL 109

Vv18g03470 55 L---KDLNKE--LSNDFE-----RVVLL-----WTLDP--AERDAFLANE-------------------- 87

Pt02g09420 62 L---KALDKE--LSNDFE-----RVLLL-----WTLDP--AERDAALANEATKRWTS-SNQ----VLMEI 109

Pt07g05300 62 L---KDLDKE--LSSDFE-----RAVLL-----WTLDP--AERDAYLANEATKRFTS-SNW----VLMEI 109

Pt05g07550 62 L---KDLDKE--LSSDFE-----RVVLL-----WTLDL--AERDAYLANEATKRFTS-SNW----VLMEI 109

Gm05g31250 62 F---KDLDKE--LSSDFE-----RAVLV-----WTLDP--AERDAFLANEATKMLTS-NNW----VILEI 109

Gm08g14460 62 L---KDLDKE--LSSDFE-----RAVMV-----WTLDP--SERDAFLANEATKMLTS-NNW----VILEI 109

At1g35720 62 L---KTLDKE--LSNDFE-----RAILL-----WTLEP--GERDALLANEATKRWTS-SNQ----VLMEV 109

At5g10230 62 L---KELDRE--LSGDFE-----RAVML-----WTFEP--AERDAYLAKESTKMFTK-NNW----VLVEI 109

At5g10220 62 L---KELDGE--LSGDFE-----RVVML-----WTLDP--TERDAYLANESTKLFTK-NIW----VLVEI 109

Cp00036g01250 62 L---KALDKE--LTNDFE-----RLVLL-----WTLRP--AERDAFLANEARK----------------- 97

Cs217870 62 L---KALDKE--LSSDFE-----RAVLL-----WTFHP--AERDALLANEAIRKL---KHF----VVLEI 107

Cp00042g00660 1 -----------------------------------MDP--AECDALLANESLRKR---AAGH--HVIMEI 28

PsABK22223 62 L---KRLQSE--LTREFE-----RALFL-----WSLDP--PERDALLAHESIKKWSP-KNR----SLIEI 109

Zm03g04200 62 V---QRLQSE--LTGDFE-----RAVYH-----WMLGP--AERQAVMANAATECLQE-ECA----VIVEI 109

Bd2g13620 62 L---QRLQSE--LTGDFQ-----TAMCH-----WVLDP--VERQAAMANAATKCIHE-EYP----VIVEI 109

Os01g31270 62 I---QRLQSE--LSGDLE-----RAMYH-----WVLDP--VERQAVMVNTATKCIHE-DYA----VIVEI 109

Zm08g03950 62 I---QRLQSE--LTGDLE-----RAVYH-----WMLGP--AERQAAMAHAATECVQE-RYA----VVVEI 109

Sb03g004990 62 I---QRLHSE--LSGDFE-----RAVYH-----WMLDP--AERQAVMANAATECIQE-EYP----VLVEI 109

Gm13g26960 61 I---KRLESE--LSGDFE-----RAVYR-----WMLEP--ADRDAVLANVAIKNGSK-GYH----VIVEI 108

Gm15g38010 61 I---KRLESE--LSGDFE-----RAVYR-----WMLEP--ADRDAVLANVAIKNGSK-GYH----VIVEI 108

Cs308090 62 I---KRLESE--ISGHFE-----RAVYR-----WMLDP--EDRDAVLANIAIRKPKE-DFA----VLVEL 109

Cs308080 62 V---KRFESE--LSGHLE-----RAVYR-----WILDP--EDRDAVLAHVALRKPNE-DFA----VLVEF 109

Gm13g26990 61 V---KRLESE--IKGDFE-----KAVYR-----WILEP--ADRDAVLANVAIKSGK--NYN----VIVEI 107

Gm15g38040 61 V---KRLESE--IKGDFE-----KAVYR-----WILEP--ADRDAVLANVAIKNGK--NYN----VIVEI 107

Mt8g038210 61 I---KRLESE--IKGDFE-----KAVYR-----WILEP--AERDAVLANVAIKSGK--NYN----VIVEI 107

Mt8g038220 61 L---KRLESE--LSGDFE-----RAVYR-----WMLEP--ADRDAVLANVAIKDGSK-SYH----VIIEI 108

Gm11g21480 61 I---KRLESE--ISGDFE-----RAMYR-----WMLQP--ADRDAVLVNVAIKNGTK-DYH----VIAEI 108

Mt3g018780 61 I---KRLESE--ISGDFE-----RAVYR-----WMLDP--ADRDAVLINVAIRNGNK-DYH----VVAEI 108

Gm04g27100 38 I---KRLESE--LSGDFE-----RAMYR-----WILEP--AEREALLANIAIKSADK-NYQ----VIVEI 85

Mt8g038180 61 I---KRLESE--LSGNFE-----KAMYR-----WILDP--ADRYAVLANVAIKSINK-DYH----VIVEI 108

Cp04842g00010 61 I---KRLESE--ISGDFE---------------------------------------------------- 73

Cp36671g00010 61 V---KRLESE--LSGDFE-----RAIYR-----WVLEP--ADRDAVLCHVAIRKHEP-DYH----VLVEI 108

Pt03g19020 61 V---KRLESE--LNGDFE-----KAVYR-----WVLDP--EDRDAVLANVAIRKSG--DYH----VIVEI 107

Mt3g018920 3 ------------YAFDVV-----RAMYR-----WILEP--AEREA------------------------- 23

Mt3g018790 61 I---KRLESE--LSGDFE-----RAMYR-----WILEP--AEREALLANIALRNANI-NYH----LIVEI 108

Cp00157g00670 61 I---QQLKSE--LSGDFE-----RAICH-----WTLDP--ADRDAVLANLALQKPVP-DYK----VIVEI 108

Cs138380 61 I---QQLNSE--LCGDFE-----RAICH-----WTLDP--ADRDATLANKALKSSTL-DYR----VIIEI 108

Pt01g27650 61 I---HQLKSE--ISGDFE-----RAMSQ-----WTLEP--ADRDAVLANAALQKSKP-DYR----VIVEI 108

At5g12380 61 I---HQLKSE--LSGNFE-----RAICL-----WVLDP--PERDALLANLALQKPIP-DYK----VLVEI 108

Vv06g10680 61 T---KQLKSE--LSGDLE-----RAICH-----WILDP--VERDAVLANEALKKARP-DYR----VILET 108

Cp00197g00010 61 L---SRLESE--LTRDFK-----RAVYL-----WMLEP--ADRDAVFVHEAIKKHKI-EYQ----VLIEI 108

Gm11g21460 59 V---TLREFF--FFFSID-----KSMYR-----WILEHVHVEREALLANIALKSADK-NYQ----VIVEI 108

Gm13g27000 61 V---KRLESE--LSGDFE-----RAVYR-----WTLEP--SKRYAVLANVAIKNANK-DYH----VMVEI 108

Gm15g14350 61 I---QQFKSE--LSGSFE-----RAICN-----WTMDP--AERDAAFINEALKKETP-DYK----VIVEI 108

Os05g31750 55 Y--MLHLAAE---FSRFK-----NLMVM-----WAMHP--WERDARLAHHVLHQAH--PAA----IVVEI 101

Bd2g26760 55 Y--MLHLAAE---FSRFK-----NLMVL-----WAMHP--WERDARLAHHVLHQAH--PPA----IAVEI 101

Zm06g23270 55 Y--MLHLAAE---FARFR-----DLVVL-----WATHP--WERDARLAHHVLHHHHH-HPPA---VVVEV 103

Bd1g62130 55 Y--VRHLKTE---FSRFK-----NLMVL-----WAMHP--WERDARWAHRALHKHK--KHQGSGCILVEL 105

Zm01g15800 61 Y--LLHLKAE---FARFK-----DAAVL-----WAMHP--WERDARWAHHVLHKAH--PPH----ILVEV 107

Sb01g035050 63 EY-LLHLKAE---FARFK-----DAAVL-----WAMHP--WERDARWAHHVLHKAH--PPQ----VLVEV 110

Mt8g038170 52 C---VRLLKH--EFVRFK-----NAVVL-----WSMHP--WERDARLAKEALKKGSI-SYG----VLIEI 99

Cs308100 51 G---MRLLKH--EFMRFK-----NAVVL-----WTTHP--WERDARLVKEALSKGHH-GQNI--NILIEV 100

Gm13g27010 52 Y---VRLLKH--EFVRFK-----NAVVL-----WTMHP--WERDARLVKEALKKGPN-EYG----VLIEV 99

Gm15g38060 52 Y---VRLLKH--EFVRFK-----NAVVL-----WSMHP--WERDARLVKEALKKGPN-AYG----VLIEV 99

Pt01g06030_ 49 C---VRLLKH--EFVRFK-----NALVL-----WAMHP--WERDARLVKEALKKGPQ-SYG----VIVEI 96

Vv00g25070 52 H---VAFLKH--EFLRLK-----NAVVL-----WTMHP--WERDARLMKEALVKGPQ-AYA----VIIEV 99

At2g38750 55 ERAFEKCHDH--FVRRFN-----TAVVM-----WAMHP--WERDARLVKKALKKGEE-AYN----LIVEV 105

Zm08g13570 63 L---DRLHSV--LSGDFR-----SAMML-----WTADP--AARDAKLAHKAMKKKGE-RYVW---VLIEV 111

Bd2g26770 62 I---GRLQDE--LSSHFR-----GAMML-----WTMDP--AARDAKLAYKALRKKGG-DRHA--WVLIEV 111

Zm06g23280 62 I---DRLHSE--LSGDFRD--SMSAMML-----WTVDP--AARDAKLAHKAMKKQGE-RYVW---VLIEV 113

Sb09g018980 62 L---DRLHSE--LSGDFR-----SAMML-----WTADP--AARDAKLAHKAMKKKGE-RYVW---VLIEV 110

Os05g31760 62 L---DRLHSE--LSGDFR-----SALML-----WTMDP--AARDAKLANEALKKKKK-GELRHIWVLVEV 113

Sb02g041850 44 ------------LSVDFW-----KAMIL-----WTMDP--AERDANLVHEAVKKK-K-KDESYVSVLVEV 87

Bd1g62120 ----------------------------------------------------------------------

Sb01g035040 1 -------------------------MVL-----WTIDP--AERDARLANQALGDRRM-MDDQHAWVLVEV 37

Bd1g18990 43 ------------LTDM-------KAMIL-----WTMDP--AERDANLLHGAIRLRGDGGENDHVFVLVEI 86

Os07g46550 43 ------------LSLDFW-----KAMIL-----WTMDP--AERDANLVHEALKKKQR-DETYYMSVLIE- 86

Pt01g06020 62 I---DRLNSE--LSGDFR-----KAVIL-----WTTDP--PERDAKLANEALKANKK-GMKQL-QVVVEI 112

Gm15g38070 62 V---DRLHSE--LSGDFR-----NAVIL-----WTYDP--PERHARLAKDALKAKKG-IKHL--QVLVEI 111

Gm13g27020 62 I---DRLNSE--LSGDFR-----NAVIL-----WSYDP--PERHAGLAKDALKAKKK-GTKHL-QVLVEI 112

Mt8g038150 62 L---DRLQSE--LSGDFR-----NAIVL-----WTCDP--PERDAKFARDALKVKRK-GIKQL-QILVEI 112

Vv00g25060 62 I---DRLQSE--LSGDFR-----NAVVL-----WTYDP--PERDARLAKEALKARKK-GINHL-QVIVEI 112

Cp00197g00020 62 I---DALFSE--LSGDFR-----KAVIL-----WTIDP--PERDAKLANEALQKKKI-GIKNL-KVIVEI 112

At2g38760 62 I---DVLSSE--LSGDFM-----KAVVS-----WTYDP--AERDARLVNKILNKEKKKKSLENLKVIVEI 114

Vv00g00800 62 I---HRLQSK--LSGVLK-----KAMSY-----WMEEP--PERDAKLVEKTLKRGKA-GITQL-QVIVEI 112

Vv03g02080 62 I---HRLQSK--LSSGLK-----TAMIL-----WMNEA--PERDAILANKALKRKRK-KINQL-QVLVEI 112

Vv00g00650 62 I---HRLQSK--LFGVFK-----TAMIL-----WMNEA--PERDAILANMALKRKRK-KINQL-QVLVEI 112

Vv00g00720 ----------------------------------------------------------------------

Vv00g00760 ----------------------------------------------------------------------

Vv00g00750 62 I---HCLQST--LSGVLG-----KAMSY-----WMEEP--PERDAKLVEKTLKRGKA-GITQL-QVIVEI 112

Vv00g00710 62 I---HCLQST--LSGVLG-----KAMSY-----WMEEP--PERDAKLVEKTLKRGKA-GITQL-QVIVEI 112

Vv00g00660 62 I---HCLQSA--LSGVLG-----KAMTY-----WMEEP--PERDAKLVEKTLKKGKA-GITQL-QVIVEI 112

Ot24272 62 I---KAVGSEWKVSGDFK-----RIITA-----LAKRD--YDFEIQSMRNAVEGLGT-DEQ----LIISI 111

MRCC299_03g01870 62 E---QAMLSE--TEGKYK-----RVLLLA----GCDGI--AEAYAKVCRSAIEGAGT-DCK----ALIRL 110

Cs307980 41 ---------E---FQRFM-----NATLM-----WMTSP--AERDARLLRKAIKTRGT-HVGI--MVIIEI 83

Cs307970 65 K---EKKRQS--MKLEFQR--IMNVTML-----WMTTP--IERDARLLRSALKMGDA-AGVS---VLIEI 116

[Consensus_aa:](http://prodata.swmed.edu/promals3d/info/consensus.html) *h*...p.*l*.pp..*h*.sp*h*c.....p*hhh*......**W***hh*c**P**..*h*c**RDA**.*lh*ppt*h*p............*hlh***E***l*

[Consensus_ss:](http://prodata.swmed.edu/promals3d/info/consensus_ss.html) h hhhhhh h hhh hhhhh hh h hhhhhhhhhhhh hh hhhhh

Conservation: 5 6 9 5 55 5 7 5

Bd4g31920 104 FTR-RKQEQLFFTKQAYLGRFKKNMEQD-MVTEPS-HPSRPYQR---LLVALAASH--KSH-HDE--PSW 162

Os09g27990 109 FTR-RKQNQLFFTNQAYLARFKKNLEQD-MVTEP----SHPYQR---LLVALATSH--KSH-HDE--LSR 164

Zm02g31380 109 FTR-RKQDQLFFTKQAYAVRFRRSLDQD-MATEP----SHPYHR---LLLALAASR--RSH-HDD--LSQ 164

Sb02g026390 108 FTR-RKQDQLFFTKQAYMARFRRNLDQDIMVTEP----SHPYQR---LLLALAASR--RSH-HDD--LSQ 164

Cs340270 109 FVG-RKSSQIFLIRQSYQARYKKQLDQD-IINID---PPHSYQK---ILVALAASH--KAH-NAD--ISQ 165

Pt13g04990 106 FVG-RKSSHMVLIKQAYYARFRRHLDQE-IINLE---PPHPYQK---ILVALATSH--KAH-QED--VSQ 162

Cp00161g00040 106 FVG-RKSSHIALMKQAYQARFKSQLEQD-IINID---PPHSFQK---ILVAFGASH--KAH-EMD--VSQ 162

Vv08g00710 102 FVW-RKSSQILLMKQDYGARFRRQMDQD-IINIE---PPHPYQK---ILVALMASH--KAH-HAD--VSQ 158

Gm13g26040 109 FVG-LPENVQKTLGPRYYQFGSKGERYI-IRNHS----IGLWEL---IIVALAASH--KAH-QAD--VNH 164

Os09g20330 111 VCT-RPSGEVALIRQAYQARYSASLERD-VSSRT----SGSLNE---VLLAFLGSSGSGYH-GGR--VDA 168

Cp00042g00810 47 ACT-RSSLELHFIKQAYNSRFNSNLEQD-MGTKL----NSGFKE---ILLAVMKSS--QYY-AGK--ADT 102

Os09g23160 110 ICS-RTPSQLQIMKQTYHAKFGTYLEHD-IGQRT----SGDHQK---LLLAYVGIP--RYE-GPE--VDP 165

Bd4g29680 110 ICS-RTPSQLQIMKQTYYAKFGTYVEHD-ISQQT----TGDHQK---ILLAYIGIP--RYE-GPE--VDP 165

Sb02g024090 110 ICS-RTPSQLQIMKQTYYARFGTYLEHD-IGHHT----SGDHQK---LLLAYVGIP--RYE-GPE--VDP 165

Zm02g30240 110 ICS-RTPSQLQIMKQTYYARFGTYLEHD-IAHHT----SGDHQK---LLLAYMGIP--RYE-GPE--VDP 165

Zm07g13390 110 ICS-RTPSQLQIMKQTYYARFGTYLEHD-IGHHT----SGDHQK---LLLAYVGIP--RYE-GPE--VDP 165

Bd3g36240 113 VCS-RTPSQLQIMKQTYRVRFGCYLEHD-ITERA----YGDHQR---LLLAYLG---------------- 157

Zm04g13650 110 VCS-RTPSQLQIMRQTYRARFGCYVEHD-VTERT----SGDHQR---LLLAYLAIP--RAE-GHE--VDP 165

Sb07g020760 110 VCS-RTPSQLAVVRHAYRARFGCHLEHD-VTERT----SGDHQR---LLLAYLAVP--RAEGGAV-VVDA 167

Os08g32970 110 VCS-RTPSQLLVVRQAYLARFGGGLEHD-VAVRA----SGDHQR---LLLAYLRSP--RYE-GPE-VVDM 166

Cs234810 90 LCS-RTSTQIQHVRQIYLSMFQSYIEHD-IEKSA----SGDHKK---LLLAYVSKP--RYE-GPE--IDR 145

Cs234800 110 ICS-RTPSQIQHFKQIYLAMFRSPLERD-IERTA----TGDHLK---LLLAYVSKP--RYE-GPE--VDR 165

Vv01g05380 110 ICS-RTPSQIQHFKQLYFAMFGVYLEQD-IEYQA----SGDHKK---LLLAYVTVP--RYE-GPE--VDR 165

Gm08g06100 109 ICS-RTPSQLHYLRQIYHSKFGVYLEHD-IETNT----SGDHKK---ILLAYVTTP--RHE-GPE--VNR 164

Mt8g107640 109 ICS-RTPSQLQYLRQIYHTRFGVYLDHD-IERNA----SGDHKK---ILLAYVSTP--RHE-GPE--VNR 164

Gm07g12030 110 ICS-HTPSQLQYLKQIYHSMFGVYLEHD-IQTNT---SPGDHQK---LLLAYISTP--RHE-GPE--VNR 166

Gm09g30190 110 ICS-RTPSQLQYLKQIYHSMFGVYLEHD-IQTNT---SPGDHQK---LLLAYISTP--RHE-GPE--VNR 166

Pt15g04350 110 ICS-RTPSQIQVFKQHYHAKFGIHLERD-IESCA----SGDHKK---LLLAYASMP--RYE-GRE--VDR 165

Pt12g03690 110 ICS-RTSSQIQVFKQHYYAKFGVHLEHD-IELRA----SGDHKK---LLLAYVSTP--RYE-GRE--VDR 165

Cp00003g03400 108 ICS-RTPSQIQLIKQHYHSQVGIHLEEE-IQQQT----PDDHQK---LLLTYVSTA--RYE-GPE--VGR 163

Gm07g28080 110 ICS-RTPSQLRRLKEVYLSTYHSYLEQD-IESKT----SGDHKK---LLLAYVSIP--RYE-GLE--LDH 165

Gm20g01460 101 ICS-RTPSQLRRLKEVYLSTYHSYLEQD-IENKT----SGDYKK---LLLAYVSIP--RYE-GPE--LDH 156

Cp00002g01210 110 ICT-RTTAQLRQIKQVYSKDYGTTPEHD-IESKC----YGDHKR---LLLAYLNTT--RYE-GPE--IDN 165

At1g68090 110 ICT-RSGSQLRQIKQVYSNTFGVKLEED-IESEA----SGNHKR---VLLAYLNTT--RYE-GPE--IDN 165

Pt10g10090 110 ICT-RISSQIRQIKQVYTPTFGTLLEYD-IGYHT----SGDHRK---FLLAYIDTT--RYD-GPE--IER 165

Pt08g13700 110 ICT-RTSSQIRQIKQVYTPTFGTRLEYD-IGCHT----SDDHKK---LLLAFIAIT--RYD-GPE--IDS 165

Sm271856 110 ICS-RTPSQLYYIRQAYQTKYHRSLDKD-IQSDT----SGDYRK---LLLAFASGQ--RPE-GPH--VDM 165

Sm167346 110 ICS-RTPSQLYYIRQAYQTKYHRSLDKD-IQSDT----SGDYRK---LLLAFASGQ--RPE-GPH--VDM 165

Sm124402 110 ICS-RTPSQLYYIRQAYQTKYHRSLDKD-IHSDT----SGDYRK---LLLAFASGQ--RPE-GPH--VDM 165

Sm94768 110 ICS-RTPSQLYYIRQAYQTKYHRSLDKD-IQSDT----SGDYRK---LLLAFASGQ--RPE-GPH--VDM 165

Sm227533 110 ICS-RTPSQLYYIRQAYQTKYHRSLDKD-IQSDT----SGDYRK---LLLAFASGQ--RPE-WPH--VDM 165

Pp1s219_3V6 109 ICT-RTPSQIYEIKQAYQAMYQQALESQ-VSGDT----SGDYRK---LLLALLRGS--RSE-TFS--VDS 164

Pp1s37_276V6 109 ICT-RTPSQHYAISQAYNAMFRHTLERK-IDGDT----SGNYRK---LLLALLRGN--RSE-TLA--VDP 164

Pp1s6_292V6 110 VCT-RNSAQIYLIKQAYYTMFNQTLENH-IDGTD---SPKRLVG---LMLALVRGN--RPE-NTS--VDR 166

Pp1s102_141V6 109 VCT-RNSAQLYLIKQAYYTMFNQT--------------PKRLVS---LLLALVRGN--RPE-NTP--VDR 155

Pp1s1_594V6 110 VCT-RNPTQIYAIKQAYYTMFNQTLENH-IDGTN---SHKRPVS---LLLALVRAS--RPE-NST--VDR 166

Pp1s61_299V6 111 LCT-RSSAQLYLIKQAYYSVFCQTLENH-LDG------SGFPKRQLALLLALARGS--RPE-NTT--VDR 167

Pp1s38_63V6 111 LCT-RTSKQIYLIKQAYYTMFNQTLESH-IDG------SGFPKRVLALLLALARGS--RPE-NTA--VDR 167

PsABK21977 111 LCS-RTPAETLRIREAYRGLYKACLEED-IAQET----VGPHQK---LLFTLAKAQ--RCP-SRD--VNI 166

PsACN40166 111 LCS-RTPAETLRIREAYRGLYKACLEED-IAQET----VGPHQK---LLFTLAKAQ--RCP-SRD--VNI 166

Cp00213g00130 110 ACT-RSSLELFKVKQAYHARYKRSLEED-VAYHT----KGDFRK---LLVPLVSTF--RYE-GEE--VNM 165

Bd3g58830 110 ACA-RTPAQLFAARQAYHERFKRSLEED-VAAHA----TGDFRK---LLVPLVSAY--RYD-GPE--VNT 165

Os02g51750 110 ACT-RTPSQLFAAKQAYHERFKRSLEED-VAAHI----TGDYRK---LLVPLVTVY--RYD-GPE--VNT 165

Sb04g027590 110 ACA-RTPAQLFAAKQAYHDRFKRSLEED-VAAHV----TGDFRK---LLVPLVSAY--RYD-GPE--VNT 165

Zm05g40790 110 ACA-RTPAQLFAVKQAYHDRFKRSLEED-VAAHV----TGDFRK---LLVPLVSAY--RYD-GPE--VNT 165

Bd1g45487 110 ACA-RGSGQLFAVRQAYHERFKRSLEED-VAAHV----TGAFRKV--LLVPLVSSY--RYE-GPE--VNT 166

Os06g11800 112 ACA-RGPAQLFAVRQAYHERFKRSLEED-VAAHA----TGDFRK---LLVPLISAY--RYE-GPE--VNT 167

Zm06g16450 110 ACT-RTSAQIFATRQAYHERFKRSLEED-IAAHV----TGDFRK---LLVPLVSTY--RYD-GPE--VNT 165

Sb10g007760 110 ACT-RTSAQVFAARQAYHERFKRSLEED-IAAHV----TGDFRK---LLVPLVSTY--RYD-GPE--VNT 165

Cs273000 115 ACT-RTSIELFKVRQAYQARFKRSVEED-VAYHT----SGDIRK---LLVPLISSL--QYE-GDE--VNK 170

At5g65020 110 ACT-RPALELIKVKQAYQARYKKSIEED-VAQHT----SGDLRK---LLLPLVSTF--RYE-GDD--VNM 165

Gm13g01870 110 ACT-RSSEQLFAARKAYHVLYKKSLEED-VAHHT----TGDFRK---LILPLVSSY--RYE-GDE--VNL 165

Mt5g063670 110 ACT-RSSDQLFFAKKAYHALHKKSLEED-VAYHT----TGDFRK---LLLPLVSSY--RYE-GDE--VNL 165

Vv18g03470 88 --------------QAYHARFKRSLEED-VAYHT----SGDFRK---LLVPLVGTY--RYE-GEE--VNM 130

Pt02g09420 110 ACT-RSSNELLLARQAYHARFKKSLEED-VAHHT----SGDFRK---LLFPLVSSY--RYD-GDE--VNM 165

Pt07g05300 110 ACT-RSSHDLFKVRQAYHARYKKSLEED-VAYHT----TGDFRK---LLVPLVSAF--RYE-GEE--VNT 165

Pt05g07550 110 ACT-RSSHDLFKARQAYHARYKKSLEED-VAYHT----TGDFRK---LLVPLVSAF--RYE-GEE--VNT 165

Gm05g31250 110 AST-RSSLDLLKAKQAYQARFKKSLEED-VAYHT----KGDIRK---LLVPLVSIF--RYE-GDE--VNM 165

Gm08g14460 110 AST-RSSLDLLKAKQAYQARFKKSLEED-VAYHT----KGDIRK---LLVPLVSTF--RYE-GDE--VNM 165

At1g35720 110 ACT-RTSTQLLHARQAYHARYKKSLEED-VAHHT----TGDFRK---LLVSLVTSY--RYE-GDE--VNM 165

At5g10230 110 ACT-RSALELFNAKQAYQARYKTSLEED-VAYHT----SGDIRK---LLVPLVSTF--RYD-GDE--VNM 165

At5g10220 110 ACT-RPSLEFFKTKQAYHVRYKTSLEED-VAYHT----SGNIRK---LLVPLVSTF--RYD-GNADEVNV 167

Cp00036g01250 98 ----RSSNQLLQAGQAYHARFKRSLEED-VAYHT----KENFRK---LLVPLVSSY--RYE-GDE----- 147

Cs217870 108 ACT-RTPRDLLLVKEEYHARFKRSIEED-VAHYT----TGDFRR---LLVPLVTAY--RYG-GPE--VNA 163

Cp00042g00660 29 ACT-RTSHEVFL---------------------------------------------------------- 39

PsABK22223 110 SCA-RSSSELWLVRQAYHVRYKKSLEED-IASHT----QGDFRK---LLVQLVSSY--RYE-GPE--VDM 165

Zm03g04200 110 ACA-NSSAELVAVKKAYHALYRRSLEED-VAARA---TAGNLRS---LLLALVSTY--RYD-GAD-SVDM 167

Bd2g13620 110 ACA-NSPTELLKVKQAYHALYKCSLEED-VAASA---PAGNLRS---LLLALVSTY--RYD-GEE--VDG 166

Os01g31270 110 ACT-NSSSELLA-----------------------------------LLLALVSTY--RYD-GDE--VND 138

Zm08g03950 110 ACATNSSAELVSVKQAYHVLYRRSLEED-VAARA----TGNLRS---LLLALVSTY--RYD-GDD-NVDA 167

Sb03g004990 110 ACANNSAAELVAVKKAYHALYKRSLEED-VAARA----TGNLRT---LLLAVVSTY--RYD-GDD-NVDM 167

Gm13g26960 109 ACV-LSADEVLAVKRAYHNRYKRSLEED-VATNT----TGDIRQ---LLVGLVTAY--RYD-GDE--VNA 164

Gm15g38010 109 ACV-LSAEEVLAVKRAYHNRYKRSLEED-VATNT----TGDIRQ---LLVGLVTAY--RYG-GDE--INA 164

Cs308090 110 SCI-YSPEELLGVRRAYQHRYKRSLEED-VAAST----NDDLRT---LLVGLVSAY--RYN-GAD--VDL 165

Cs308080 110 SCI-YSPEEFLGVRRAYQHRYKRSLEED-VAANT----HDDFRK---LLVGLVSAY--RYN-GGE--IDA 165

Gm13g26990 108 ATI-LSPEELLAVRRAYLNRYKHSLEED-VAAHT----SGHLRQ---LLVGLVTAF--RHV-GDE--INP 163

Gm15g38040 108 ATI-LSPEELLAVRRAYLNRYKHSLEED-VAAHT----SGHLRQ---LLVGLVTSY--RYV-GDE--INP 163

Mt8g038210 108 SAV-LSPEELLNVRRAYVKRYKHSLEED-LAAHT----SGHLRQ---LLVGLVTAF--RYV-GDE--INP 163

Mt8g038220 109 VSV-LSPEEVLAMRRAYHNRYKHSLEED-LAAHT----TGHLRQ---LLVGLVTSF--RYG-GAE--INP 164

Gm11g21480 109 ACV-LSAEELLAVRRAYHRRYKCSLEED-VAANT----TGNLRQ---LLVGLVTSY--RYE-GDE--INV 164

Mt3g018780 109 ASV-LSTEELLAVRRAYHNRYKRSIEED-VSAHT----TGHLRQ---LLVGLVSSF--RYE-GDE--INA 164

Gm04g27100 86 SCV-LSPEELFAVRRAYHNKYKRCLEED-VAANT----SGHLRQ---LLVGLVSSF--RYG-GSE--INA 141

Mt8g038180 109 ASV-LQPQELLAVRHAYHNRYKNSLEED-VAAHT----SGYHRQ---LLVGLVSSF--RYD-GVE--INP 164

Cp04842g00010 74 ----------------------------------------------------IQLL--MYD-GTE--VDG 86

Cp36671g00010 109 SCI-RSPEELIAIRRAYQIRYKCSLEED-VAAHT----SGDIRK---LLVALLTAF--RYIYGAE--THG 165

Pt03g19020 108 ACV-LSSEELLAVRRAYHARYKHSLEED-LAAHT-------------------TAF--RYE-GDE--INT 151

Mt3g018920 24 -------------SRAYHNRYKRSLEED-VATNN----NGYLRQ---LLVGLVSSF--RYG-GSE--VNA 67

Mt3g018790 109 SCV-SSPDELFNLRRAYHNRYKRSLEED-VATNT----NGHLRQ---LLVGLVSSF--RYD-GSE--VNA 164

Cp00157g00670 109 TCI-ASPDDLLAVKRAYRSRYKRSLEED-VASYT----TADIRT---LLVAVTSAY--RYD-GSE--VDE 164

Cs138380 109 ACV-QSAEDLLAVKRAYRFRFKRSLEED-VASCT----TGNMRK---LLVGVVSAY--RCE-GNE--IDE 164

Pt01g27650 109 ACV-GSPEDLLAVKRAYRFRYRHSLEED-VALHT----KGDIRK---VLVALVSAY--RYD-GHE--VDE 164

At5g12380 109 ACM-RSPEDMLAARRAYRCLYKHSLEED-LASRT----IGDIRR---LLVAMVSAY--KYD-GEE--IDE 164

Vv06g10680 109 AYM-KSPEELLAVKRAYQFLYKRSLEED-VASHT----TGDMRR---LLIAVVSVY--RYE-GEE--IDE 164

Cp00197g00010 109 SCT-RSPEELFAIRRAYQ-----------------------------LLIALLTTF--RYN-GNE--IND 143

Gm11g21460 109 SCV-LSPEELFVVRRAYHNKYKRSLEED-VAANT----SGHLRQ---ILVGLVSSF--RYG-GSE--INA 164

Gm13g27000 109 VCV----------RRAYHNRYKHSLE-D-VAAHT----TDHVRQ---ASMGVMRSM--QDW--------- 148

Gm15g14350 109 VCT-RTSEEFLAAKRSYQFQYKHCLEED-VASKT----IGDIRR---LLVAVISTY--RYD-GDE--FDE 164

Os05g31750 102 ACT-RTAEELLGARKAYQALFHHSLEED-VAYRA---RDKPYCG---LLVGLVSAY--RYE-GPR--VSE 158

Bd2g26760 102 ACT-RSAEDLLGARKAYQALFHHSLEED-VAFHA---KDKPYCS---LLVGLVSAY--RYE-GPK--VNE 158

Zm06g23270 104 ACA-RSADELLGARRAYQALFHRSLEED-VAHRA---RDKPYCS---WMDGAADAR--ALS-GSK----- 157

Bd1g62130 106 ACT-RSAEELLGARRAYHALYSRSLEED-VAYRL---KETEHAG---LLVGLVAAY--RYE-GAR--VSE 162

Zm01g15800 108 ACT-RTADDLLGARRAYQALYHRSLEED-VAYRV----RDANAS---LLLGLVSAY--RYE-GAR--VNE 163

Sb01g035050 111 ACT-RAADDLLGARRAYQALYHRSLEED-VAYRV----RDANAS---LLVGLVSAY--RYE-GAR--VSE 166

Mt8g038170 100 ACT-RSSEELLGARKAYHSLFDHSIEED-VASHI----HGNDRK---LLVALVSAY--RYE-GTK--VKD 155

Cs308100 101 ACT-RTSDELLGARKAYHSLFDHSIEED-VASHL----NGPERK---LLVALMSAY--RYE-GPK--YKE 156

Gm13g27010 100 ACT-RSSEELLGARKAYHSLFDHSIEED-VASHI----HGIERK---LLVALLSAY--RYE-GTK--VKD 155

Gm15g38060 100 SCT-RSSEELLGARKAYHSLFDHSIEED-VASHI----HGIERK---LLVALLSAY--RYE-GTK--VKD 155

Pt01g06030_ 97 ACT-RSSEELLGARKAYHSLFDQSIEED-VATHI----HGSERK---LLVALVSAY--RYE-GPK--VKE 152

Vv00g25070 100 AST-RSSEQLLGARRAYHSLFDHSIEED-VAYHI----NDSCRK---LLVGLVSSY--RYE-GPK--VNE 155

At2g38750 106 SCT-RSAEDLLGARKAYHSLFDQSMEED-IASHV----HGPQRK---LLVGLVSAY--RYE-GNK--VKD 161

Zm08g13570 112 ACA-STPDHLVAVRKAYRESYPASLEED-VAACPL-YKDPRVKQ---FLVRLVSSY--RYS-GDL--VDD 170

Bd2g26770 112 ACA-SSPDHLVAVRKAYCSAYESSLEED-VAACSL--YKDPLKQ---FLVRLVSSY--RYAGGEH--VDD 170

Zm06g23280 114 ACA-SAPDHLVAVRKAYREAYSASLEED-VAACPL-YKDPLLKQ---FLVRLVSSY--RYS-GEL--VDD 172

Sb09g018980 111 ACA-STPDHLVAVRKAYREAYSASLEED-VAACPLYNKDPLLKQ---FLVRLVSSY--RYS-GEL--VDD 170

Os05g31760 114 ACA-SSPDHLVAVRKAYRAAYASSLEED-VASCSL--FGDPLRR---FLVRLVSSY--RYG-GGG--VDG 171

Sb02g041850 88 SCA-STPDHLMAVRNIYRKLFSSSVEED-VASSPA--LQEPLKK---MLLRLVSSY--RYA-GEH--VDM 145

Bd1g62120 26 -----------------------------------------GNL----LVSLVRSY--RCA-EEA--VDV 45

Sb01g035040 38 ACA-SAPDHLIAVRRAYRSLFGCSLEED-VAACPA--LQDPLRK---LLVSLVRSY--RCE-TER--VDE 95

Bd1g18990 87 SCA-SAPDHLVAVRRAYASLFGCSLEED-LASSVS--FQEPLKK---LLVGLVTSY--RYD-GDQ--VDE 144

Os07g46550 87 -----------------------------------------------MLVRLVSSY--RYE-GDECVVDM 106

Pt01g06020 113 TCA-SSPNHLQEVRQAYCSIFDCSLEED-IVSAV----PLPLRK---ILVAVASSY--RYD-KEL--VDT 168

Gm15g38070 112 ACA-STPNHLVAVRQAYCSLFDCSLEED-IIASV----APALRK---LLVSLVSSF--RYD-KVA--VNL 167

Gm13g27020 113 ACA-STPNHLVAVRQAYCSLFDCSLEED-IIASV----APPLRK---LLVSLVSSF--RYD-KVA--VNL 168

Mt8g038150 113 ACA-SSPNHLMAVRQAYCSLFDCSLEED-IIASV----SQPLTK---ILVGLVSSF--RHD-KVT--VNL 168

Vv00g25060 113 ACA-SSPHHLMSVRQAYCSLFESSLEED-ITANV----SLPLKK---LLVGLVSSY--RYD-KEM--VDL 168

Cp00197g00020 113 ACA-SSPHHLMAVRQAYSSLFDCSLEED-IVSAV----PPLIAK---VLVALVRSY--RYD-KEL--VDP 168

At2g38760 115 SCT-TSPNHLIAVRKAYCSLFDSSLEEH-IASSL----PFPLAK---LLVTLASTF--RYD-KDR--TDA 170

Vv00g00800 113 ACA-SSPNHLMAVRQAYCSLFDCSLEEA-ITSKV----SSSLQK---LLLGLVSSY--RYD-REL--VDL 168

Vv03g02080 113 ACA-SSPDHLMAVRQAYCSLYECSLEED-ITSNI----STSLQK---LLVGLVSSY--RHD-REL--VDF 168

Vv00g00650 113 ACA-SSPDHLMAVRQAYFSLYECSLEED-ITSNI----STSLQK---LLVGLVSSY--RHD-REL--VDF 168

Vv00g00720 2 ------------------------LQTN---------------IK--LLVGLVSSY--RHD-REL--VDF 25

Vv00g00760 20 --------RMLSGRRHHIQHLNFSPKGD-------------------ISLGLVSSY--RHD-REL--VDF 57

Vv00g00750 113 ACA-SSPNHLMAVRQAYYSLFDCSLEEA-ITSKV----SSSLQK-------VVHSL--RYT--KL--VDL 163

Vv00g00710 113 ACA-SCPNHLMAVRQAYCSLFDCSLEEA-ITSKV----SSSLQK---LLLGLVSSY--RYD-REL--VDL 168

Vv00g00660 113 ACA-SSPNHLMAVRQAYCSLFDCSLEEA-ITSKV----SSSLQK---LLLGLVSSY--RYD-REL--VDL 168

Ot24272 112 LAN-KTHEQIEEFREAYKVATGELLRER-IRDET----TGLFES---KLFRETLMG-----------LLT 161

MRCC299_03g01870 111 MVT-CTHQVMYDTRKAYGRLYNLDLARD-MSGEWA--ISGDFKN---ILCALVKKH--PDN---I--ETD 166

Cs307980 84 TCT-REFCDVSAAKDVYHHLYKSLLEFD-LSRYI----VGPEQT---LLNSLLNTK--RCKETNK--EEE 140

Cs307970 117 VCT-RPFADFLAIKYLYGKLFKSDLLFD-LDQHV----PGKAVR---CLINLFSIE--RRQDIIK--GEE 173

[Consensus_aa:](http://prodata.swmed.edu/promals3d/info/consensus.html) *h*ts.psspp*l*b.*h***+**p*h***Y**...*@*...**L-**p**D**.*l*..p*h*....ss.*h*pp...*l***L***l*s*hh*ps...**+***h*p...p..*l*s.

[Consensus_ss:](http://prodata.swmed.edu/promals3d/info/consensus_ss.html) hhh hhhhhhhhhhhhhhh hhhh hhhh hhhh hhhhhhh h

Conservation: 77 7 5 55 76 56 6 6

Bd4g31920 163 HIAKCDARRLYDAKKGGT------GSVDEATILEMFSKRSIPQVRLAFSSYK--HIYGHDYTKALKK-NV 223

Os09g27990 165 HIAKCDARRLYDAKNSGM------GSVDEAVILEMFSKRSIPQLRLAFCSYK--HIYGHDYTKALKK-NG 225

Zm02g31380 165 HVAKCDARRLHDTKNSGAGA-GSGSVVDEAVILEMFSKRSIPQLRLAFCSYK--HIYGHDYTKALKI-NG 230

Sb02g026390 165 HVAKCDARRLHDTKNSGA-----GSVVDEAVILEMFSKRSIPQLRLAFCSYK--HIYGHDYTKALKI-NG 226

Cs340270 166 HIAKCDARKLYETVKDNS------GAIEEAFVLEMLTKRSIPQLKLTFSCYQ--HIFGHNFTKDLKF-RN 226

Pt13g04990 163 HIAKCDARRLYEAGEGSS-----QGAVEEAVVLEILSKRSIPQTKLTLSSYK--HIYGHEYTKSLKN-AK 224

Cp00161g00040 163 HVAKCDARRLYEVGEGSA------GGIEEAVVLEIFSKRSIPQLKLTFSCYK--HIYGYDYIKSFKR-EN 223

Vv08g00710 159 HIAKCDARRLYEAGVGKS------GGTEEAVVLEILSKRSIPQLKLTFSCYK--HIYGHDYTKLLKK-EN 219

Gm13g26040 165 HISKCDARRLYETGEGSL------GTVIEAVVLEILSKRSIPQLKLTFFSYK--HIYGHDYTKSIKR-GK 225

Os09g20330 169 TMAMCDAKTLYEAVEISA------ARVDQRSVLQLLRHRSGDQLRAVLASYR--RLYGQELARALKR-KD 229

Cp00042g00810 103 SMAMCDAKTLYEAVETG-------KTIDQRSIVLIMSQRNTGQIKAILSSYR--QLYGHEFSKAIKQ-SK 162

Os09g23160 166 TIVTHDAKDLYKAGEKR-------LGTDEKTFIRIFTERSWAHMASVASAYH--HMYDRSLEKVVKS-ET 225

Bd4g29680 166 TIVTHDAKDLYKAGEKK-------LGTDEKTFIRIFTERSWAHMAAVASAYH--HMYDRSLEKVVKS-ET 225

Sb02g024090 166 TIVTHDAKDLYKAGEKR-------LGTDEKTFIRVFTERSWAHLASVSSAYH--HMYDRKLEKVVKS-ET 225

Zm02g30240 166 TIVTHDAKDLYKAGEKR-------LGTDEKIFIRVFTERSWAHLASVSSAYH--HMYDRKLEKVIKS-ET 225

Zm07g13390 166 TIVTHDAKDLYKAGEKR-------LGTDEKTFIRVFTERSWAHLASVSSAYH--HMYDRKLEKVIKS-ET 225

Bd3g36240 158 ---------LYKAGEKR-------LGTDERTFIRIFSERSWAHLASVASAYQ--HMYARSLEKAVKS-ET 208

Zm04g13650 166 STVTLDARDLYKAGERR-------LGTDERAFIRIFSQRSWAHMAAVARAYH--HMYDRPLERAVKS-ET 225

Sb07g020760 168 STVALDARDLYKAGERR-------LGTDERAFIRVFSERSWPHMAAVARAYH--HMYDRSLESAVKS-ET 227

Os08g32970 167 AAAARDARELYRAGERR-------LGTDERTFIRVFSERSAAHMAAVAAAYH--HMYDRSLEKAVKS-ET 226

Cs234810 146 NIVEKDAKTLYKAGEKR-------WGTDEQKFIQIFSESSRAHLAAVAYTYK--QSYSNSLEKAIKS-ET 205

Cs234800 166 ALVDKDAKSLYKAGEKR-------LGTDEDKFIKIFSERSRAHLSAVSHAYK--HSYGNSLKEVIKK-ET 225

Vv01g05380 166 AMVEKDAKALYKAGEKK-------LGTDENTFIRIFSEKSRAHLAAVSTAYH--SVYGNSLQKAVKS-ET 225

Gm08g06100 165 EMAEKDAKVLYKAGEKR-------LGTDEKTFVQIFSERSAAHLAAITSYYH--SMYGHSLKKAVKK-ET 224

Mt8g107640 165 EMAENDAKVLYKAGEKK-------LGTDEKTFVQIFSQRSAAQLAAINHFYH--ANYGHSLKKAIKN-ET 224

Gm07g12030 167 EIAQKDAKALYKAGEKK-------LGTDEKTFIHIFSERSAAHLAAVSSYYH--DMYGHSLKKAVKN-ET 226

Gm09g30190 167 EIAQKDAKGLYKAGEKK-------LGTDEKTFIHIFSERSAAHLAAVSSYYH--DMYGHSLKKAVKN-ET 226

Pt15g04350 166 EMVVKDAKALYKAGEKK-------WGTDEKTFIHIFSERSAAHLAAVDSAYH--DMYGNSLNKVIKK-ET 225

Pt12g03690 166 NMVEKDAKALYKAGEKR-------LGTDEMTFIRVFSERSAAHLAAVDSAYH--NMYGNSLKKAIKK-ET 225

Cp00003g03400 164 EEAVKDAKALFKAGEKK-------WGTDEKTFIRIFSERSRAHMVAVDAAYH--EMYGNSLKKAVKK-ET 223

Gm07g28080 166 IIVQEDAKQLYKSGEKR-------IGTDEKMFIKIFSEKSGAHLAAVNSTYI--ASYGHSLEKAIKK-ET 225

Gm20g01460 157 IIVQEDAKQLYKSGEKR-------IGTDEKMFIKIFSEKSSTHLAAVNSAYI--ASYGHSLEKAIKK-ET 216

Cp00002g01210 166 VLVEADATTLHTAISKK-------HGGEDKVFIQIFSERSKAHLAALGSAYR--KMYGKFLGKAIRH-ET 225

At1g68090 166 ASVENDARTLKSAVARK-------HKSDDQTLIQIFTDRSRTHLVAVRSTYR--SMYGKELGKAIRD-ET 225

Pt10g10090 166 VLVEEDAIAISKIEVKK-------SGMDESTFIQIFTERSSAHLAALASAYH--KMFRKELRKTIKR-ET 225

Pt08g13700 166 VLVEDDAKAINKIGVKK-------SGMDESTFIQIFTERSSAHLIALASVYH--KMFGKELRKTIKR-EA 225

Sm271856 166 HLADADARELYRAGEGR-------LGTDESTFIRVFSTRSAAQLHAAFAAYK--HLYKRDIDKAIKR-ET 225

Sm167346 166 HLADADARELYRAGEGR-------VGTDESTFIRVFSTRSAAQLHAAFAAYK--HLYKRDIDKAIKR-ET 225

Sm124402 166 HLADADARELYRAGEGR-------LGTDESTFIRIFSTRSAAQLHAAFAAYK--HLYKRDIDKAIRR-ET 225

Sm94768 166 HLADADARELYRAGEGR-------LGTDESTFIRIFSTRSAAQLHAAFAAYK--HLYKRDIDKAIKR-ET 225

Sm227533 166 HLADADARELYRAGEGR-------LGTDESTFIRIFSTRSAAQLHAAFAAYK--HLYKRDIDKAIRR-ET 225

Pp1s219_3V6 165 NLALADAHDLYRAGEAR-------LGTNEDIIIHILTTRSPAQLNLALQYYR--QTYGHEFMKAVKS-ET 224

Pp1s37_276V6 165 NFALADAHALYQAGEAR-------LGTDEDTFIHILTTRSPAQLNMTLQYYR--QIYGRDFEKSIKR-ET 224

Pp1s6_292V6 167 HIALNDAHQLNKVFTGK--------VGDEDTLIRIFCTRSAQQLTATLNYYH--QHYGHDFEESLIN-EN 225

Pp1s102_141V6 156 HIALNDAHQLHKVVIGK--------GGNEDTLVRILCTRSIQQLTATFNYYH--QHYGRELEQSLTR-GG 214

Pp1s1_594V6 167 HIALNDAHQLNKVFTIVG------KVGNEDTLIRIFCTRSAQQLTATLNYYH--QHYGHDFEQSLTR-EN 227

Pp1s61_299V6 168 HIALTDAHQLNKVCSGK--------LGNEETLIRIFSTRSPYQLTATMNFYE--QHYGHDFEKALSK-KD 226

Pp1s38_63V6 168 HFALSDAHHLNKVCTGK--------IGNEEMLIRIFTTRSSYQLSATMNYYQ--QHYGHDFEKVLSK-QG 226

PsABK21977 167 CQAKCDAKRLYGAREGR-------IGIDEGAIVKLLSDRNLNHLRAAFGYYK--QFYGHDILKALRR-ET 226

PsACN40166 167 CQAKCDAKRLYGAREGR-------IGIDEGAIVKLLSDRNLNHLRAAFGYYK--QFYGHDIL-------- 219

Cp00213g00130 166 TLAKNEAKTLHEKISDK--------AYNDEEVIRIISTRSKAQLNATLNHYN--NGYGNAINKDLKS-DP 224

Bd3g58830 166 SLAHSEAKILHEKINDG--------AYGDDEIIRILTTRSKAQLLATFNSYN--DQFSHPITKDLKA-DP 224

Os02g51750 166 SLAHSEAKILHEKIHDK--------AYSDDEIIRILTTRSKAQLLATFNSYN--DQFGHPITKDLKA-DP 224

Sb04g027590 166 SLAHSEAKILHEKIDKK--------AYSDEEIIRILTTRSKAQLLATFNNYK--DQFGHAINKDLKA-DP 224

Zm05g40790 166 SLAHSEAKILHEKIHKK--------AYSDEEIIRILTTRSKAQLLATFNSYK--DQFTHAINKDLKA-DP 224

Bd1g45487 167 RLAHSEAKILHEKIEHK--------AYGDDEIIRILTTRSKAQLLATFNHYN--DAFGHPITKDLKA-DP 225

Os06g11800 168 KLAHSEAKILHEKIQHK--------AYGDDEIIRILTTRSKAQLIATFNRYN--DEYGHPINKDLKA-DP 226

Zm06g16450 166 RLAHSEAKLLHEKIHHK--------AYSDDEIIRILTTRSKPQLIATFNHYN--DAFGHRINKLLYK-IV 224

Sb10g007760 166 RLAHSEAKLLHEKIHHK--------AYSDDEIIRILTTRSKPQLLATFNHYN--DAFGHRINKDLKA-DP 224

Cs273000 171 TLAKSEAKILHEKIAGK--------EYNHDEVIRILTTRSKAQLLATLNHYN--NEYGNAINKDLKA-DP 229

At5g65020 166 MLARSEAKILHEKVSEK--------SYSDDDFIRILTTRSKAQLGATLNHYN--NEYGNAINKNLKE-ES 224

Gm13g01870 166 TLAKTEAKLLHEKISNK--------AYNDDDFIRILATRSRAQINATLNHYK--DAFGQDINKDLKA-DP 224

Mt5g063670 166 TIAKAEAKILHEKISKK--------AYNDDDFIRILATRSKAQINATLNHYK--DAFGKDINKDLKE-DP 224

Vv18g03470 131 TLAKSEAKILHEKISEK--------AYNHEDVIRILATRSKAQINATLNHYK--NEFGNDINKDLKT-DP 189

Pt02g09420 166 TLAKSEAKMLHEKISNK--------AYSDEELIRILATRSKAQINATLNQYK--NEFGNDINKDLKA-DP 224

Pt07g05300 166 ILAKSEAKILHEKISDK--------AYSDEEIIRILTTRSKAQLNATLNHYN--NAFGNAINKNLKE-EA 224

Pt05g07550 166 MLAKSEAKILHEKISDK--------AYSDDEIIRILTTRSKAQLNATLNHYN--NAFGNAINKNLKE-DA 224

Gm05g31250 166 TLAKSEAKLLHEKIAEK--------AYNDEELIRILSTRSKAQLTATLNQYN--NEFGNAINKDLKT-DP 224

Gm08g14460 166 TLAKSEAKLLHQKIAEK--------AYNDEDLIRILSTRSKAQLTATLNQYN--NEFGNAINKDLKT-DP 224

At1g35720 166 TLAKQEAKLVHEKIKDK--------HYNDEDVIRILSTRSKAQINATFNRYQ--DDHGEEILKSLEEGDD 225

At5g10230 166 TLARSEAKILHEKIKEK--------AYADDDLIRILTTRSKAQISATLNHYK--NNFGTSMSKYLKE-DS 224

At5g10220 168 KLARSEAKTLHKKITEK--------AYTDEDLIRILTTRSKAQINATLNHFK--DKFGSSINKFLKE-DS 226

Cp00036g01250 148 --------KIHQKISEK--------AYDSDDLVRILATRSKVQIIATLNRYK--KEFGNDVNKDLKT-DP 198

Cs217870 164 TLATSEAKILHDKITEK--------AYNDEELIRIISTRSKAQLNATFNHYN--DQFGNAISKDLKT-DP 222

Cp00042g00660 40 ---------------------------------------------------------------ELKT-DP 45

PsABK22223 166 RLAKSEAKQLHEAIEDK--------AFGNEEFIRIITTRSKAQLNATFNNYK--DEYGHHINKDLKN-EK 224

Zm03g04200 168 ELARSEAKAVHEAVRDGG-----GAG-GHEELIRVVGTRSKAQLRATFGCFK--DEHRRSVAKALPR-GT 228

Bd2g13620 167 GLARSEAELIHEAVKNGE-----NGTTDDGELIRILGTRSKAQLGATFSCFR--DEHGTTLTK------- 222

Os01g31270 139 ALAKSEAKILHETVTNG--------DTDHGELIRIVGTRSRAQLNATFSWFR--DERGTSITKALQH-GA 197

Zm08g03950 168 ELARSEAKIVHEAVR-NSAG-AAGGRHDHEELIRVLGTRSKAQLRATFSCFKDQDEHRRSVTKALPR-GA 234

Sb03g004990 168 ELARSEAKIVHEAVRNGGG----GAAGGHDELIRVVGTRSKAQLRATFACFK--DEHRSSVTKALPR-GD 230

Gm13g26960 165 KLAKTEADILHESIKEK--------KGNHEEAIRILTTRSKTQLLATFNRYR--DDHGASITKKLLD-NA 223

Gm15g38010 165 KLAKTEADILHESIKEK--------KGNHEEAIRILTTRSKTQLLATFNRYR--DDHGASITKKLLD-NA 223

Cs308090 166 SLAKSEAERLERAIKDK--------TFYHEDVVRILTTRSRPQLVATFNHYK--DAYGISISEQLSSDKA 225

Cs308080 166 KLAKSEAEILERAVKDK--------AFNHEDVIRILTTRSKAQLIATFNHYK--DANGISISKQLGQDRD 225

Gm13g26990 164 KLAQSEAEILHDAVKEK--------KGSYEETIRVLTTRSRTQLVATFNRYR--EIHGTSISKKLVD-EG 222

Gm15g38040 164 KLAQTEAEILHDAVKEK--------KGSYEETIRVLTTRSRTQLVATFNCYR--EIHGTSISKKLVD-EG 222

Mt8g038210 164 KLAQTEAGILHESVKEK--------KGSHEEAIRILTTRSKTQLIATFNRYR--ETHGTSITKKLLD-EG 222

Mt8g038220 165 KLAKTEADILHESIKEK--------KGNHEEAIRILTTRSKTQLLATFNRYR--DDHGISITKKLLD-NA 223

Gm11g21480 165 KFSQTEANVLHESVKEK--------KGNSEEVIRILTTRSKTQLVATFNRYR--DEHGISISKKLLD-QT 223

Mt3g018780 165 KLAQTEANIIHESVKEK--------KGNNEEVIRILTTRSKTQLVATFNRYR--DEHGISISKKLLD-QT 223

Gm04g27100 142 KLAQSEADALHEAIKNK--------NKSNDEIIRILTTRSKTQLVATFNRYR--DDHGIAITKKLSD-EG 200

Mt8g038180 165 ILAKHEADILHEAVKNK--------KGNIEEVIRILITRSKTQLKATFNRYR--DDHGFSISKKLLN-EA 223

Cp04842g00010 87 RLANSEADILRDVIKDK--------EYNHEEVVRIVSTRSKPQLLATFNRYR--EEPGTSITKELLG-ES 145

Cp36671g00010 166 RLANSEAEILRDAIIDK--------EYNHEEVIRIVSTRSKLQLLATFNRYR--EEHGTSITKELLD-DS 224

Pt03g19020 152 RLTNSEADILHDAIKDK--------AFNHEDVIRILTTRSKAQLMATFNRYR--DDHGSSITKDLLD-EP 210

Mt3g018920 68 SLAQCEADMLHEAIKHK--------NHNHEEVIRILTTRSKTQLVATFNCYR-------HFLKKLSD-EG 121

Mt3g018790 165 SLAQCEADMLHEAIKNK--------NYNHEEVIRILTTRSKTQLVATFNCYR--HDHGIAITKKLSD-EG 223

Cp00157g00670 165 AVAHAEAITLHDEIQRN--------PLKHEEIIRVLGTRSKAQLNATFNIYK--DIYGTSITKNLLG-DP 223

Cs138380 165 NMAELEANIIDDEIKGK-------GLKNNEEMIRIVSTRSKPQLHATFNRYR--DIHATSITKGLIG-DS 224

Pt01g27650 165 DLAISEAGLLHDDVYGK--------AFNHDELVRVLTTRSKAQLNATFNRYQ--DIHGKSITKGLLG-DP 223

At5g12380 165 MLAQSEAAILHDEILGK--------AVDHEETIRVLSTRSSMQLSAIFNRYK--DIYGTSITKDLLN-HP 223

Vv06g10680 165 GVAHSEANILGDEMQGG--------ALKGEEIIRILSTRSKAQLIATFNNYK--QIHGTSITKSLRG-DP 223

Cp00197g00010 144 KLASSEADILRDAIKDK--------VYNHDEVVRIISTRSKAQLSATFNCHK--EKQGAFISEELLG-DL 202

Gm11g21460 165 KLAQSEDDALHEAIKNKNKNNLWLLSIATGMIMALPSLRVVSFLTCSFLPWQ--KLFDE----------G 222

Gm13g27000 149 --QNLKLIFFMRPSKTK-------KGHHEEVIGGSLVQEARPNLWQLSTASK----MKITFLSKLLE-ET 204

Gm15g14350 165 NLAHLEANILHQVIENK--------AFNDDEIIRILCTRSKKQLCATFSTFR--NVYGTTITKGLST-NP 223

Os05g31750 159 ETARAEAKALVAAVK-SAGH-AAAKLVENDDVVRILTTRSKPHLVETFKHYK--EIHGRHIEEDLGH--- 221

Bd2g26760 159 DTAKAEAKALGAALKKKE-----AAAVENGEVVRILTTRSKPHLVETFKHYK--ELHGKHIHEDLGS--- 218

Zm06g23270 158 ----------------------------------------APLLLT------------------------ 163

Bd1g62130 163 DLATEEANAISAK------------PGNNEVLARVLATRSKPQLRATFRIYR--EIHGKPLEEDLIA-VG 217

Zm01g15800 164 DLATEEAKALAAAVR-AAPA-AATKLVQNEQVVRVLVTRSKPQLGATFRVYM--ELHGKPLEEELPA--- 226

Sb01g035050 167 DLATEEAKALAAAVRAAPA----AKLVQNEQVVRVLATRSKPQLRATFRVYM--ELHGKPLEEDLAA--- 227

Mt8g038170 156 DTAKSEAKTLSNAIKNAQN----KPIVEDDEVIRILATRSKLHLQAVYKHYK--EISGKNLEEDLN---- 215

Cs308100 157 EIAKSEAKKFAHSIK-EANS-KKSSLIEDEEIVRILSTRSKHFLHALHKHYN-EISAGRSIDEDLHG--- 220

Gm13g27010 156 DTAKSEAKTLSNAIKNAHK----KPLNEDDEVIRILATRSKLHIQAVCKHYK--EISGKNLDEDLDD--- 216

Gm15g38060 156 DTAKSEAKILSNAIKNAHK----KPINEDDEVIRILATRSKLHLQAVYKHYK--EISGKNLDEDLDD--- 216

Pt01g06030_ 153 DAAKSEAKILANAIKNGNK----KNPIEDEEVIRILSTRSKAHLKVVYKHYK--EVSGNNIHEDLDA-SD 215

Vv00g25070 156 EIAKSEAKTLFAAIKNADK----KNPIEDEEVVRILTTRSKPHLKAIFKHYK--EINGKNIDEDLDD--- 216

At2g38750 162 DSAKSDAKILAEAVASSG-----EEAVEKDEVVRILTTRSKLHLQHLYKHFN--EIKGSDLLGGVSK--- 221

Zm08g13570 171 ELARAEAAELHDAVVAR-------KQLLHGQVVRIVSSRSKQQLQATFERYR--QDRGKAFDEVLEE-RR 230

Bd2g26770 171 ELARAEAAELHGAVVAQ-------KQPLHGDVVRVISSRSKPQLKATFQHYK--QHHGKSFDEVLEG-NR 230

Zm06g23280 173 ELARAEAAELHDAVVAR-------KQPLHGDVVRVVSSRSKAQLKATFERYR--LDHGKAVDEVLEE-RR 232

Sb09g018980 171 ELARAEAAELHDAVVAR-------KQPLHGDVVRIVSSRSKPQLKATFERYR--QGHGKAIDEVLEEERR 231

Os05g31760 172 ELAIAEAAELHDAVVGRG------QALHGDDVVRIVGTRSKAQLAVTLERYR--QEHGKGIDEVLDG-RR 232

Sb02g041850 146 DVAKLEAAQLSEAIREK-------RL-HGDEVARIISTRSKPQLRATFQQYK--DDQGTDIVEDIGS-SN 204

Bd1g62120 46 GIARLEAAQLAEAIRKK-------KQPHADEVVRIVSTRSKAQLRATFQCYK--QDHGSYIEEDIN--NC 104

Sb01g035040 96 DVARMEAAQLAEAIRKR-------RQPHGDEVARIVSTRSKHQLRATFQLYK--QEHGTDVDEDIT--KH 154

Bd1g18990 145 ATAAAEAALLCEAVRRK-------KQPHGEDVVRVISTRSKAQLAATFGLYR--AHHGTELVEDIE--SR 203

Os07g46550 107 DVVRMEASQLAEAIKKKK------QPRGEDEVVRIVTTRSKSQLRATFQRYR--EDHGSDIAEDID--SH 166

Pt01g06020 169 KVANAEAAKLHEVIKSK--------KLDQDDIILILSTRNFHQLRATFACYN--QNFGNSIDQDIKS-CG 227

Gm15g38070 168 EVAKEEASKLHEAINSK--------QLDNDHIIWILSTRNLFQLRETFACYN--NLYGNTLEQDIKK-CG 226

Gm13g27020 169 EVAKEEASKLHEAINCK--------QLEDDHIIWILSTRNFFQLRETFACYN--NLYGNTLEQDIK--CG 226

Mt8g038150 169 EVAKSEAEKLHEAINNN--------KLDDDHFVWILSTRNVFQIRETFASYK--QLYGKTFEEDIKT-CG 227

Vv00g25060 169 NVAKLEAAKLHEAIKKK--------QLDHDDVVWILSTRNVFQLQATFEFYK--QNYGNSIDQDIKS-FG 227

Cp00197g00020 169 NVANSEAVLLHEAIQKK--------KLDLDHVLYILGTRNFYQLRETFKSYK--EKFKNPVEKDIKN-CG 227

At2g38760 171 EVATIEAAMLREAIEKK--------QLDHDHVLYILGTRSIYQLRETFVAYK--KNYGVTIDKDVDG-CP 229

Vv00g00800 169 NVAKSEATKLHEAIEKK--------QLDRDEVMWILSTRNFFQLRATFKHYK--QNYQVPIYQAIMS-SG 227

Vv03g02080 169 NLAKSEADKIHEAIEKN--------QLDHDDVVWILTTRNFFQLRATFVCYK--QSYEVAIDQAINS-SG 227

Vv00g00650 169 NLAKSEATKLHEAIEKK--------QLDHDDVVWIMTTRNFFQLRATFVCYK--QSYEVAIDQAINS-SG 227

Vv00g00720 26 NLAKFEAAKLHEAIEKK--------QLDHDDVVWILTTKNFFQLRATFVCYK--QSYEVAIDQAINS-SG 84

Vv00g00760 58 NLAKSEVAKLHEAIEKN--------QLDHDDVVWILTTRNFFQLKATFVCYK--QSYEVAIDQAINS-SG 116

Vv00g00750 164 NVAKSEAAKLHEAIEKK--------QSDRDEVMWILSTRNFFQLRATFKHYK-------QNYQAIMS-SG 217

Vv00g00710 169 NVAKSEAAKLHEAIEKK--------QLDRDEVMWILSTRNFFQLRATFKHYK--QNYQVPIYQAIMS-SG 227

Vv00g00660 169 NVAKSEAAKLHEAIEKK--------QLDGDEVMWILSTRNFFQLRATFKHYK--QNYQVPIYQAIMS-SG 227

Ot24272 162 PREEQIAIYLQEAFGMW--------ANDDWGLISMLVHRTEEEKELIRTKYT--EHTGGDLIADIRS-KC 220

MRCC299_03g01870 167 PDYDADIQILHDAVEGL--------GTDEDAIIGVLRNKTEEQLQMLQRKYD--ATHCEDLKLRLKS-ET 225

Cs307980 141 KIVMLDAETLAKAFNDKS-----EVYIENREIINILMYRSISHLRAVFEQCK------------------ 187

Cs307970 174 KCLRKDITTLQNATSGEP-----QTRICIKHIVSILTQRSIGHLRNMYRFCQ--PEMRRQPKSSLWI--- 233

[Consensus_aa:](http://prodata.swmed.edu/promals3d/info/consensus.html) .*hh*p.**-A**..**L**.c*h*.p.p..........ppp.*hl*p*lh*sp**R**o..p**L**.*hhh*..**Y**p..p.*@*spp*h*.p.*l*.p.ps

[Consensus_ss:](http://prodata.swmed.edu/promals3d/info/consensus_ss.html) hhhhhhhhhhhhhhh hhhhhhhhhh hhhhhhhhhhhh hhh hhhhhhh h

Conservation: 6 6 6 5 66 5 5 6 6 5 5 65 6

Bd4g31920 224 FG----EFEES--LSVVVKCIYSPSKYYCK-LLQKSMQR--PES--NKRLVTRAILGSDD--VGMDKIKL 280

Os09g27990 226 FG----EFEQS--LRVVVKCIYNPSMYFSK-LLHRSLQC--SAT--NKRLVTRAILGSDD--VDMDKIKS 282

Zm02g31380 231 SG----EFEGP--LRVVVKCIYNPSKYYSK-LLHRSMLP--AAT--DTRMVTRAILGSDD--VGIDEIRS 287

Sb02g026390 227 SG----KFEES--LRVVVKCIYNPSKYYSK-LLQRSMLS--AAT--DKRMVTRAILGSDD--VGIDEIRS 283

Cs340270 227 CG----EFENA--LRTVIKCIRNPPKYFAK-VLYKSIKG---GE--SDGALKRVMLSRAE--VDLDEIQR 282

Pt13g04990 225 YM----EFEDA--LKVVMKCMCNPPTYYAK-VLYTSIKG--TTA--DNGALARVMISRAE--VDLYEIRS 281

Cp00161g00040 224 ST----EFENT--LKLVIKCMCNPLSYYAK-ELHGGIKG--RGE--RGSSLTRVMVSRAE--VDMNEIQK 280

Vv08g00710 220 SG----EFEDA--FKSVVKCMCSPAKYYAK-TLHSSIKG--SAT--DKGALAWVMASRAG--VDVDELVR 276

Gm13g26040 226 YG----QFGKA--LKVVVKCICNPAHYYAK-ILYSSIKG--ETR--DRRVLARTLVSRAE--IDIDEIRR 282

Os09g20330 230 GD----TFPGI--LRAALRCAQLPERHFAR-AVRAALER--AGA--DRRDARGRRVDAGE--RRPERVRQ 286

Cp00042g00810 163 CG----QFGKE--LRVMIRCIQNPGKFFAK-QLRMKS-----GD--GRELLIRVVVTRSG--IDIKDINN 216

Os09g23160 226 SG----NFELA--LLTILRCAENPAKYFAK-VLRKSMKG--MGT--DDSTLIRVVVTRTE--IDMQYIKA 282

Bd4g29680 226 SG----NFEVA--LLTILRCAENPAKYFAK-VLRKSMKG--LGT--DDKTLIRVVVTRTE--IDMQYIKA 282

Sb02g024090 226 SG----NFEFA--LLTILRCAENPAKYFAK-LLRKAMKG--LGT--DEKTLTRVVVTRTE--IDMQYIKA 282

Zm02g30240 226 SG----NFEFA--LLTILRCAENPAKYFAK-LLRKAMKG--LGT--DDMTLIRVVVTRTE--IDMQYIKA 282

Zm07g13390 226 SG----NFEFA--LLAILRCAENPAKYFAK-LLRKAMKG--LGT--DDKTLIRVVVTRTE--IDMQYIKA 282

Bd3g36240 209 SG----NFGFG--LLTVLRCAESPAKYFAK-VMHKAMKG--LGT--SDTTLIRVVVTRTE--IDMQYIKA 265

Zm04g13650 226 SG----NFGFG--LLTVLRCADSPARYFAK-ELHRAMKG--LGT--SDSVLIRVVVTRAE--IDMQYIKA 282

Sb07g020760 228 SG----NFGFG--LLTVLRCADSPARYFAG-VLHKAMKG--LGT--SDSTLIRVVVTRAE--IDMQYIKA 284

Os08g32970 227 SG----NFGFG--LLTILRCAESPAKYFAK-VLHEAMKG--LGT--NDTTLIRVVTTRAE--VDMQYIKA 283

Cs234810 206 SG----YFEYG--LLTIVRCAENPALYFAK-VLHKAMKG--MGT--DDSTLIRIIVTRTE--IDMQYIKT 262

Cs234800 226 SG----NFEHG--LLTILLCAENPGFYFAK-VLRKAMKG--MGT--DDSTLIRVIVSRAE--IDMQYIKA 282

Vv01g05380 226 SG----HFEFA--LLTILQSAENSGKYFAK-VLHKAMKG--LGT--DDTTLTRIIVTRAE--IDLQYIKQ 282

Gm08g06100 225 SG----NFALA--LLTIVQCAENPAKYFAK-VLRKAMKG--LGT--DDTKLIRVIVTRAE--IDLQYIKA 281

Mt8g107640 225 SG----NFAHA--LLTIVQCAESPAKYFAK-VLRKAMKG--LGT--DDTKLMRVIVTRSE--IDLHYIKA 281

Gm07g12030 227 SG----AFEHA--LLTIIQCAVNPGKYFAK-VLRKAMKG--LGT--DDSTLIRVIVTRTE--VDMQYIKA 283

Gm09g30190 227 SG----AFEHA--LLTIIQCAVNPGKYFAK-VLHKAMKG--LGT--DDSTLIRVVVTRTE--VDMQYIKA 283

Pt15g04350 226 SG----HFEHA--LKTILLCSENPANYFAK-VLHKAMKG--MGT--NDTALIRVIVTRTE--IDMHYIKA 282

Pt12g03690 226 SG----HFEHA--LKTILQCSENPAKYFVK-LLRKAMKG--LGT--NDTALIRVIVTRTE--IDMQYIKA 282

Cp00003g03400 224 SG----MFEFG--LLTILQCAHNPAKFFAK-ELYKAMKG--LGT--NDTTLIRIIVTRTE--IDMQYIAA 280

Gm07g28080 226 SG----NFESA--LLTILRCATDPAMYFAK-ILRKSMKG--VGT--DDSRLIRVIVTRTE--IDMQFIKI 282

Gm20g01460 217 SG----SFGSA--LLTILRCATDPAMYFAK-ILRKSMKG--VGT--DDSRLIRVIVTRTE--IDMHYIKI 273

Cp00002g01210 226 SG----NFEHA--LLTILQCSENPAFYFAK-VLRKAMKG--LGT--DDTTLIRIIMTRVE--VDMKYIKA 282

At1g68090 226 RG----NFEHV--LLTILQCAENSCFYFAK-ALRKSMKG--LGT--DDTALIRIVVTRAE--VDMQFIIT 282

Pt10g10090 226 SG----NFKYA--LLTILEYAVDPTKHYAT-MLRKAMKG--LGT--DDSTLIRILATRAE--IDLQKIKE 282

Pt08g13700 226 SG----NFKYA--LLTILQYAVDPTKHYAT-VLRKATKG--LGT--DDSTLIRILVTRAE--IDLQRIEE 282

Sm271856 226 SG----DFEDA--LRLIVKSVTRPGRYFAK-VLYDSMKR--MGT--DDSTLIRVVVTRAE--QDMQYIKA 282

Sm167346 226 SG----DFEDA--LRLIVKSATRPGRYFAK-VLYDSMKR--MGT--DDSTLIRVVVTRAE--QDMQYIKA 282

Sm124402 226 SG----DFEDA--LRLIVKSVTRPGRYFAK-VLYGSMKR--MGT--DDSTLIRVVVTRAE--QDMQYIKA 282

Sm94768 226 SG----DFENA--LRLIVKSATRPGRYFAR-VLYDSMKG--MGT--DDSTLIRVVVTRAE--QDMQYIKA 282

Sm227533 226 SG----DFEDA--LRLIVKSVTRPGRYFAK-VLYDSMKR--MGT--DDSTLIRVVVTRAE--QDMQYIKA 282

Pp1s219_3V6 225 SG----HFEAA--ILAVVQCTCNPAKFFAQ-ELHDAMKG--YGT--KDADLMRVITTRAE--IDMYYIKQ 281

Pp1s37_276V6 225 SG----HFEDA--LLAVVQCTCYPARYFAQ-ELYSSMKG--LGT--KDRDLIRIITTRAE--IDMYYIKQ 281

Pp1s6_292V6 226 SG----DFEQA--LRYTVMCFRQPAKFYAE-ELHTALGG--AGT--DDDALIRVITTRAE--VDMQYIKL 282

Pp1s102_141V6 215 CG----EFEQA--LRYTVMCYRQPAKFYAE-ELNAALGG--AGT--DDDALIRVVTTRAE--VDMQYIKL 271

Pp1s1_594V6 228 SG----EFEQA--LRCTVICFRQPAKFYAE-ELCNALGA--AGT--DDDALIRVVTTRAE--VDMQYIKL 284

Pp1s61_299V6 227 AG----EFLQA--LRAVVQCLRRPSEFYAE-DIITALSK-GNGPV-DEDTLVQIITTRAD--VDMHMIRI 285

Pp1s38_63V6 227 SG----EFLQA--LRAALQSLRQPSKFYAE-ELSDALSG--IGT--DEETLVLIITTRAE--VDMQFIKL 283

PsABK21977 227 SG----KFEYA--LRIIIKCICYLAKYFSK-VLRISLDQ----S--EYAALTRVMVTRAE--VDMEEIKA 281

PsACN40166 220 -----------------------------K-VLRISLDQ----S--EYAALTRVMVTRAE--VDMEEIKA 251

Cp00213g00130 225 KD----EYLQL--LRAAIKCLTCPEKYFEK-LLRLAINK--MGT--DEWALTRVITTRAE--IDMQRIKE 281

Bd3g58830 225 KD----EFQAT--LRAIIRCFTCPDRYFEK-IIRLALGG--VGT--DENSLTRIITTRAE--VDLKLIKE 281

Os02g51750 225 KD----EFLGT--LRAIIRCFTCPDRYFEK-VIRLALGG--MGT--DENSLTRIITTRAE--VDLKLIKE 281

Sb04g027590 225 KD----EFLST--LRAIIRCFTCPDRYFEK-VIRLALGG--VGT--DEDALTRVITTRAE--VDLKLIGE 281

Zm05g40790 225 KD----EFLST--LRAIIRCFTCPDRYFEK-VIRLALGG--MGT--DEDDLTRVVTTRAE--VDLKLIKE 281

Bd1g45487 226 KD----EFLKT--LRAVIRCFTCPDRYFEK-VIRLAIAG--TGT--DENSLTRIITTRAE--VDLKLIKE 282

Os06g11800 227 KD----EFLST--LRAIIRCFCCPDRYFEK-VIRLAIAG--MGT--DENSLTRIITTRAE--VDLKLITE 283

Zm06g16450 225 QDVTLFSYLSC--LYVLLHCIHLPNL-------------------------------------------- 248

Sb10g007760 225 KD----EYLKT--LRAIIRCFSCPDRYFEK-VARQAIAG--LGT--DENALTRVITTRAE--VDLKLIKE 281

Cs273000 230 ND----EYLKL--LRTTVKSLTFPERHFAK-ILRLAINK--LGT--DEWALARVVASRAE--IDMERIKE 286

At5g65020 225 DD---NDYMKL--LRAVITCLTYPEKHFEK-VLRLSINK--MGT--DEWGLTRVVTTRTE--VDMERIKE 282

Gm13g01870 225 KD----EFLSL--LRATVKCLIRPEKYFEK-VVRLAINK--RGT--DEGALTRVVATRAE--VDLKNIAD 281

Mt5g063670 225 KN----EYLSL--LRSTVKCLVFPERYFAK-IIREAINK--RGT--DEGALTRVVATRAE--IDLKIIAE 281

Vv18g03470 190 KD----EFLAI--LRATVKCLTRPEKYFEK-VLRLAINK--RGT--DEGALTRVVTTRAE--IDMKIIKE 246

Pt02g09420 225 ND----EFLAL--LRATVKCLTRPEKYFEK-VLRLAINK--RGT--NEGALTRVVATRAE--IDMKLIKD 281

Pt07g05300 225 DN----DFLKL--LRATIKCLTYPEKYFEK-LLRLSIKK--LGT--DERALTRVVTTRAE--VDMERIKE 281

Pt05g07550 225 DN----EFLKL--LRATIKCLTYPEKYFEK-LLRLAIKK--IGT--DEGALTRVVTTRAE--VDMERIKE 281

Gm05g31250 225 KD----EYLQL--LRAAIKCLTYPEKYFAK-VLRMAINK--LGT--DEGALTRVVTTRAE--VDLQRIAE 281

Gm08g14460 225 KD----EYLQL--LRAAIKCLTYPEKYFAK-VLRLAINK--LGT--DEGALTRVVTTRAE--VDLQRIAE 281

At1g35720 226 DD----KFLAL--LRSTIQCLTRPELYFVD-VLRSAINK--TGT--DEGALTRIVTTRAE--IDLKVIGE 282

At5g10230 225 EN----EYIQL--LKAVIKCLTYPEKYFEK-VLRQAINK--LGT--DEWGLTRVVTTRAE--FDMERIKE 281

At5g10220 227 ND----DYVQL--LKTAIKCLTYPEKYFEK-VLRRAINR--MGT--DEWALTRVVTTRAE--VDLERIKE 283

Cp00036g01250 199 NE----EFLAL--LMATVKCLTRPEKYFEK-VLRLAINR--QGT--DEGALTRVVATRAE--VDMKLITE 255

Cs217870 223 ND----NYLKL--LRSAIKCLTWSEKYFEK-VLRLAIKG--LGT--DEEALTRVVVTRAE--VDMKRIAE 279

Cp00042g00660 46 KD----EYLKL--LRATIKCLTYPEKYFEE-VLRLSVKG--LGI--DEEALTRVVVTRAD--VDMKHIKE 102

PsABK22223 225 PE----EFLES--LRVVIKCICFPERYFAK-VLRLAIDK--LGT--EEEALIRVVVTRAE--TDMNNIKE 281

Zm03g04200 229 DP----TGYLR-ALRAAVRCVADPSKYFAK-VLRSATRE-SAGT--DEDSLARVVLLHAE-KDDMGAICA 288

Bd2g13620 223 -------------------------------VLRNAMHE-SAGT--DEDSLTRVVVTHAE--KDLRDIKD 256

Os01g31270 198 DP----TGYSH-ALRTALRCISDANKYFVK-VLRNAMHK--SGT--NEDSLTRVIVLHAE--KDLKGIKD 255

Zm08g03950 235 DD---PTGYLR-ALRAAVRCVADPTKYFAKQVLRNATRE-AAGT--DEDSLTRVVVLHAE-KDDMGAICG 296

Sb03g004990 231 DP----TGYPR-ALRTAARCVADPSKYFAK-VLRHATRE-SAGT--DEDSLTRVVVVHAE-KDDMGAICA 290

Gm13g26960 224 ST----DFQKA--LHTAIRCINDHKKYYEK-VLRNAIKG--VGT--DEDALTRVVVSRAE--KDLRDIKE 280

Gm15g38010 224 ST----DFQKA--LHTAIRCINDHKKYYEK-VLRNALKN--VGT--DEDALTRVVVSRAE--KDLRDIKE 280

Cs308090 226 GK----EFTEA--LRTIIECIDDPYQYYEK-VVRNAIKR--VGKS-DEDALTRVVVSRAE--KDLRQIKE 283

Cs308080 226 AN----EFTEA--LKTVIRCINDPVKYYEK-VVRNAIKK--VGKS-DEDALTRVVVTRAE--KDLRQIKE 283

Gm13g26990 223 SD----EFQRA--LYTAIRAINDPIKYYEK-VVRNAIKK--VGT--DEDALTRVVVSRAE--KDLKIISE 279

Gm15g38040 223 SD----EFQRA--LYTAIRGIKDPIKYYEK-VVRNAIKK--VGT--DEDALTRVVVSRAE--KDLKIISE 279

Mt8g038210 223 SD----EFQKA--LYTTIRSFNDHVKYYEK-VVRDAIKK--VGT--DEDALTRVIVSRAQ--HDLKVISD 279

Mt8g038220 224 SD----DFHKA--LHTTIRCINDHKKYYEK-ILRGALKR--VGT--DEDGLTRVVVTRAE--KDLKDIKE 280

Gm11g21480 224 SD----DFHKV--LHTAIRCINDHKKYYEK-VLRNAVKK--FGT--DEDGLSRVIVTRAE--KDLKDIKE 280

Mt3g018780 224 SD----DFQKT--LHTAIRCINDHKKYYEK-VLRNAIKK--FGT--DEDGLSRVIVTRAE--KDLRDIKE 280

Gm04g27100 201 SD----EFHKA--ANLAISCINDHKKYYEK-VLRNAMEH--LGT--AEDALTRVIVTRAE--KDLKEIKE 257

Mt8g038180 224 SD----DFLKA--VHVAIRCIDDHKKYYEK-VLRGALKR--IGT--DEDGLTRVVITRAE--KDLKDIKE 280

Cp04842g00010 146 DN----ELAVL--LYTAIRCLVDPIKYFEK-VLRNSIKK--IGT--DEDALTRVIVTRAE--KDLQDIKD 202

Cp36671g00010 225 DN----EFAMV--LYITIRCLVDPKKYFEK-VLRNSIKK--LGT--DEDALTRVIVTRAE--VDLQDIKE 281

Pt03g19020 211 AD----EFKTV--LRTAIRCLNDHKKYYEK-ILRNAIKK--VGT--DEDALTRVIVTRAE--KDLNDIKE 267

Mt3g018920 122 SD----GFHKA--VRIAISCINDHNKYYEK-VLRNAMEI--VGI--NEDALTRVIVTRAE--KDLEDIKK 178

Mt3g018790 224 SD----GFHKA--VSLAISCINDHNKYYEK-VLRNAMET--VGT--DEDALTRVIVTRAE--KDLEDIKK 280

Cp00157g00670 224 GN----EYLAV--LRTVIRCIKNSKKYFAK-VLRNAINT--VGT--DEDALSRVIVTRAE--KDLKDIME 280

Cs138380 225 SD----EYLAA--LRTVIRCIRDPKKYYAK-VLRNAMNT--DRV--DKDGISRVIVTRAE--KDLKEIME 281

Pt01g27650 224 ID----EYLGA--LRTAVRCIRDPRKYFVK-VLRRAVHK--EDT--DEDALSRVIVTRAE--KDLKEIKE 280

At5g12380 224 TN----EYLSA--LRAAIRCIKNPTRYYAK-VLRNSINT--VGT--DEDALNRVIVTRAE--KDLTNITG 280

Vv06g10680 224 TE----EFSAA--LRAAIRCIRNPKKYLQK-LLCNVINN--MGT--DEDTLSRVIITRAE--KDLKEMKE 280

Cp00197g00010 203 HN----DLAVM--LYGAILCLIDPKEYFEW-VLHKSMQG--IGT--DEDALTRVIITRAE--KDLLYIKE 259

Gm11g21460 223 SD----EFHKA--ANLAVSCINDHKKYCQK-VLCNAMEH--VGT--DEDALTRVIVTRAE--KDLKEIKE 279

Gm13g27000 205 SD----DFYKA--VNVAIHCINDHKKYYEK-VLRNAIKG--VGN--NEDGQTRVFVTRAE--KDLKDIKE 261

Gm15g14350 224 ND----EYMTA--LRTVIRCIKNPRRYLAK-VLCYALNE--LIA--EEHELSRVIITRAE--RDLNEIND 280

Os05g31750 222 ----------EETLREAALCLATPARYFSE-VVAAAVSD--GADHHAKEALTRVAVTRAD--VDMDAIRA 276

Bd2g26760 219 ---------EETLIREAVQCLAAPEMYFSQ-VMEAALRE--GADHHGKEALARVAVTRSD--VDMDGIRA 274

Zm06g23270 ----------------------------------------------------------------------

Bd1g62130 218 GI-------C---LQEAVRCLDAPAKYFGE-VIAGAFKE--GADKQAKAALTRVVVSRSE--ADMEEIKE 272

Zm01g15800 227 ----------EPCLREAVRCLDSPPKYFSE-VIHRAFSD--DADRQAKAALTRVLVSRAD--TDMEDIKD 281

Sb01g035050 228 ----------EPCLREAVKCLDSPPRYFSE-VISRAFRD--DADRQAKAALTRVVVSRAD--TDMEDIKD 282

Mt8g038170 216 ----------DLRFKETVQCLCTPQVYFSK-VLDAALKN--DVNKNIKKSLTRVIVTRAD--IDMKEIKA 270

Cs308100 221 ----------DLRLQEAVLCLTNPVKYFTQ-LLNVSLKA--DADKKIKKVLTRIVVTRAD--NDMKEIKV 275

Gm13g27010 217 --------LR---FKEAVQCLCTPQIYFSK-VLNAALKI--DVDKNTKKSLTRVIVTRAD--IDMKDIKA 270

Gm15g38060 217 --------LR---FKEAVQCLCTPQTYFSK-VLNAALRI--DVDKNTKKSLTRVVVTRAD--IDMKDIKA 270

Pt01g06030_ 216 LI-----------LKETVECLCTPHAYFSK-VLDEAMSS--DAHKNTKKGLTRVIVTRAD--VDMKEIKE 269

Vv00g25070 217 ----------ELSLDETMQCLCTPQTYFSK-VLGAAFQN--DADEHAKEALTRVIVTRAD--DDMKEIKE 271

At2g38750 222 ----------SSLLNEALICLLKPALYFSK-ILDASLNKDADKT--TKKWLTRVFVTRADHSDEMNEIKE 278

Zm08g13570 231 SD----QLAAM--LKTAVWCLTSPEKHFAE-VIRRSIVG--LGT--DEESLTRVIVSRAE--IDMKKVKE 287

Bd2g26770 231 ND----QLSAM--LKTAVWCLTTPEKHFAE-VIRNSIVG--LGT--DEESLTRGIVSRAE--IDMKKVKE 287

Zm06g23280 233 SD----QLAAV--LKTAVWCLTSPEKHFAE-VIRSSIVG--LGT--DEESLTRAIVSRAE--IDMKKVKE 289

Sb09g018980 232 SD----QLAAV--LKTAVWCLTSPEKHFAE-VIRSSIVG--LGT--DEESLTRAIVSRAE--IDMKKVKE 288

Os05g31760 233 GD----QLAAV--LKAALWCLTSPEKHFAE-VIRTSILG--LGT--DEEMLTRGIVSRAE--VDMEKVKE 289

Sb02g041850 205 CC---GQLAGM--LRSAVLCLASPEKHFAE-VIRYSILG--LGT--YEDMLTRVIVSRAE--VDMEQIKE 262

Bd1g62120 105 SS---SQFARM--LKIAVWCLTSPEKHFAE-VIRYSILG--IGT--DEDALTRAIVSRAE--IDMEKIKQ 162

Sb01g035040 155 SS---SQFAKI--LRSAVWCLTSPEKHFAE-AIRYSILG--FGT--DEDTLTRAIISGSE--IGMNKIKE 212

Bd1g18990 204 CS---SQFAGA--LKSAVWCLTSPEKHFAE-VIRNAVEG--LGT--YEDVLTRAVVSRAE--VDMASVRA 261

Os07g46550 167 CI---GQFGRM--LKTAVWCLTSPEKHFAE-VIRHSILG--LGT--YEDMLTRVIVSRAE--IDMRHIRE 224

Pt01g06020 228 KG----DLESL--LRVVIKCIDTPEKHFAE-VIGEAIIG--FGT--DEDSLTRAIVARAE--IDTMKIRG 284

Gm15g38070 227 NG----DLESL--LHTVIWCIDCPEKHFAK-VVRDSIVG--FGT--DEDSLNRAIVTRAE--IDLLNVRF 283

Gm13g27020 227 NG----DLESL--LHMVIWCIDCPEKHFAK-VVRDSIVG--FGT--DEDSLNRAIVTRAE--IDLLKVRF 283

Mt8g038150 228 KG----DLTSL--LNVVVWCIECPEKHFAK-VIRDSIVG--LGT--DEDSLNRAIVTRAE--IDLLKVRF 284

Vv00g25060 228 IG----DLASL--LRVIIWCIDSPEKHFAE-VIRASIVG--LGT--DEDSLTRAIVTRAE--IDMMRSEG 284

Cp00197g00020 228 NG----DLESL--LRMVVSCIDCPEKHFAE-VIGTSVIG--FGT--DEDSLTRAIVSRAE--IDMMKIRG 284

At2g38760 230 GD---ADLRSL--LKVAIFCIDTPEKHFAK-VVRDSIEG--FGT--DEDSLTRAIVTRAE--IDLMKVRG 287

Vv00g00800 228 SD----DLGSL--LRVVILCIDAPEKHFAE-VIRASLSG--HRT--DVHSLARAILARVE--IDMMKIKE 284

Vv03g02080 228 NG----DLGSI--LRGVILCIVSPEKHFAE-VIKASTVG--YWTK-DEDSLTRAIVTRAE--IDMTKIKG 285

Vv00g00650 228 NG----DLGSI--LRGVILCIVSPEKHFVE-VIRASTIG--YWTK-DEDSLTRAIVTRAE--IDMTKIKE 285

Vv00g00720 85 NG----DLGSI--LRGVIWCIVSPEKHFAE-VIKASTVG--YWTK-DEDSLTRAIVTWAE--IDMTKIKG 142

Vv00g00760 117 NG----DLGSI--LRGVILCIVSPEKHFAE-VIRASTVG--YWTK-DEDSLTRAIVTQAE--IDMTKIKG 174

Vv00g00750 218 SD----DLGSL--LRVVILCIDAPEKHFAE-VVEI----------------------------------- 245

Vv00g00710 228 SD----DLGSL--LRVVILCIDAPEKHFAE-VIRASLSG--HRT--DVHSLARAILARVE--IDMMKIKE 284

Vv00g00660 228 SD----DLGSL--LRVVILCIDAPEKHFAE-VVEI----------------------------------- 255

Ot24272 221 SG----DYED-----ALVACISPKERTIAR-GMRQCIAG--WFSS-TNKTGIMALLTHKD--MVMPRLRK 275

MRCC299_03g01870 226 TG----LFESEGFRNTLMGLLTNREEQIAI-YLKEAFEG--WFSN-DDWGLISMLVHRTP--QEMELIRN 285

Cs307980 188 -------------------------------------KI--GVT--PKDSLSRIIITCPK--VDLDKIKI 214

Cs307970 234 --------------STTFLCLVDPIEYFYQ-VLSNSIDS--SPSLHCLDSISRIIMTRRG--VDLDEINT 284

[Consensus_aa:](http://prodata.swmed.edu/promals3d/info/consensus.html) ss....p*@*.....**L**.*hhl*.**C***h*..s.c*@@*.c.*hl*p.t*h*.....ss..scps**L***h***R***hll*o**R***h***-**...**D***h*p.**I+**.

[Consensus_ss:](http://prodata.swmed.edu/promals3d/info/consensus_ss.html) hhhhh hhhhhhhhh hhhhhhh hhhhhhh hhhhhhhhhhh h hhhhhhhh

Conservation: 7 7 6 5 5 7

Bd4g31920 281 AFKSNFG-RNLGDFIHESLPQSDYRDFLW------MWQGG---Q--- 314

Os09g27990 283 VFKSSYG-KDLEDFILESLPENDYRDFLL------GAAKGSRAS--- 319

Zm02g31380 288 AFQSSYG-KSLAEYIQENLPGSDYRDFLV------AVASASVAQ--- 324

Sb02g026390 284 AFKSSYG-RNLADYIQENLPESDYKDFLV------AVARGSVAP--- 320

Cs340270 283 AFKGRYG-VQLTDAICERTSCDDYRDFFV------ALATKKAQ---- 318

Pt13g04990 282 IFKRKYG-MELKDAICERIPSGDYRDFLA------AIASTTTII--- 318

Cp00161g00040 281 VFKKKYG-VELRDSICESLPSGDYTDFLL------ALASKTLCS--- 317

Vv08g00710 277 VFRKKYG-MELKEAIYGSIPSGDLRD--------------------- 301

Gm13g26040 283 VFKEKYG-KELADAICEGFPSGDYYRDFL------VALATRSK---- 318

Os09g20330 287 RRHREVG-RRLDRRLARGV----------------AQVGLRTRD--- 313

Cp00042g00810 217 AFTAKTG-SSLENLVRREFTNGIVAAILT------GLIRG------- 249

Os09g23160 283 EYYKKYK-KSLAEAIHSET-SGNYRTFLL------SLVG-SH----- 315

Bd4g29680 283 EYYKKYK-KPLGDAIHSET-SGGYRTFLL------SLVGGH------ 315

Sb02g024090 283 EYFKKYK-KPLAEAINSET-SGNYRAFLL------SLVGHGH----- 316

Zm02g30240 283 EYLKKYK-KPLAEAINSET-SGNYRTFLL------SLVGHGH----- 316

Zm07g13390 283 EYFKKYK-KPLAEAIHSET-SGNYRTFLL------SLVGPGH----- 316

Bd3g36240 266 EYHKKYK-RSLADAIHSET-SGNYRTFLL------SLVGRDR----- 299

Zm04g13650 283 EYHSMYK-RSLADAIHAET-SGNYRTFLL------SLVGRDRT---- 317

Sb07g020760 285 EYHRMYK-RSLADAIHAET-SGNYRTFLL------SLVGRDRTY--- 320

Os08g32970 284 EYHRSYK-RSLADAVHSET-SGNYRTFLL------SLIGRDR----- 317

Cs234810 263 EYQKKYK-KTLHDAVHSET-SGSYRDFLL------SLLG-------- 293

Cs234800 283 EYHKKYK-KTLNKAVQSET-SGSYKDFLL------SLLGPDR----- 316

Vv01g05380 283 EYRKKYG-KTLNDAVHSET-SGHYKAFLL------ALLGPNH----- 316

Gm08g06100 282 EYLKKYK-KTLNDAVHSET-SGHYRAFLL------SLLGPNQ----- 315

Mt8g107640 282 EYLKKYK-KTLNDAVHSET-SGHYRAFLL------SLLGPNQ----- 315

Gm07g12030 284 AYLKKHK-KTLNDEVHSET-SGHYRTFLL------SLLGPNH----- 317

Gm09g30190 284 AYLKKHK-KTLNDEVHSET-SSHYRTFLL------SLLGPNH----- 317

Pt15g04350 283 EYLKKYK-KTLNDAVHSET-SGNYQAFLL------ALLGPNH----- 316

Pt12g03690 283 EYLKKYR-KTLNDAVHSET-SGHYRAFLL------ALLGPNQ----- 316

Cp00003g03400 281 EYFKKYK-ETLNEAVHSET-SGHYRTFLL------YLLGENH----- 314

Gm07g28080 283 AYYKKYG-KPLTHAVKSDT-SGHYKDLLL------NLLGSDY----- 316

Gm20g01460 274 TYYKKYG-KPLTHAVKSDT-SGHYKDFLL------NLLGSDY----- 307

Cp00002g01210 283 EYRKKYG-KTLNDAVHSDT-SGHYRTFLL------SLLGPNH----- 316

At1g68090 283 EYRKRYK-KTLYNAVHSDT-TSHYRTFLL------SLLGPNV----- 316

Pt10g10090 283 DYLKRYK-RPLVEVVHSDT-SGYYRAFLL------SLLGSKF----- 316

Pt08g13700 283 EFLKKYK-RPLPEVVHSET-SGHYRAFLL------SLLGSKY----- 316

Sm271856 283 DFYQKYK-KPLESMISGDT-SGNYKHFLL------SLVGGH------ 315

Sm167346 283 DFYQKYK-KPLESMISGDT-SGNYKHFLL------SLVGGH------ 315

Sm124402 283 DFYQKYK-KPLESMISGDT-SGNYRHFLL------SLVGGH------ 315

Sm94768 283 DFYQKYK-KPLESMISVDT-SGNYKHFLL------SLIGGH------ 315

Sm227533 283 DFYQKYK-KPLESMISGDT-SGNYRHFLL------SLVGGH------ 315

Pp1s219_3V6 282 EFQAMFK-KTLQEAIQSNT-SGDYRHFLL------SLVGDA------ 314

Pp1s37_276V6 282 EFQIMYG-TTLEYMIAGDT-SGDYRYFLL------SLVGGA------ 314

Pp1s6_292V6 283 EFANECK-RSLEEMIANDT-IGNYRYFLL------TLVGPGDLG--- 318

Pp1s102_141V6 272 EFANESK-KKLEDMIANET-SGNYRYFLL------TLVGPGDLG--- 307

Pp1s1_594V6 285 EFTNLSK-RTLEEMVANDT-AGTYRYFLL------TLVGPGDLG--- 320

Pp1s61_299V6 286 EFMKECK-RALEQVISERA-MGVIGQFLV------TAIRQRDMV--- 321

Pp1s38_63V6 284 EFMNECK-RSLEDVVRDET-IGKLRQLLL------TILGQGDML--- 319

PsABK21977 282 TYREKYG-ISLEQAICKQT-SGSYRDFLL------QLACWEATSQPK 320

PsACN40166 252 TYREKYG-ISLEQAICKQT-SGSYRDFLL------QLACWEATSQPK 290

Cp00213g00130 282 EYQRRNS-VPLDRAIAKDT-TGNYEKMLL------ALIGHGDA---- 316

Bd3g58830 282 AYQKRNS-VPLEKAVSKDT-TRDYEDMLL------ALLGAEY----- 315

Os02g51750 282 AYQKRNS-VPLERAVAKDT-TRDYEDILL------ALLGAE------ 314

Sb04g027590 282 AYQKRNS-VPLDRAVAKDT-TRDYEDILL------ALLGAE------ 314

Zm05g40790 282 AYQKRNS-VPLERAVAKDT-TRDYEDIML------ALLGAE------ 314

Bd1g45487 283 AYQKRNS-VPLERAVAGDT-SGDYESMLL------ALLGKE------ 315

Os06g11800 284 AYQKRNS-VPLERAVAGDT-SGDYERMLL------ALLGQEQ----- 317

Zm06g16450 -----------------------------------------------

Sb10g007760 282 AYQKRNS-VPLERAVAGDT-SGDYESMLL------ALLGQE------ 314

Cs273000 287 EYYRRNS-VPLGRAIAKDT-SGDYEKMLL------ELIGHSDA---- 321

At5g65020 283 EYQRRNS-IPLDRAIAKDT-SGDYEDMLV------ALLGHGDA---- 317

Gm13g01870 282 EYQRRSS-VPLERAIVKDT-TGDYEKMLV------ALLGHDDA---- 316

Mt5g063670 282 EYQRRNS-IPLDRAIVKDT-TGDYEKMLL------AILGHNDA---- 316

Vv18g03470 247 EYHKRNS-VTLDHAIGKDT-TGDYEKMLL------ALIGHGDA---- 281

Pt02g09420 282 EYQRRNS-IPLDRAIVKDT-DGEYEKLLL------ELVGHEDA---- 316

Pt07g05300 282 EYHRRNS-VTLERDIAGDT-SGDYERMLL------ALIGHGDA---- 316

Pt05g07550 282 EYHRRNS-VTLDHDIAGEA-SGDYERMLL------ALIGHGDA---- 316

Gm05g31250 282 EYQRRNS-IPLDRAIANDT-SGDYQSILL------ALVGHED----- 315

Gm08g14460 282 EYQRRNS-IPLDRAIASDT-SGDYQSILL------ALVGHED----- 315

At1g35720 283 EYQRRNS-IPLEKAITKDT-RGDYEKMLV------ALLGEDDA---- 317

At5g10230 282 EYIRRNS-VPLDRAIAKDT-HGDYEDILL------ALLGHDHA---- 316

At5g10220 284 EYLRRNS-VPLDRAIANDT-SGDYKDMLL------ALLGHDHA---- 318

Cp00036g01250 256 EYQQRSS-VPLDRAITKDT-HGDYEKMLL------ELIVHDDA---- 290

Cs217870 280 EYYRRNS-VPLGQAIKGDT-SGDYESMLL------ALIGKESN---- 314

Cp00042g00660 103 EYYRRNS-EPLDAAIKGDN-SGNYERMLL------ALIGHGDT---- 137

PsABK22223 282 EYHKRTS-KTLEHAIAADT-SGYYEEFLL------TLIGKEQS---- 316

Zm03g04200 289 AFLKRAS-CTLEQAVAKET-SGDYRSFLL------ALLGS------- 320

Bd2g13620 257 VFRKTTS-VALEQAIAKET-SGDYKTFIV------ALVGSQ------ 289

Os01g31270 256 AFQKRAS-VALEKAIGNDT-SGDYKSFLM------ALLGSGI----- 289

Zm08g03950 297 AFQKRAS-CTLQQAIAKET-SGDYSSFLL------ALLGS------- 328

Sb03g004990 291 AFQKRAS-CTLEQAIAKET-SGDYRSFLL------ALLGS------- 322

Gm13g26960 281 LYYKRNS-VHLEDAVAKEI-SGDYKKFIL------TLLGKED----- 314

Gm15g38010 281 RYYKRNS-VHLEDAVAKEI-SGDYKKFIL------TLLGKED----- 314

Cs308090 284 AYHKRNS-VTLDDAVSKET-SGDYKRFIL------ALLGN------- 315

Cs308080 284 AYHKRNS-VTLDDAVKKET-SGDYERFIL------ALLGNQTE---- 318

Gm13g26990 280 VYYKRNS-VLLEHAIAKEI-SGDYKKFLL------TLLGKED----- 313

Gm15g38040 280 VYYKRNS-VLLEHAIAKET-SGDYKKFLL------TLLGKED----- 313

Mt8g038210 280 VYYKRNS-VLLEHVVAKET-SGDYKKFLL------TLLGKEE----- 313

Mt8g038220 281 LYYKRNS-VHLEDAVAKEI-SGDYKKFIL------TLLGKQD----- 314

Gm11g21480 281 LYYKRNS-VHLEDEVSKET-SGDYKKFLL------TLLGK------- 312

Mt3g018780 281 LYYKRNS-VHLEDEVSKET-SGDYKKFIL------TLLGKHD----- 314

Gm04g27100 258 VYYKRNS-VHLEHAVAKET-SGDYKKFLL------SLMGKEE----- 291

Mt8g038180 281 LYYKRNS-VHLEDTVAKEI-SGDYKKFLL------TLLGKGH----- 314

Cp04842g00010 203 LYYKKNS-ELLEHAVAKDT-TGDYKHFLL------TLLGKED----- 236

Cp36671g00010 282 LYYKKNS-ELLEHAVAKDT-SGDYKHFLL------TLLGKED----- 315

Pt03g19020 268 IYYKRNS-VPLDQAVANDT-SGDYKAFLL------ALLGKEE----- 301

Mt3g018920 179 VYYKRNS-VQLEHAVAKKT-SGDYKKFLL------TLMGKEE----- 212

Mt3g018790 281 VYYKRNS-VQLEHAVAKKT-SGDYKNFLR------TLMGKEE----- 314

Cp00157g00670 281 LYLKRNN-ISLEQAVARDT-SGDYKAFLL------ALLGSEEN---- 315

Cs138380 282 MYLKRNN-ISLEEAVSREI-GGDYKAFLL------ALLGIDQPL--- 317

Pt01g27650 281 LYLKRNN-ISLDQAVAVDT-HGEYKEFLL------TLLGNEKN---- 315

At5g12380 281 LYFKRNN-VSLDQAIAKET-SGDYKAFLL------ALLGHGKQL--- 316

Vv06g10680 281 LYLERNS-RSLEDAVSSET-TGDYKAFLL------TLLGNQEF---- 315

Cp00197g00010 260 LYHKKNG-KLLAHDVAGDT-SRHYKHFLL------TLLGDEK----- 293

Gm11g21460 280 MYYKRNI-VHLEHVAAKET--S------------------------- 298

Gm13g27000 262 LYYKKNS-VHLEDTMAKEN-SGYYKKFLL------TLLGKGG----- 295

Gm15g14350 281 LYFKRNG-VTLDSSVAKKT-SGNYKNFLL------ALLGNN------ 313

Os05g31750 277 AYHEQFG-GRLEDAVAGKA-HGYYRDALL------SLVAGGK----- 310

Bd2g26760 275 AYQEQFG-ARLEDAVAACA-HGHFKDALL------SLIAGGK----- 308

Zm06g23270 -----------------------------------------------

Bd1g62130 273 AYVKQHG-AKLVDAVAKNT-HGHYRDALL------AMIGK------- 304

Zm01g15800 282 AYTRQYG-TKLADAVAKNT-HGHYKEALL------AIIGK------- 313

Sb01g035050 283 AYARQYG-AKLADAVAKNT-HGHYKDALL------AIIGK------- 314

Mt8g038170 271 EYNNLYG-VSLPQKIEETA-KGNYKDFLL------TLIARGG----- 304

Cs308100 276 EFKKQFG-ISLAEKIGSVC-NGSYKDFLI------TLLARSD----- 309

Gm13g27010 271 DYHNLYG-VSLPQKVEEVA-RGSYKDFLL------NLIVRGG----- 304

Gm15g38060 271 EYHNLYG-VSLPQKVEEVA-RGSYKDFLL------NLIVRGG----- 304

Pt01g06030_ 270 EYMNLFG-VSLSKKIEEKA-NGNYRDFLV------TLITRDN----- 303

Vv00g25070 272 EYQKKYG-VSLSKKIEDAV-NGNYKDFLL------TLIARGE----- 305

At2g38750 279 EYNNLYG-ETLAQRIQEKI-KGNYRDFLL------TLLSKSD----- 312

Zm08g13570 288 EYKVRYR-TTVTSDVNGDT-SGYYNSILL------TLVGRE------ 320

Bd2g26770 288 EYKARFK-TTVTNDIIGDT-SGYYKDILL------TLVGPE------ 320

Zm06g23280 290 EYRARYR-TTVTSDVNGDT-SGYYNVILL------TLVGPE------ 322

Sb09g018980 289 EYKARYR-KTVTSDVNGDT-SGYYNGILL------TLVGPE------ 321

Os05g31760 290 EYKVRYN-TTVTADVRGDT-SGYYMNTLL------TLVGPEK----- 323

Sb02g041850 263 EYRARYG-SAVSLDVAGDT-SFGYRDMLL------ALLGGQE----- 296

Bd1g62120 163 EYKVRLK-STVTNDVIGDT-SGYYMDILL------ALVGNED----- 196

Sb01g035040 213 EYKVRFK-TTVTSDVVGDT-SGYYKDFLL------TLVGSED----- 246

Bd1g18990 262 EYRARFG-VTVASDIADDT-SFGYRDVLL------ALVGIEEGEE-- 298

Os07g46550 225 EYKVRYK-TTVTRDVVGDT-SFGYKGFLL------ALVGRED----- 258

Pt01g06020 285 EYFNIFK-TNLDGAVTGDT-SGDYKDFLM------TLLGARI----- 318

Gm15g38070 284 EYANVYK-SSLDDDVIGDT-SGYYKDFLM------TLLGKGPDGE-- 320

Gm13g27020 284 EYANVYK-TSLDDDVIGDT-SGNYRDFLM------TLLGKGSEGE-- 320

Mt8g038150 285 EYANMYK-SSLDDDVIGDT-SGDYMEFLL------TLLGKGPKGY-- 321

Vv00g25060 285 ----IFQ-HKQDQP--GQC--S------------------------- 297

Cp00197g00020 285 EYFNLYK-TNLDGAVIDDT-SGDYRDFLM------TLLGARI----- 318

At2g38760 288 EYFNMYN-TSMDNAITGDI-SGDYKDFII------TLLGSKI----- 321

Vv00g00800 285 EYFNMNK-VSLDDAVVGKT-SGGYKDFLM------TLIGARI----- 318

Vv03g02080 286 EYFKMNN-TNLDDVVRRDT-SGVYKSFLM------ALIGAKI----- 319

Vv00g00650 286 EYFKMNN-TNLDDVVRRDA-SGVYKSFLM------ALIGEKI----- 319

Vv00g00720 143 DYFKMNN-TNLDDVVRHDA-LGVYKSFLM------ALIGAKI----- 176

Vv00g00760 175 EYFKMNN-TSLDDVVRRDA-SGVYKSFLM------ALIGAKI----- 208

Vv00g00750 246 -----------------------YK---------------------- 247

Vv00g00710 285 EYFNMNK-VSLDDAVVRKT-SGGYKDFLT------TLIGAIRPL--- 320

Vv00g00660 256 -----------------------YK---------------------- 257

Ot24272 276 EFEKEFRGQTLQNCIKKEC-AGEFEAALV------SLAAYTPPK--- 312

MRCC299_03g01870 286 AYTRVHG-RDLIADIRKNC-KGDYEKALV------ALVAPRART--- 321

Cs307980 215 KFKETSK-ITLQERIRLVC-KGSYKDLLL------GVLSK------- 246

Cs307970 285 KFRMFDE-LSLQDRIKLYC-KGTYQKLLLELLLNVNILGSEAESS-- 327

[Consensus_aa:](http://prodata.swmed.edu/promals3d/info/consensus.html) .*@*.p......**L**.p.*l*..p*h*.p**G**p**Y**cp*h***L***l*......s**L***l*t........

[Consensus_ss:](http://prodata.swmed.edu/promals3d/info/consensus_ss.html) hhhhhh hhhhhhhh hhhhhhhh hhh
