# Supplementary material for: Genome-wide Comparative Analysis of Annexin Superfamily in Plants
Source: PLoS One. 2012 Nov 2;7(11):e47801. doi: 10.1371/journal.pone.0047801 (PMC3487801; doi:10.1371/journal.pone.0047801)
Supplement: Figure S2 — A–D Alignments used to detect calcium binding sites and also to build the sequence logos for the four annexin domains. Taxon-specific indels are removed to optimize the alignments. (DOC) [file pone.0047801.s002.doc]

**Figure S2A**

Bd4g31920 ----SPFENDCREIHGMCD----EPCRLSRLLAHRSSSERQQIKVTYRAMFGEDLVGRLRNTLLPDQDNLLY

Os09g27990 VT--TGFEDECREIHDACN----QPRRLSVLLAHRSPSERQKIKATYRTVFGEDLAGEVQKILMVNQEKLLY

Zm02g31380 ----AGSEEACREIRRTCG----APRRLGLLLAPRSPAERQQIRAAYRARFGEDLAATLHGTLAAP-NKLLY

Sb02g026390 ---EEEEEEACREIRGASG----TPRRLGLLLAPRSPAERQQIRAAYRATFGEDLAATLHGN---QDDKLLY

Cs340270 HNMSINDLGIENDCRDIHDSWDQRSNVMVRVLAGRNAMERQQTRRIYKEIYGEDLVDRLGTIDVEPINRALS

Pt13g04990 TSSSHGFENECKEIHDSWG----RLNHLVRSLAGRSKLERQQIRETYKAMYGEDMAILLQKMQFQNGSAALY

Cp00161g00040 PSSSHGFENECREIHECWG----RTNRLIRALANRTRVERKQIRETYKTMYGEDLASFLFRRSNSI-NAALL

Vv08g00710 PSSHGGLYIYATALADQTEG---HSSQLVQPLGGRARLELGYIQEAFMELVGEVPINRFQNGYMITQRNEPG

Gm13g26040 TMTNLNFELDC---KRTHDSLGSLS-QLITSLACVTHHERQQLRETFKAVNGEDLISHLQRYEDAFYSSAIS

Os09g20330 F--AKRYEADCRHLNQFFSGNVNNARPVLEIFTARSSQEMKQICRAYSSMYRQDLIQLLSQQLSGSIAIRVA

Cp00042g00810 ---------------------------------------------------------MATNQGNSPFARVAY

Os09g23160 PPAPTNPRQDAIDLHKAFKGFGCDSTTVINILTHRDSMQRALIQQEYRTMYSEDLSRRISSELSGHHKKAML

Bd4g29680 PPGPPNPRQDAIELHKAFKGFGCDSTAVTNILGHRDSMQRGYIQHEYKTMYSEELSRRISSELSGNHKKAMS

Sb02g024090 PPAPPNPRQDAIDLHKAFKGFGCDSTAVINILTHRDSVQRGLIQQEYRAMYHEELFHRISSELSGNHKKAMS

Zm02g30240 PPAPPNPRQDAIDLHKAFKGFGCDSTTVINILTHRDSVQRGLIQQEYRAMYHEELSHRISSELSGNHKKAMS

Zm07g13390 PPAPPNPRQDAIDLQKAFKGFGCDSTTVINILTHRDSVQRGLIQQEYRAMYHEELSHRISSELNGNHKKAML

Bd3g36240 PPVLTSPRQDAAALHKAFKGFGCDSTTVINILAHRNATQRALIMQEYRAIYHQDLYHRLSTELTGNHKKAML

Zm04g13650 PPVPAWPRQDAIDLHKAFRGFGCDSTTVTNILAHRDATQRSLIQQEYRAVFNQDLARRIASELSGHHKRAML

Sb07g020760 PPVPTWPRQDAIDLHKAFRGFGCDSTTVISILAHRDAAQRAAIAQEYRAVFNQDLARRLASELSGNHKRAML

Os08g32970 PPVPTDPRRDAIDLHRAFKGFGCDATAVTAILAHRDASQRALIRRHYAAVYHQDLLHRLAAELSGHHKRAVL

Cs234810 --------------MCVYIGFGCDNGAVVNILAHRDAAQRSLIQREYKAMYHKDLIKHLKSELSGNLEKAIL

Cs234800 PPLLTSPRDDAALLYRAFKGFGCDTAAVINVLAHRDAAQRALIQQEYRAIYSEELTKRLKSELSGKLEDAIL

Vv01g05380 PPVLTSPRDDAIQLYRAFKGLGCDTAAVVHILAHRDVTQRGLIQQEYRAMYSEDLVKRLSSELSGNVKRAVL

Gm08g06100 PPLPPSPRDDAIQLYAAFKGFGCDTSVVINILAHRDATQRAYIQQEYKAMYSGDLLKRLSSELSGKLETALL

Mt8g107640 PPIPPSPRDDAMQLHRAFKGFGCDTSAVINILAHRDATQRAYLQQEYRATYSEDLLKRLSSELSGKFENAIL

Gm07g12030 PPVPPSPRDDAMQLYRAFKGFGCDTSAVINILAHRDATQRAYIQQEYRSMYSEELSKRLASELSGKLETAVL

Gm09g30190 PPVPPSPRDDAMQLYRAFKGFGCDTSAVINILAHRDATQRAYIQQEYKAMYSEELSKRLASELSGKLETAVL

Pt15g04350 PPVLSSPRDDAMQLFRAFKGLGTDTSAVINILAHRDAAQRSLIQHEYRTLYSEDLFKRLSSELTGNLETAVL

Pt12g03690 PPLLSSPRDDAMHLYRAFKGFGTDTSAVISILAHRDAAQRALIQHEYRALYAEDLLKRLTSELTGKLETAVL

Cp00003g03400 PPFPTNPKDDAIQLYRAFKGLGCDTPVVINILAHRDATQRALIQQEYRLMYSEDLLKRLASELHGKLERAVL

Gm07g28080 PPVIPSPREDAIKLHKAFKGLGCDTSKVIKILAHRNAEQRSLIQQEFETNYSELLSKRLSKELRGHVKKAVL

Gm20g01460 P----------VSKHHAFSSLGCDTSKVIKILAHRNAEQRSLIQQEFETNYSELLSKRLSKELRGHVKKAML

Cp00002g01210 PANSPSPRDDAMQLYQAFKGRGCETSVIINILAHRNATQRGLIEQEYETKYSEELSKRLYSELHGHLKKAVV

At1g68090 PMTVPSPRVDADQLFKAFKGRGCDTSVIINILAHRNATQRALIEQEYETKFSDDLRKRLHSELHGHLKKAVL

Pt10g10090 PSMQTSSRDDAVQLNRAFKGLGCDTAVVVNVLGNRNASQRDSIQQEYETLFSDDLKKQLALELHGHLKKAVL

Pt08g13700 PSMQKSSRDDAEQLNRAFKGLGCDAAVVVNILALRNASQRDSIQQEYETLFSDDLKKQLAHELHGHLKKAVL

Sm271856 PPMLPPVQQDCQALHHAFKGFGCDEKHVIQILAHRNYLQRRELVNAYRSMYGEDLLRRLEKELHGNLEQAVL

Sm167346 PPMLPPVQQDCQALHHAFKGFGCDEKHVIQILAHRNYLQRRELVNAYRSMYGEDLLRRLEKELHGNLEQAVL

Sm124402 PPMLPPVQQDCQALHHAFKGFGCNEKHVIQILAHRNYLQRRELVNAYRSMYGEDLLRRLEKELHGKLEQAVL

Sm94768 PPMPPPVQQDCQALHHAFKGFGCDEKHVIQILAHRNYLQRRELVNAYRSMYGEDLLRRLEKELHGNLEQAVL

Sm227533 PPMLPPVQQDCQALHHAFKGFGCNEKHVIQILAHRNYLQRRELVNAYRSMYGEDLLRRLEKELHGKLEQAVL

Pp1s219_3V6 PS-YLNMGEDVRELHRAFKGFGCDEKKVIQILAHRTQSQRLAIADAYHHQYGESIHKRLKSELHGKLEEVML

Pp1s37_276V6 PP-YLSMSDDVHALHRAFRGFGCDEKRVIQILAHRTQPQRDAIADAYQRQYGESIHKRLKSELHGKLEKAVL

Pp1s6_292V6 PP-YFNLQEDCKDLRSSFKGLGCNEKRVIEILARRTQAQRLEIAQAYQTVYGESLHKRLKSAFSGKLEKCIL

Pp1s102_141V6 PP-CFNLQEDCKELRSSLKGLGSNEKKVIEILGRRTQAQRLEIAQAYQTVYGESLHKRLKSAFSGKLEKCIL

Pp1s1_594V6 PP-YFNLQEDCKELRLSFKGLGCNEKRVIEILGRRTQSQRLEIAQAYQTVYGESLHKRLKAAFNGKLEKCIL

Pp1s61_299V6 FQQYPGLEDDIRDLRYALSGLFPNERKVVEILGKRSQAHRESIAEGYKLLFAESLPKRLKASMSCKAERCLM

Pp1s38_63V6 QQSYPNLHEDCKDLRNALRGISSNEKKVIEILGQRNQSQRDSLSEAYKLVFGEDLRKRLKSSISGKLEKCLT

Ot11g03220 AGCDGVGESYAKVINSAVSGLGTDTSAIIRLMVTATPEQLDATREAYSRIYKKDLIKAVGSEVSGDFKRIIT

MRCC299_56760 ALLTEQFDFEAQVLHRAMKGWGCDEDTLTTILCTLDEADIFKLQNAYSSRFEKSLEQAMLSETEGKYKRVLL

PsABK21977 SCSSTQDIKDCEAVYNCCKGIAASKGRLEHILASRNATERKELGDLFYALYKEDLSTLLHAELWGNLEKAVV

PsACN40166 SCSSTQDIKDCEAVYNCCKGIAASKGRLEHILASRNATERKELGDLFYALYKEDLSTLLHAELWGNLEKAVV

Cp00213g00130 PANVPSPAEDADQLHKAFQGWGTNEGLIISILAHRNAAQRNLIRQTYAETYGEDLLKALDKELSSDFERAVL

Bd3g58830 PAAVPPVAEDCEQLRKAFQGWGTNERLIISILAHRDAAQRRAIRAAYAEQYGEELLRALGDEIHGKFERAVI

Os02g51750 PAAVPPVAEDCEQLRKAFKGWGTNEKLIISILAHRDAAQRRAIRRAYAEAYGEELLRALNDEIHGKFERAVI

Sb04g027590 PSSVPAVAEDAEQLHKAFEGWGTNEKLIISILAHRNAAQRRAIRRAYAEAYGKELLRALGDEIHGKFERTVI

Zm05g40790 PSSVPAVAEDCEQLHKAFEGWGTNEKLIISILAHRNAAQRRAIRRGYAEAYGKELLRALGDEIHGKFERAVI

Bd1g45487 PSSVPAITDDCEQLRKAFQGWGTNEALIISILGHRDAAQRRAIRRAYAETYGEELLRSITDEISGDFERAVI

Sb10g007760 PATVPPVADDCEQLRKAFQGWGTNEALIISILGHRDAAQRRAIRRAYAEAHGEELLRSITDEISGDFERAVI

Os06g11800 PSAVPPVADDCDQLRKAFQGWGTNEALIISILAHRDAAQRRAIRRAYADTYGEELLRSITDEISGDFERAVI

Zm06g16450 PATVPPVADDCDQLRKAFQGWGTNEALIISILGHRDAAQRRAIRRAYAEAYGEELLRSITDEISGDFERAVI

Cs273000 PDHLPSPAEDCEQLRKAFQGWGTNEDLIISILAHRNAAQRSLIRKAYAETYGEDLLKALDKELSSDFERIVL

At5g65020 PSNVPLPEDDAEQLHKAFSGWGTNEKLIISILAHRNAAQRSLIRSVYAATYNEDLLKALDKELSSDFERAVM

Gm13g01870 PQPLPPVADDCEQLRKAFSGWGTNEELIVSILAHRNAAQRKLIRETYAQTYGEDLLKALDKELTSDFERLVH

Mt5g063670 PHPLPPVSDDVEQLRKAFSGWGTNENLIISILGHRNEVQRKVIREAYAKTYEEDLIKALNKELTSDFERLVH

Vv18g03470 -------TEDCEQLRKAFAGWGTNEGLIISILAHRNAAQIKSIRQTYAQTYGEDLLKDLNKELSNDFERVVL

Pt02g09420 PQQVPPVSEDVEQLRKAFSGWGTNEGLIISILGHRNAAQRKLIRQAYAEAYGEDLLKALDKELSNDFERVLL

Pt07g05300 PASVPPPYEDAEQLHKAFEGWGTNEGLIISILAHRNAAQRNLIRKVYAEAYGQDLLKDLDKELSSDFERAVL

Pt05g07550 PASVPPPYEDAEQLNKAFKGWGTNEGLIMSILAHRNAAQRNLIRQVYAEAYGQDLLKDLDKELSSDFERVVL

Gm05g31250 PAQLPSPVEDSEQLRKAFQGWGTNEGLIISILGHRNAAQRKLIREAYSATHGEDLFKDLDKELSSDFERAVL

Gm08g14460 PAQLPSPLEDSEQLRKAFQGWGTNEGLIISILGHRNAAQRKLIREAYSTTHGEDLLKDLDKELSSDFERAVM

At1g35720 SDSVPAPSDDAEQLRTAFEGWGTNEDLIISILAHRSAEQRKVIRQAYHETYGEDLLKTLDKELSNDFERAIL

At5g10230 PATVPLPEEDAEQLYKAFKGWGTNERMIISILAHRNATQRSFIRAVYAANYNKDLLKELDRELSGDFERAVM

At5g10220 PANIPLPEEDSEQLHKAFKGWGTNEGMIISILAHRNATQRSFIRAVYAANYNKDLLKELDGELSGDFERVVM

Cp00036g01250 PESVPSVTEDCEQLNKAFSGWGTNEGPIISILAHINPNQCKLICQTYAETYGEDLLKALDKELTNDFERLVL

Cs217870 PDQLPPVAEDCDRLHSAFQGWGTDEGAIVSILAHRNAKQRSLIRQTYAETYGEDLLKALDKELSSDFERAVL

Cp00042g00660 ------------------------------------------------------------------------

PsABK22223 PTPTPTPVEDSESLRKAFEGWGTNEKLIIEILGHRTAAQRRAIRQAYTQLYEEDFLKRLQSELTREFERALF

Zm03g04200 PRVVPSPAEDAAALLKAFQGWGTDEQAVIGILAHRDATQREQIALEYEHKYGESLVQRLQSELTGDFERAVY

Bd2g13620 PQVIPSPTEDADALMKAFQGWGTDEQAVISILAYRDAEQRKQIRLAYQEKYDESLLQRLQSELTGDFQTAMC

Os01g31270 PPVTPSPAEDADALLKAFQGWGTDEQAVIGVLAHRDATQRKQIRLTYEENYNENLIQRLQSELSGDLERAMY

Zm08g03950 PRVVPSPAEDAAALLKAFQGWGTDEQAVISILAHRDATQRKQIALEYEHEYSESLIQRLQSELTGDLERAVY

Sb03g004990 PRVVPSPAEDAAALLKAFQGWGTDEQAVISILAHRDATQRKQIALEYEHKYSESLIQRLHSELSGDFERAVY

Gm13g26960 APNQKSPVEDVEALHKAFKGWGTDEKTVIAILGHRNVHQRQQIRKVYEEIYQEDLIKRLESELSGDFERAVY

Gm15g38010 APNQKSPVEDVEALHKAFKGWGTDEKTVIAILGHRNVHQRQQIRKIYEEIYQEDLIKRLESELSGDFERAVY

Cs308090 VPRDVPSNVDAEALRTAFKGWGSDEKAIISILAHRNAIQRRHIRIAYEQLFQEDLIKRLESEISGHFERAVY

Cs308080 VPHDVPPNVDAEAIKAAFRGWGTDEKAIVAVLGYRNAPQRRQIRIAYEQLFEEDLVKRFESELSGHLERAVY

Gm13g26990 APSNHSPQEDAEALRKAFEGWGTDENTVIVILGHRTVYQRQQIRRVYEEIYQEDLVKRLESEIKGDFEKAVY

Gm15g38040 APSNHSPQEDAEALRKAFEGWGTDEKTVIVILGHRTVYQRQQIRRVYEEIFQEDLVKRLESEIKGDFEKAVY

Mt8g038210 VQDNHSPNEDAEALRKAFEGWGTDEKTVITILGHRNSNQIQQIRKAYEGIYNEDLIKRLESEIKGDFEKAVY

Mt8g038220 APINHSPVADAEALHGAFKGWGTDEKSVITILGHRNVYQRQQIRKSYQEIYQEDILKRLESELSGDFERAVY

Gm11g21480 APSHHSRVEDAEALRNAFKGWGADDKAIIAILGHRNVHQRQEIRKAYEEIYQEDLIKRLESEISGDFERAMY

Mt3g018780 VHSQTSPVQDAEALRLAFKGWGADNKAIIAILGHRNVHQRQQIRKAYEELFEEDLIKRLESEISGDFERAVY

Gm04g27100 -------------------SWGADGKAIIAILGHRNATQRTLIREAYQNLFQEDLIKRLESELSGDFERAMY

Mt8g038180 APMNHSPKEDADVLWKAVKGWGTDESAIIAIMGQRNAVQRQQIRQAYQDIYQEDLIKRLESELSGNFEKAMY

Cp04842g00010 VPSQVSVVEDAEALNKAFKGWGTDEKTVIVILGHRNAAQRKQIRMAYEEIYQEDLIKRLESEISGDFE----

Cp36671g00010 VPSHVSVVEDAEALRKAVQGWGTDEKAIILLLGHRNSAQRKQIRIAYEEIYQEDLVKRLESELSGDFERAIY

Pt03g19020 VPENVSYADDAQALRKACQGWGTNEKAIISILGHRNAAQRKQIRLAYSELFQEDLVKRLESELNGDFEKAVY

Mt3g018920 --------------------------------------------------------------YAFDVVRAMY

Mt3g018790 APSNHSPVEDAEALQRAVKGWGADEKAIIAILGHRNGTQRTQIRQAYYELYQEDLIKRLESELSGDFERAMY

Cp00197g00010 VHAQASAVEDAEALRKAFAGLGTNEKAIISILGHRNAAQRKQIRVEYELLYKEDFLSRLESELTRDFKRAVY

Cp00157g00670 APKQFSPVEDAENIKKACLGWGTDERAIISILGHRNVFQRKLLRLAYQEIYQEDLIQQLKSELSGDFERAIC

Cs138380 TPKYFSPVEDAENIKKACLGLGTDENAIISILGHRNATQRKLIRLAYEEIYNEDLIQQLNSELCGDFERAIC

Pt01g27650 APKDFSPVEDAETIKKACLGLGTDEKAIISVLGNRNSFQRKLIRLAYEEIYHEDLIHQLKSEISGDFERAMS

At5g12380 SPPHFSPVEDAENIKAACQGWGTNENAIISILGHRNLFQRKLIRQAYQEIYHEDLIHQLKSELSGNFERAIC

Vv06g10680 APEDFSPGEDALAINRACQGWGTDEKAIISILGHRNAAQRKQIRLAYQEIYLEDLTKQLKSELSGDLERAIC

Gm11g21460 APSNHPPVEDTESLRKAVKAFSHKNRVQLVLKDSRSTYSRISAKRISSNALSQSLVTLREFFFFFSIDKSMY

Gm13g27000 APRNHFPQEDAEALWKAVKGWGTDEKTIIKILGHRNASQRQQIRLVFQDIHLEDLVKRLESELSGDFERAVY

Gm15g14350 AAKHSSSIEDAENIKKACKGLGTDETALISILAHRNVAQRKLVRMAYEELYQEDLIQQFKSELSGSFERAIC

Os05g31750 ---MADEIQHLTRAFSGLGGLGVDEPAMVSALAKRQPEKLSGFRKSFNGFFKDEYMLHLAAE-FSRFKNLMV

Bd2g26760 ---MADEVQALTKAFSGLGGLGVDETTMVSTLAQKQPEKRSGFRKSFRGLFKEEYMLHLAAE-FSRFKNLMV

Zm06g23270 ---MADEVQQLTRAFSGLGGLGVDEPAMVSALARGQPEKRSAFRKGFPGFFSSEYMLHLAAE-FARFRDLVV

Bd1g62130 ---MADEQQELTRAFSGLGGLGVEETALVSALGRKQPEKRASFRRGFPGFFSPEYVRHLKTE-FSRFKNLMV

Zm01g15800 KEAMADEHQDLTRAFAGLGGLGVDETALVSALGRREPEKRAQFRRGFPGFFSSEYLLHLKAE-FARFKDAAV

Sb01g035050 QSKMADEHQDLTRAFAGLGGLGVDETALVSVLGRRQPEKRAQFRRGFLGFFSAEYLLHLKAE-FARFKDAAV

Vv00g25070 -------SHEFQALTKSFSGFGVDEKSMISILGKWHQDDRKSYRKGCPQFFTQRHVAFLKHE-FLRLKNAVV

Cs308100 MADSAVEVLTRALS-----GHGINENAMIETLGKWDHEEKKLFRKKSSHFFSEHGMRLLKHE-FMRFKNAVV

Gm13g27010 -------NQELEAVTQAFSGHGVDEKSLVTLLGKWDPLERESFRKKTPHLFSEQYVRLLKHE-FVRFKNAVV

Gm15g38060 -------NQELEAVTQAFSGHGVDEKSLVTLLGKWDPLERESFRKKTPHLFSEQYVRLLKHE-FVRFKNAVV

Mt8g038170 -------NQELEAITQAFSGHGVDEKSLIAVLGKWDPLERETYRKKTSHFFIEHCVRLLKHE-FVRFKNAVV

Pt01g06030_ -------MANLEALAKAFTGLGVDEKSLIENLGKSHPEQRTLFRKKTPQLFIEHCVRLLKHE-FVRFKNALV

At2g38750 ---LPLELESLTEAISAGMGMGVDENALISTLGKSQKEHRKLFRKASKSFFVEDEERAFEKCHDHFVRTAVV

Sb09g018980 PNPVPSATQDAENIRKAVQGWGTDEKALIEILGHRTAAQRAEIAVAYEGLCNESLLDRLHSELSGDFRSAMM

Bd2g26770 PDPVPAPTEDAENIRKAVEGWGTDEKALIEILGHRTAAQRAEIAVAYEGLYDQPLIGRLQDELSSHFRGAMM

Zm06g23280 PSRAPSAAEDAENIRKAVQGWGTDEKALIEILGHRTAAQRAEIAVAYEGLYNEPIIDRLHSELSGDFRSAMM

Zm08g13570 PDPVPSATEDAENIRKAAVGWGPDKKALMEILGHRTAAQRAEIAAAYAGRYNESLLDRLHSVLSGDFRSAMM

Os05g31760 PNPAPSPTEDAESIRKAVQGWGTDENALIEILGHRTAAQRAEIAVAYEGLYDETLLDRLHSELSGDFRSALM

Bd1g62120 PTPLPSPAADA-----------------------------------------------------------ES

Sb01g035040 ----------------------------------------------------------------------MV

Sb02g041850 PSPPPTASEDAESLRTALQGWRADKAALIGVLCRRTAAQRAAIRRAYAFLYREPLLNCFRYKLSRHCIKAMI

Bd1g18990 PSPVPSPAEDAEGIWKALQGKY--------------------------------ACNCIVFSFQLY-HKAMI

Os07g46550 PSPVPSASDDAESLRKALQGWRADKGALTRILCRRTAAQRAAIRRAYAFLYREPLLNCFRYKLSRHCLKAMI

Pt01g06020 PEVVPSPTQDCEKLRDAVQGLGTDEKAIIWILGHRNASQRKKIRETYQQLYNESLIDRLNSELSGDFRKAVI

Gm15g38070 PEVVPSPTQDSERLRKAFQGFGTDEKAVILVLGHRNAQQRKKIGETYQQLYNESLVDRLHSELSGDFRNAVI

Gm13g27020 PEVVPSPTQDSERLRKAFQGYGTDEKAVILVLGHRNAQQRKEIRETYQQLYNESLIDRLNSELSGDFRNAVI

Mt8g038150 PEIVPSPNTDTERLRNAFQGIGTNEKELILVLGHRNAQQRREIRETYQKLYNESLLDRLQSELSGDFRNAIV

Vv00g25060 PDAVPPPAQDCEKLQKAFQGWGTDEKAIIWVLGHRNASQRRIIRDTYQHLYNESLIDRLQSELSGDFRNAVV

Cp00197g00020 SDTVPSPTEDSHTLKEAFQGFGTDEKAIIKVLGARTARQRREIRDTYQQLYNENLIDALFSELSGDFRKAVI

At2g38760 PNEVPSPAQDSETLKQAIRGWGTDEKAIIRVLGQRDQSQRRKIRESFREIYGKDLIDVLSSELSGDFMKAVV

Vv00g00800 PDSIPSPAQDSERLSLALQGRGVDEKVIVWILGHRNAIQRKRIKDTYQQLYKESIIHRLQSKLSGVLKKAMS

Vv03g02080 PDSIPSPAQDSERLNLALQGRGVDEQVIVWILGHRNAIQRKQIKDTYQQLYKESIIHRLQSKLSSGLKTAMI

Vv00g00650 PDSIPSPVQDSERLNQALQGRGVDEKVIVWILGHRNAIQRKQIKDTYQQLYKESIIHRLQSKLFGVFKTAMI

Vv00g00720 ------------------------------------------------------------------------

Vv00g00760 ---IPGPSNGS--------EAGLLLFI---------------------------------------------

Vv00g00750 PDVVPSPTQDSERLRVALQGWGVDQEVIIWILGHRKAVQRKKIKETYQQLFKESIIHCLQSTLSGVLGKAMS

Vv00g00710 PAVAPSPTQDSERLRVALQGWGVDQEVIIWILGHRNAVQRKKIKETYQQLFKESIIHCLQSTLSGVLGKAMS

Vv00g00660 PDVAPSSTQDSERLRVALQGWGVDQEVIIWILGHRNAVQRKKIKETYQQLFKESIIHCLQSALSGVLGKAMT

Cs307980 -----------MILPEAGLGIGIDEKKLVDMVRR-SDFNPGNIKRRRELIMI---------E-FQRFMNATL

Cs307970 CAKNHSPFESKDAFEQSLAGVGINENGIVKTLTNFDADEYRLMSSNFDQSLGYIWKEKKRQSMKLEFQNVTM

**Figure S2B**

Bd4g31920 LWMLDPAERDAIMARDAIESG-TGYRALVEIFTRRKQEQLFFTKQAYLGRFKKNMEQDMVTEPSPSRPYLLV

Os09g27990 LWVLDPSERDAIMARDAVENGGTDYRVLVEIFTRRKQNQLFFTNQAYLARFKKNLEQDMVTEPSHPYQRLLV

Zm02g31380 LWALEPAERDAVVAREAVEGGVAGYRALVEVFTRRKQDQLFFTKQAYAVRFRRSLDQDMATEPSHPYHRLLL

Sb02g026390 LWGALPAERDAVVAREAVEGGVAGYRALVEVFTRRKQDQLFFTKQAYMARFRRNLDQDMVTEPSHPYQRLLL

Cs340270 LWMLDSHERDAVFAREALEPGDTNFKALIEIFVGRKSSQIFLIRQSYQARYKKQLDQDIINIDPHSYQKILV

Pt13g04990 RWMMDTYERDAIVAREAFGQGDVNYKALVEIFVGRKSSHMVLIKQAYYARFRRHLDQEIINLEPHPYQKILV

Cp00161g00040 MWMLDSCERDAVVAREALKQDDIDFKALVEIFVGRKSSHIALMKQAYQARFKSQLEQDIINIDPHSFQKILV

Vv08g00710 GL--DPHPSDAVVVREALEQGDTNYKVLVEIFVWRKSSQILLMKQDYGARFRRQMDQDIINIEPHPYQKILV

Gm13g26040 LWMLDTHDRDAVVAREALQQDETNFKALVEIFVGLPENVQKTLGPRYYQFGSKGERYIIRNHSIGLWELIIV

Os09g20330 CLRASPCVRDADIARDALFGRRIDGDVLVEVVCTRPSGEVALIRQAYQARYSASLERDVSSRTSGSLNEVLL

Cp00042g00810 LRMSKPQERDAEIMRHSLFGGRINLHNLIEVACTRSSLELHFIKQAYNSRFNSNLEQDMGTKLNSGFKEILL

Os09g23160 LWILDPAGRDATVLREALSGDTIDLRAATEIICSRTPSQLQIMKQTYHAKFGTYLEHDIGQRTSGDHQKLLL

Bd4g29680 LWILDPAGRDATVLREALSADSLDLRAATDIICSRTPSQLQIMKQTYYAKFGTYVEHDISQQTTGDHQKILL

Sb02g024090 LWILDPAGRDATVLREALSGDTMDLRAATEIICSRTPSQLQIMKQTYYARFGTYLEHDIGHHTSGDHQKLLL

Zm02g30240 LWILDPAGRDATVLREALNGDTMDLRAATEIICSRTPSQLQIMKQTYYARFGTYLEHDIAHHTSGDHQKLLL

Zm07g13390 LWILDPAGRDATVLREALSVDTMDLRAATDIICSRTPSQLQIMKQTYYARFGTYLEHDIGHHTSGDHQKLLL

Bd3g36240 LWILDPAGRDATILNQALNSDIPDLRAATEIVCSRTPSQLQIMKQTYRVRFGCYLEHDITERAYGDHQRLLL

Zm04g13650 LWILDPATRDATILKQALTGDITNLRAATEIVCSRTPSQLQIMRQTYRARFGCYVEHDVTERTSGDHQRLLL

Sb07g020760 LWVLDPATRDATVLKQALTGDVTDLRAATEVVCSRTPSQLAVVRHAYRARFGCHLEHDVTERTSGDHQRLLL

Os08g32970 LWVLDPASRDAAVLHQALNGDVTDMRAATEVVCSRTPSQLLVVRQAYLARFGGGLEHDVAVRASGDHQRLLL

Cs234810 LWMYDPGTRDAVIVKEALSGDTIHLRRATEVLCSRTSTQIQHVRQIYLSMFQSYIEHDIEKSASGDHKKLLL

Cs234800 LWMYDPATRDAILVKNAIYGETSTLRAATEVICSRTPSQIQHFKQIYLAMFRSPLERDIERTATGDHLKLLL

Vv01g05380 LWVQDPAGRDASIVRQALSGNVVDLKAATEVICSRTPSQIQHFKQLYFAMFGVYLEQDIEYQASGDHKKLLL

Gm08g06100 LWMHDPAGRDAIILRQSLTLP-KNLEAATQLICSRTPSQLHYLRQIYHSKFGVYLEHDIETNTSGDHKKILL

Mt8g107640 LWMHDPATRDAIILKQTLTVS-KNLEATTEVICSRTPSQLQYLRQIYHTRFGVYLDHDIERNASGDHKKILL

Gm07g12030 LWLHDPAGRDATIIRKSLTADNRSIEGATEVICSHTPSQLQYLKQIYHSMFGVYLEHDIQTNTSGDHQKLLL

Gm09g30190 LWLHDPAGRDATIIRKSLTADNKTLEGATEVICSRTPSQLQYLKQIYHSMFGVYLEHDIQTNTSGDHQKLLL

Pt15g04350 FWMHDLPGRDAIIVRQALMMNTMNLEAATEVICSRTPSQIQVFKQHYHAKFGIHLERDIESCASGDHKKLLL

Pt12g03690 LWMHDLPGRDAIIVRQALIADILNLETATEVICSRTSSQIQVFKQHYYAKFGVHLEHDIELRASGDHKKLLL

Cp00003g03400 LWILDPAARDATAIRQAYVA--ADLRALTEIICSRTPSQIQLIKQHYHSQVGIHLEEEIQQQTPDDHQKLLL

Gm07g28080 LWLHDPATRDAKVVRKALTISVVDNQAITEIICSRTPSQLRRLKEVYLSTYHSYLEQDIESKTSGDHKKLLL

Gm20g01460 LWLHDPATRDAKVVRKALTASVVDNQALTEIICSRTPSQLRRLKEVYLSTYHSYLEQDIENKTSGDYKKLLL

Cp00002g01210 LWMNDPVTRDAKILAQALRGSLTDHKAVTEVICTRTTAQLRQIKQVYSKDYGTTPEHDIESKCYGDHKRLLL

At1g68090 LWMPEAVERDASILKRSLRGAVTDHKAIAEIICTRSGSQLRQIKQVYSNTFGVKLEEDIESEASGNHKRVLL

Pt10g10090 LWMKSPVERDVTTLRQALTGPIIDIKTATEIICTRISSQIRQIKQVYTPTFGTLLEYDIGYHTSGDHRKFLL

Pt08g13700 LWMKSPIERDVTTLRQALTGPLFDVKAATEIICTRTSSQIRQIKQVYTPTFGTRLEYDIGCHTSDDHKKLLL

Sm271856 LWMMEPAERDAVLIRDAMKGLGTKDKTLIEIICSRTPSQLYYIRQAYQTKYHRSLDKDIQSDTSGDYRKLLL

Sm167346 LWMMEPAERDAVLIRDAMKGLGTKDKTLIEIICSRTPSQLYYIRQAYQTKYHRSLDKDIQSDTSGDYRKLLL

Sm124402 LWMMEPAERDAVLLRDAMKGLGTKDKTLIEIICSRTPSQLYYIRQAYQTKYHRSLDKDIHSDTSGDYRKLLL

Sm94768 LWMLEPAERDAVLIRDAMKGLGTKDKTLIEIICSRTPSQLYYIRQAYQTKYHRSLDKDIQSDTSGDYRKLLL

Sm227533 LWMMEPAERDAVLLRDAMKGLGTKDKTLIEIICSRTPSQLYYIRQAYQTKYHRSLDKDIQSDTSGDYRKLLL

Pp1s219_3V6 LWMMGPAQRDAILIYDSMKGLGTKDSALIGIICTRTPSQIYEIKQAYQAMYQQALESQVSGDTSGDYRKLLL

Pp1s37_276V6 LWMMTPAQRDATLVNESMNGLGTTDHALVGIICTRTPSQHYAISQAYNAMFRHTLERKIDGDTSGNYRKLLL

Pp1s6_292V6 LWMMDSAERDAILLYELMKVGGKADRAFIGIVCTRNSAQIYLIKQAYYTMFNQTLENHIDGTDSPKRLVLML

Pp1s102_141V6 LWMMDSAERDAILMHELMKVGGKADRSLIGLVCTRNSAQLYLIKQAYYTMFNQTIENHMDGTDSPKRLVLLL

Pp1s1_594V6 LWMMDSAERDAILMYELMKIGGKADRALIGIVCTRNPTQIYAIKQAYYTMFNQTLENHIDGTNSHKRPVLLL

Pp1s61_299V6 LWMMDPSERDAVLLYEALSQGGKKDRAVIGMLCTRSSAQLYLIKQAYYSVFCQTLENHLDGSGFPKRQLLLL

Pp1s38_63V6 LWMMDPFDRDAVLLNEALREGGKKDRVIIGMLCTRTSKQIYLIKQAYYTMFNQTLESHIDGSGFPKRVLLLL

Ot11g03220 ALAKRDYDFEIQSMRNAVEGLGTDEQLIISILANKTHEQIEEFREAYKVATGELLRERIRDETTGLFESKLF

MRCC299_56760 LAGCDIAEAYAKVCRSAIEGAGTDCKALIRLMVTCTHQVMYDTRKAYGRLYNLDLARDMSGEWAGDFKNILC

PsABK21977 LWMHDPAERDAIIAKTELRSQYPDFRALTEILCSRTPAETLRIREAYRGLYKACLEEDIAQETVGPHQKLLF

PsACN40166 LWMHDPAERDAIIAKTELRSQYPDFRALTEILCSRTPAETLRIREAYRGLYKACLEEDIAQETVGPHQKLLF

Cp00213g00130 LWTLDPPERDAYLANESTKRFTSSNWVLLEIACTRSSLELFKVKQAYHARYKRSLEEDVAYHTKGDFRKLLV

Bd3g58830 QWTLDPAERDAVLASEEARKWHPGGRALVEIACARTPAQLFAARQAYHERFKRSLEEDVAAHATGDFRKLLV

Os02g51750 QWTLDPAERDAVLANEEARKWHPGGRALVEIACTRTPSQLFAAKQAYHERFKRSLEEDVAAHITGDYRKLLV

Sb04g027590 LWTLDPAERDAVLANEEAKKWHPGGRALVEIACARTPAQLFAAKQAYHDRFKRSLEEDVAAHVTGDFRKLLV

Zm05g40790 LWTLDPAERDAVLANEEAKKSHPGGRALVEIACARTPAQLFAVKQAYHDRFKRSLEEDVAAHVTGDFRKLLV

Bd1g45487 LWTLDPAERDAVLANEGAKKWHPGSPVLVEIACARGSGQLFAVRQAYHERFKRSLEEDVAAHVTGAFRKLLV

Sb10g007760 LWTLDPAERDAVLANEAARKWQPGNRVLVEIACTRTSAQVFAARQAYHERFKRSLEEDIAAHVTGDFRKLLV

Os06g11800 LWTLDPAERDAVLANEVARKWYSGSRVLVEIACARGPAQLFAVRQAYHERFKRSLEEDVAAHATGDFRKLLV

Zm06g16450 LWTLDPAERDAVLANEAARKWKPGNRVLVEIACTRTSAQIFATRQAYHERFKRSLEEDIAAHVTGDFRKLLV

Cs273000 LWTLEPADRDAFMVNEATKRLTSNNLVIVEVACTRTSIELFKVRQAYQARFKRSVEEDVAYHTSGDIRKLLV

At5g65020 LWTLDPPERDAYLAKESTKMFTKNNWVLVEIACTRPALELIKVKQAYQARYKKSIEEDVAQHTSGDLRKLLL

Gm13g01870 LWTLDSAERDAFLANEATKKWTSSNQVLVEIACTRSSEQLFAARKAYHVLYKKSLEEDVAHHTTGDFRKLIL

Mt5g063670 LWTLESAERDAFLANEATKRWTSSNQVLVELACTRSSDQLFFAKKAYHALHKKSLEEDVAYHTTGDFRKLLL

Vv18g03470 LWTLDPAERDAFLANE----------------------------QAYHARFKRSLEEDVAYHTSGDFRKLLV

Pt02g09420 LWTLDPAERDAALANEATKRWTSSNQVLMEIACTRSSNELLLARQAYHARFKKSLEEDVAHHTSGDFRKLLF

Pt07g05300 LWTLDPAERDAYLANEATKRFTSSNWVLMEIACTRSSHDLFKVRQAYHARYKKSLEEDVAYHTTGDFRKLLV

Pt05g07550 LWTLDLAERDAYLANEATKRFTSSNWVLMEIACTRSSHDLFKARQAYHARYKKSLEEDVAYHTTGDFRKLLV

Gm05g31250 VWTLDPAERDAFLANEATKMLTSNNWVILEIASTRSSLDLLKAKQAYQARFKKSLEEDVAYHTKGDIRKLLV

Gm08g14460 VWTLDPSERDAFLANEATKMLTSNNWVILEIASTRSSLDLLKAKQAYQARFKKSLEEDVAYHTKGDIRKLLV

At1g35720 LWTLEPGERDALLANEATKRWTSSNQVLMEVACTRTSTQLLHARQAYHARYKKSLEEDVAHHTTGDFRKLLV

At5g10230 LWTFEPAERDAYLAKESTKMFTKNNWVLVEIACTRSALELFNAKQAYQARYKTSLEEDVAYHTSGDIRKLLV

At5g10220 LWTLDPTERDAYLANESTKLFTKNIWVLVEIACTRPSLEFFKTKQAYHVRYKTSLEEDVAYHTSGNIRKLLV

Cp00036g01250 LWTLRPAERDAFLANEARK---------------RSSNQLLQAGQAYHARFKRSLEEDVAYHTKENFRKLLV

Cs217870 LWTFHPAERDALLANEAIRK--LKHFVVLEIACTRTPRDLLLVKEEYHARFKRSIEEDVAHYTTGDFRRLLV

Cp00042g00660 ---MDPAECDALLANESLRKRAAGHHVIMEIACTRTSHEVFL------------------------------

PsABK22223 LWSLDPPERDALLAHESIKKWSPKNRSLIEISCARSSSELWLVRQAYHVRYKKSLEEDIASHTQGDFRKLLV

Zm03g04200 HWMLGPAERQAVMANAATECLQEECAVIVEIACANSSAELVAVKKAYHALYRRSLEEDVAARATGNLRSLLL

Bd2g13620 HWVLDPVERQAAMANAATKCIHEEYPVIVEIACANSPTELLKVKQAYHALYKCSLEEDVAASAPGNLRSLLL

Os01g31270 HWVLDPVERQAVMVNTATKCIHEDYAVIVEIACTNSSSELLA---------------------------LLL

Zm08g03950 HWMLGPAERQAAMAHAATECVQERYAVVVEIACANSSAELVSVKQAYHVLYRRSLEEDVAARATGNLRSLLL

Sb03g004990 HWMLDPAERQAVMANAATECIQEEYPVLVEIACANSAAELVAVKKAYHALYKRSLEEDVAARATGNLRTLLL

Gm13g26960 RWMLEPADRDAVLANVAIKNGSKGYHVIVEIACVLSADEVLAVKRAYHNRYKRSLEEDVATNTTGDIRQLLV

Gm15g38010 RWMLEPADRDAVLANVAIKNGSKGYHVIVEIACVLSAEEVLAVKRAYHNRYKRSLEEDVATNTTGDIRQLLV

Cs308090 RWMLDPEDRDAVLANIAIRKPKEDFAVLVELSCIYSPEELLGVRRAYQHRYKRSLEEDVAASTNDDLRTLLV

Cs308080 RWILDPEDRDAVLAHVALRKPNEDFAVLVEFSCIYSPEEFLGVRRAYQHRYKRSLEEDVAANTHDDFRKLLV

Gm13g26990 RWILEPADRDAVLANVAIKSG-KNYNVIVEIATILSPEELLAVRRAYLNRYKHSLEEDVAAHTSGHLRQLLV

Gm15g38040 RWILEPADRDAVLANVAIKNG-KNYNVIVEIATILSPEELLAVRRAYLNRYKHSLEEDVAAHTSGHLRQLLV

Mt8g038210 RWILEPAERDAVLANVAIKSG-KNYNVIVEISAVLSPEELLNVRRAYVKRYKHSLEEDLAAHTSGHLRQLLV

Mt8g038220 RWMLEPADRDAVLANVAIKDGSKSYHVIIEIVSVLSPEEVLAMRRAYHNRYKHSLEEDLAAHTTGHLRQLLV

Gm11g21480 RWMLQPADRDAVLVNVAIKNGTKDYHVIAEIACVLSAEELLAVRRAYHRRYKCSLEEDVAANTTGNLRQLLV

Mt3g018780 RWMLDPADRDAVLINVAIRNGNKDYHVVAEIASVLSTEELLAVRRAYHNRYKRSIEEDVSAHTTGHLRQLLV

Gm04g27100 RWILEPAEREALLANIAIKSADKNYQVIVEISCVLSPEELFAVRRAYHNKYKRCLEEDVAANTSGHLRQLLV

Mt8g038180 RWILDPADRYAVLANVAIKSINKDYHVIVEIASVLQPQELLAVRHAYHNRYKNSLEEDVAAHTSGYHRQLLV

Cp04842g00010 ------------------------------------------------------------------------

Cp36671g00010 RWVLEPADRDAVLCHVAIRKHEPDYHVLVEISCIRSPEELIAIRRAYQIRYKCSLEEDVAAHTSGDIRKLLV

Pt03g19020 RWVLDPEDRDAVLANVAIRKS-GDYHVIVEIACVLSSEELLAVRRAYHARYKHSLEEDLAAHT---------

Mt3g018920 RWILEPAEREA--------------------------------SRAYHNRYKRSLEEDVATNNNGYLRQLLV

Mt3g018790 RWILEPAEREALLANIALRNANINYHLIVEISCVSSPDELFNLRRAYHNRYKRSLEEDVATNTNGHLRQLLV

Cp00197g00010 LWMLEPADRDAVFVHEAIKKHKIEYQVLIEISCTRSPEELFAIRRAYQ---------------------LLI

Cp00157g00670 HWTLDPADRDAVLANLALQKPVPDYKVIVEITCIASPDDLLAVKRAYRSRYKRSLEEDVASYTTADIRTLLV

Cs138380 HWTLDPADRDATLANKALKSSTLDYRVIIEIACVQSAEDLLAVKRAYRFRFKRSLEEDVASCTTGNMRKLLV

Pt01g27650 QWTLEPADRDAVLANAALQKSKPDYRVIVEIACVGSPEDLLAVKRAYRFRYRHSLEEDVALHTKGDIRKVLV

At5g12380 LWVLDPPERDALLANLALQKPIPDYKVLVEIACMRSPEDMLAARRAYRCLYKHSLEEDLASRTIGDIRRLLV

Vv06g10680 HWILDPVERDAVLANEALKKARPDYRVILETAYMKSPEELLAVKRAYQFLYKRSLEEDVASHTTGDMRRLLI

Gm11g21460 RWILEHVEREALLANIALKSADKNYQVIVEISCVLSPEELFVVRRAYHNKYKRSLEEDVAANTSGHLRQILV

Gm13g27000 RWTLEPSKRYAVLANVAIKNANKDYHVMVEIVCVR---------RAYHNRYKHSLE-DVAAHTTDHVRQASM

Gm15g14350 NWTMDPAERDAAFINEALKKETPDYKVIVEIVCTRTSEEFLAAKRSYQFQYKHCLEEDVASKTIGDIRRLLV

Os05g31750 MWAMHPWERDARLAHHVLHQA-HPAAIVVEIACTRTAEELLGARKAYQALFHHSLEEDVAYRARKPYCGLLV

Bd2g26760 LWAMHPWERDARLAHHVLHQA-HPPAIAVEIACTRSAEDLLGARKAYQALFHHSLEEDVAFHAKKPYCSLLV

Zm06g23270 LWATHPWERDARLAHHVLHHHHHPPAVVVEVACARSADELLGARRAYQALFHRSLEEDVAHRARKPYCSWMD

Bd1g62130 LWAMHPWERDARWAHRALHKHKGSGCILVELACTRSAEELLGARRAYHALYSRSLEEDVAYRLKTEHAGLLV

Zm01g15800 LWAMHPWERDARWAHHVLHKA-HPPHILVEVACTRTADDLLGARRAYQALYHRSLEEDVAYRVRDANASLLL

Sb01g035050 LWAMHPWERDARWAHHVLHKA-HPPQVLVEVACTRAADDLLGARRAYQALYHRSLEEDVAYRVRDANASLLV

Vv00g25070 LWTMHPWERDARLMKEALVKGPQAYAVIIEVASTRSSEQLLGARRAYHSLFDHSIEEDVAYHINDSCRKLLV

Cs308100 LWTTHPWERDARLVKEALSKGHQNINILIEVACTRTSDELLGARKAYHSLFDHSIEEDVASHLNGPERKLLV

Gm13g27010 LWTMHPWERDARLVKEALKKGPNEYGVLIEVACTRSSEELLGARKAYHSLFDHSIEEDVASHIHGIERKLLV

Gm15g38060 LWSMHPWERDARLVKEALKKGPNAYGVLIEVSCTRSSEELLGARKAYHSLFDHSIEEDVASHIHGIERKLLV

Mt8g038170 LWSMHPWERDARLAKEALKKGSISYGVLIEIACTRSSEELLGARKAYHSLFDHSIEEDVASHIHGNDRKLLV

Pt01g06030 LWAMHPWERDARLVKEALKKGPQSYGVIVEIACTRSSEELLGARKAYHSLFDQSIEEDVATHIHGSERKLLV

At2g38750 MWAMHPWERDARLVKKALKKGEEAYNLIVEVSCTRSAEDLLGARKAYHSLFDQSMEEDIASHVHGPQRKLLV

Sb09g018980 LWTADPAARDAKLAHKAMKKKGRYVWVLIEVACASTPDHLVAVRKAYREAYSASLEEDVAACPLNKDPLFLV

Bd2g26770 LWTMDPAARDAKLAYKALRKKGRHAWVLIEVACASSPDHLVAVRKAYCSAYESSLEEDVAACSLKDPLKFLV

Zm06g23280 LWTVDPAARDAKLAHKAMKKQGRYVWVLIEVACASAPDHLVAVRKAYREAYSASLEEDVAACPLKDPLLFLV

Zm08g13570 LWTADPAARDAKLAHKAMKKKGRYVWVLIEVACASTPDHLVAVRKAYRESYPASLEEDVAACPLKDPRVFLV

Os05g31760 LWTMDPAARDAKLANEALKKKKRHIWVLVEVACASSPDHLVAVRKAYRAAYASSLEEDVASCSLGDPLRFLV

Bd1g62120 LWNA-------------------------------------------------------------VQGNLLV

Sb01g035040 LWTIDPAERDARLANQALGDRRQHAWVLVEVACASAPDHLIAVRRAYRSLFGCSLEEDVAACPAQDPLRLLV

Sb02g041850 LWTMDPAERDANLVHEAVKKKK-YVSVLVEVSCASTPDHLMAVRNIYRKLFSSSVEEDVASSPAQEPLKMLL

Bd1g18990 LWTMDPAERDANLLHGAIRLRGDHVFVLVEISCASAPDHLVAVRRAYASLFGCSLEEDLASSVSQEPLKLLV

Os07g46550 LWTMDPAERDANLVHEALKKKQYYMSVLIE---------------------------------------MLV

Pt01g06020 LWTTDPPERDAKLANEALKANKKQLQVVVEITCASSPNHLQEVRQAYCSIFDCSLEEDIVSAVPLPLRKILV

Gm15g38070 LWTYDPPERHARLAKDALKAKKKHLQVLVEIACASTPNHLVAVRQAYCSLFDCSLEEDIIASVAPALRKLLV

Gm13g27020 LWSYDPPERHAGLAKDALKAKKKHLQVLVEIACASTPNHLVAVRQAYCSLFDCSLEEDIIASVAPPLRKLLV

Mt8g038150 LWTCDPPERDAKFARDALKVKRKQLQILVEIACASSPNHLMAVRQAYCSLFDCSLEEDIIASVSQPLTKILV

Vv00g25060 LWTYDPPERDARLAKEALKARKNHLQVIVEIACASSPHHLMSVRQAYCSLFESSLEEDITANVSLPLKKLLV

Cp00197g00020 LWTIDPPERDAKLANEALQKKKKNLKVIVEIACASSPHHLMAVRQAYSSLFDCSLEEDIVSAVPPLIAKVLV

At2g38760 SWTYDPAERDARLVNKILNKEKENLKVIVEISCTTSPNHLIAVRKAYCSLFDSSLEEHIASSLPFPLAKLLV

Vv00g00800 YWMEEPPERDAKLVEKTLKRGKTQLQVIVEIACASSPNHLMAVRQAYCSLFDCSLEEAITSKVSSSLQKLLL

Vv03g02080 LWMNEAPERDAILANKALKRKRNQLQVLVEIACASSPDHLMAVRQAYCSLYECSLEEDITSNISTSLQKLLV

Vv00g00650 LWMNEAPERDAILANMALKRKRNQLQVLVEIACASSPDHLMAVRQAYFSLYECSLEEDITSNISTSLQKLLV

Vv00g00720 -----------------------------------------------------MLQTN---------IKLLV

Vv00g00760 --------------------------------------RMLSGRRHHIQHLNFSPKGD-----------ISL

Vv00g00750 YWMEEPPERDAKLVEKTLKRGKTQLQVIVEIACASSPNHLMAVRQAYYSLFDCSLEEAITSKVSSSLQK---

Vv00g00710 YWMEEPPERDAKLVEKTLKRGKTQLQVIVEIACASCPNHLMAVRQAYCSLFDCSLEEAITSKVSSSLQKLLL

Vv00g00660 YWMEEPPERDAKLVEKTLKKGKTQLQVIVEIACASSPNHLMAVRQAYCSLFDCSLEEAITSKVSSSLQKLLL

Cs307980 MWMTSPAERDARLLRKAIKTRGVGIMVIIEITCTREFCDVSAAKDVYHHLYKSLLEFDLSRYIVGPEQTLLN

Cs307970 LWMTTPIERDARLLRSALKMGDAGVSVLIEIVCTRPFADFLAIKYLYGKLFKSDLLFDLDQHVPGKAVRCLI

**Figure S2C**

Bd4g31920 DEPSWHIAKCDARRLYDAKKGGTSVDEATILEMFSKRSIPQVRLAFSSYKHIYGHDYTKALKKNVFGEFEESL

Os09g27990 DELSRHIAKCDARRLYDAKNSGMSVDEAVILEMFSKRSIPQLRLAFCSYKHIYGHDYTKALKKNGFGEFEQSL

Zm02g31380 DDLSQHVAKCDARRLHDTKN-SGVVDEAVILEMFSKRSIPQLRLAFCSYKHIYGHDYTKALKINGSGEFEGPL

Sb02g026390 DDLSQHVAKCDARRLHDTKNS--VVDEAVILEMFSKRSIPQLRLAFCSYKHIYGHDYTKALKINGSGKFEESL

Cs340270 ADISQHIAKCDARKLYETVKDNSGAIEEFVLEMLTKRSIPQLKLTFSCYQHIFGHNFTKDLKFRNCGEFENAL

Pt13g04990 EDVSQHIAKCDARRLYEAGEG--AVEEAVVLEILSKRSIPQTKLTLSSYKHIYGHEYTKSLKNAKYMEFEDAL

Cp00161g00040 MDVSQHVAKCDARRLYEVGEG--GIEEAVVLEIFSKRSIPQLKLTFSCYKHIYGYDYIKSFKRENSTEFENTL

Vv08g00710 ADVSQHIAKCDARRLYEAGVGKSGTEEAVVLEILSKRSIPQLKLTFSCYKHIYGHDYTKLLKKENSGEFEDAF

Gm13g26040 ADVNHHISKCDARRLYETGEGSLTVIEAVVLEILSKRSIPQLKLTFFSYKHIYGHDYTKSIKRGKYGQFGKAL

Os09g20330 GRVDATMAMCDAKTLYEAVEISARVDQRSVLQLLRHRSGDQLRAVLASYRRLYGQELARALKRKDGDTFPGIL

Cp00042g00810 GKADTSMAMCDAKTLYEAVETGKTIDQRSIVLIMSQRNTGQIKAILSSYRQLYGHEFSKAIKQSKCGQFGKEL

Os09g23160 PEVDPTIVTHDAKDLYKAGEKRLGTDEKTFIRIFTERSWAHMASVASAYHHMYDRSLEKVVKSETSGNFELAL

Bd4g29680 PEVDPTIVTHDAKDLYKAGEKKLGTDEKTFIRIFTERSWAHMAAVASAYHHMYDRSLEKVVKSETSGNFEVAL

Sb02g024090 PEVDPTIVTHDAKDLYKAGEKRLGTDEKTFIRVFTERSWAHLASVSSAYHHMYDRKLEKVVKSETSGNFEFAL

Zm02g30240 PEVDPTIVTHDAKDLYKAGEKRLGTDEKIFIRVFTERSWAHLASVSSAYHHMYDRKLEKVIKSETSGNFEFAL

Zm07g13390 PEVDPTIVTHDAKDLYKAGEKRLGTDEKTFIRVFTERSWAHLASVSSAYHHMYDRKLEKVIKSETSGNFEFAL

Bd3g36240 --------------LYKAGEKRLGTDERTFIRIFSERSWAHLASVASAYQHMYARSLEKAVKSETSGNFGFGL

Zm04g13650 HEVDPSTVTLDARDLYKAGERRLGTDERAFIRIFSQRSWAHMAAVARAYHHMYDRPLERAVKSETSGNFGFGL

Sb07g020760 AVVDASTVALDARDLYKAGERRLGTDERAFIRVFSERSWPHMAAVARAYHHMYDRSLESAVKSETSGNFGFGL

Os08g32970 PEVDMAAAARDARELYRAGERRLGTDERTFIRVFSERSAAHMAAVAAAYHHMYDRSLEKAVKSETSGNFGFGL

Cs234810 PEIDRNIVEKDAKTLYKAGEKRWGTDEQKFIQIFSESSRAHLAAVAYTYKQSYSNSLEKAIKSETSGYFEYGL

Cs234800 PEVDRALVDKDAKSLYKAGEKRLGTDEDKFIKIFSERSRAHLSAVSHAYKHSYGNSLKEVIKKETSGNFEHGL

Vv01g05380 PEVDRAMVEKDAKALYKAGEKKLGTDENTFIRIFSEKSRAHLAAVSTAYHSVYGNSLQKAVKSETSGHFEFAL

Gm08g06100 PEVNREMAEKDAKVLYKAGEKRLGTDEKTFVQIFSERSAAHLAAITSYYHSMYGHSLKKAVKKETSGNFALAL

Mt8g107640 PEVNREMAENDAKVLYKAGEKKLGTDEKTFVQIFSQRSAAQLAAINHFYHANYGHSLKKAIKNETSGNFAHAL

Gm07g12030 PEVNREIAQKDAKALYKAGEKKLGTDEKTFIHIFSERSAAHLAAVSSYYHDMYGHSLKKAVKNETSGAFEHAL

Gm09g30190 PEVNREIAQKDAKGLYKAGEKKLGTDEKTFIHIFSERSAAHLAAVSSYYHDMYGHSLKKAVKNETSGAFEHAL

Pt15g04350 REVDREMVVKDAKALYKAGEKKWGTDEKTFIHIFSERSAAHLAAVDSAYHDMYGNSLNKVIKKETSGHFEHAL

Pt12g03690 REVDRNMVEKDAKALYKAGEKRLGTDEMTFIRVFSERSAAHLAAVDSAYHNMYGNSLKKAIKKETSGHFEHAL

Cp00003g03400 PEVGREEAVKDAKALFKAGEKKWGTDEKTFIRIFSERSRAHMVAVDAAYHEMYGNSLKKAVKKETSGMFEFGL

Gm07g28080 LELDHIIVQEDAKQLYKSGEKRIGTDEKMFIKIFSEKSGAHLAAVNSTYIASYGHSLEKAIKKETSGNFESAL

Gm20g01460 PELDHIIVQEDAKQLYKSGEKRIGTDEKMFIKIFSEKSSTHLAAVNSAYIASYGHSLEKAIKKETSGSFGSAL

Cp00002g01210 PEIDNVLVEADATTLHTAISKKHGGEDKVFIQIFSERSKAHLAALGSAYRKMYGKFLGKAIRHETSGNFEHAL

At1g68090 PEIDNASVENDARTLKSAVARKHKSDDQTLIQIFTDRSRTHLVAVRSTYRSMYGKELGKAIRDETRGNFEHVL

Pt10g10090 PEIERVLVEEDAIAISKIEVKKSGMDESTFIQIFTERSSAHLAALASAYHKMFRKELRKTIKRETSGNFKYAL

Pt08g13700 PEIDSVLVEDDAKAINKIGVKKSGMDESTFIQIFTERSSAHLIALASVYHKMFGKELRKTIKREASGNFKYAL

Sm271856 PHVDMHLADADARELYRAGEGRLGTDESTFIRVFSTRSAAQLHAAFAAYKHLYKRDIDKAIKRETSGDFEDAL

Sm167346 PHVDMHLADADARELYRAGEGRVGTDESTFIRVFSTRSAAQLHAAFAAYKHLYKRDIDKAIKRETSGDFEDAL

Sm124402 PHVDMHLADADARELYRAGEGRLGTDESTFIRIFSTRSAAQLHAAFAAYKHLYKRDIDKAIRRETSGDFEDAL

Sm94768 PHVDMHLADADARELYRAGEGRLGTDESTFIRIFSTRSAAQLHAAFAAYKHLYKRDIDKAIKRETSGDFENAL

Sm227533 PHVDMHLADADARELYRAGEGRLGTDESTFIRIFSTRSAAQLHAAFAAYKHLYKRDIDKAIRRETSGDFEDAL

Pp1s219_3V6 FSVDSNLALADAHDLYRAGEARLGTNEDIIIHILTTRSPAQLNLALQYYRQTYGHEFMKAVKSETSGHFEAAI

Pp1s37_276V6 LAVDPNFALADAHALYQAGEARLGTDEDTFIHILTTRSPAQLNMTLQYYRQIYGRDFEKSIKRETSGHFEDAL

Pp1s6_292V6 TSVDRHIALNDAHQLNKVFTGKV-GDEDTLIRIFCTRSAQQLTATLNYYHQHYGHDFEESLINENSGDFEQAL

Pp1s102_141V6 TPVDRHIALNDAHQLHKVVIGKG-GNEDTLVRILCTRSIQQLTATFNYYHQHYGRELEQSLTRGGCGEFEQAL

Pp1s1_594V6 STVDRHIALNDAHQLNKVFTIVG-GNEDTLIRIFCTRSAQQLTATLNYYHQHYGHDFEQSLTRENSGEFEQAL

Pp1s61_299V6 TTVDRHIALTDAHQLNKVCSGKL-GNEETLIRIFSTRSPYQLTATMNFYEQHYGHDFEKALSKKDAGEFLQAL

Pp1s38_63V6 TAVDRHFALSDAHHLNKVCTGKI-GNEEMLIRIFTTRSSYQLSATMNYYQQHYGHDFEKVLSKQGSGEFLQAL

Ot11g03220 --LLTPREEQIAIYLQEAFGMWA-NDDWGLISMLVHRTEEEKELIRTKYTEHTGGDLIADIRSKCSGDYEDA-

MRCC299_56760 -IETDPDYDADIQILHDAVEGLG-TDEDAIIGVLRNKTEEQLQMLQRKYDATHCEDLKLRLKSETTGLFESER

PsABK21977 RDVNICQAKCDAKRLYGAREGRIGIDEGAIVKLLSDRNLNHLRAAFGYYKQFYGHDILKALRRETSGKFEYAL

PsACN40166 RDVNICQAKCDAKRLYGAREGRIGIDEGAIVKLLSDRNLNHLRAAFGYYKQFYGHDIL---------------

Cp00213g00130 EEVNMTLAKNEAKTLHEKISDKA-YNDEEVIRIISTRSKAQLNATLNHYNNGYGNAINKDLKSDPKDEYLQLL

Bd3g58830 PEVNTSLAHSEAKILHEKINDGA-YGDDEIIRILTTRSKAQLLATFNSYNDQFSHPITKDLKADPKDEFQATL

Os02g51750 PEVNTSLAHSEAKILHEKIHDKA-YSDDEIIRILTTRSKAQLLATFNSYNDQFGHPITKDLKADPKDEFLGTL

Sb04g027590 PEVNTSLAHSEAKILHEKIDKKA-YSDEEIIRILTTRSKAQLLATFNNYKDQFGHAINKDLKADPKDEFLSTL

Zm05g40790 PEVNTSLAHSEAKILHEKIHKKA-YSDEEIIRILTTRSKAQLLATFNSYKDQFTHAINKDLKADPKDEFLSTL

Bd1g45487 PEVNTRLAHSEAKILHEKIEHKA-YGDDEIIRILTTRSKAQLLATFNHYNDAFGHPITKDLKADPKDEFLKTL

Sb10g007760 PEVNTRLAHSEAKLLHEKIHHKA-YSDDEIIRILTTRSKPQLLATFNHYNDAFGHRINKDLKADPKDEYLKTL

Os06g11800 PEVNTKLAHSEAKILHEKIQHKA-YGDDEIIRILTTRSKAQLIATFNRYNDEYGHPINKDLKADPKDEFLSTL

Zm06g16450 PEVNTRLAHSEAKLLHEKIHHKA-YSDDEIIRILTTRSKPQLIATFNHYNDAFGHRINKLLYKIVQDVYLSCL

Cs273000 DEVNKTLAKSEAKILHEKIAGKE-YNHDEVIRILTTRSKAQLLATLNHYNNEYGNAINKDLKADPNDEYLKLL

At5g65020 DDVNMMLARSEAKILHEKVSEKS-YSDDDFIRILTTRSKAQLGATLNHYNNEYGNAINKNLKEESDDNYMKLL

Gm13g01870 DEVNLTLAKTEAKLLHEKISNKA-YNDDDFIRILATRSRAQINATLNHYKDAFGQDINKDLKADPKDEFLSLL

Mt5g063670 DEVNLTIAKAEAKILHEKISKKA-YNDDDFIRILATRSKAQINATLNHYKDAFGKDINKDLKEDPKNEYLSLL

Vv18g03470 EEVNMTLAKSEAKILHEKISEKA-YNHEDVIRILATRSKAQINATLNHYKNEFGNDINKDLKTDPKDEFLAIL

Pt02g09420 DEVNMTLAKSEAKMLHEKISNKA-YSDEELIRILATRSKAQINATLNQYKNEFGNDINKDLKADPNDEFLALL

Pt07g05300 EEVNTILAKSEAKILHEKISDKA-YSDEEIIRILTTRSKAQLNATLNHYNNAFGNAINKNLKEEADNDFLKLL

Pt05g07550 EEVNTMLAKSEAKILHEKISDKA-YSDDEIIRILTTRSKAQLNATLNHYNNAFGNAINKNLKEDADNEFLKLL

Gm05g31250 DEVNMTLAKSEAKLLHEKIAEKA-YNDEELIRILSTRSKAQLTATLNQYNNEFGNAINKDLKTDPKDEYLQLL

Gm08g14460 DEVNMTLAKSEAKLLHQKIAEKA-YNDEDLIRILSTRSKAQLTATLNQYNNEFGNAINKDLKTDPKDEYLQLL

At1g35720 DEVNMTLAKQEAKLVHEKIKDKH-YNDEDVIRILSTRSKAQINATFNRYQDDHGEEILKSLEEGDDDDFLALL

At5g10230 DEVNMTLARSEAKILHEKIKEKA-YADDDLIRILTTRSKAQISATLNHYKNNFGTSMSKYLKEDSENEYIQLL

At5g10220 ADVNVKLARSEAKTLHKKITEKA-YTDEDLIRILTTRSKAQINATLNHFKDKFGSSINKFLKEDSNDDYVQLL

Cp00036g01250 DE-----------KIHQKISEKA-YDSDDLVRILATRSKVQIIATLNRYKKEFGNDVNKDLKTDPNEEFLALL

Cs217870 PEVNATLATSEAKILHDKITEKA-YNDEELIRIISTRSKAQLNATFNHYNDQFGNAISKDLKTDPNDNYLKLL

Cp00042g00660 -----------------------------------------------------------ELKTDPKDEYLKLL

PsABK22223 PEVDMRLAKSEAKQLHEAIEDKA-FGNEEFIRIITTRSKAQLNATFNNYKDEYGHHINKDLKNEKPEEFLESL

Zm03g04200 ADVDMELARSEAKAVHEAVRD--G-GHEELIRVVGTRSKAQLRATFGCFKDEHRRSVAKALPRGTDPTGYLRL

Bd2g13620 EEVDGGLARSEAELIHEAVKN--GTDDGELIRILGTRSKAQLGATFSCFRDEHGTTLTK--------------

Os01g31270 DEVNDALAKSEAKILHETVTNGD-TDHGELIRIVGTRSRAQLNATFSWFRDERGTSITKALQHGADPTGYSHL

Zm08g03950 DDVDAELARSEAKIVHEAVRN--SHDHEELIRVLGTRSKAQLRATFSCFKDEHRRSVTKALPRGADDPGYLRL

Sb03g004990 DDVDMELARSEAKIVHEAVRN--GAGGHELIRVVGTRSKAQLRATFACFKDEHRSSVTKALPRGDDPTGYPRL

Gm13g26960 DEVNAKLAKTEADILHESIKEKK-GNHEEAIRILTTRSKTQLLATFNRYRDDHGASITKKLLDNASTDFQKAL

Gm15g38010 DEINAKLAKTEADILHESIKEKK-GNHEEAIRILTTRSKTQLLATFNRYRDDHGASITKKLLDNASTDFQKAL

Cs308090 ADVDLSLAKSEAERLERAIKDKT-FYHEDVVRILTTRSRPQLVATFNHYKDAYGISISEQLSSDKAGKFTEAL

Cs308080 GEIDAKLAKSEAEILERAVKDKA-FNHEDVIRILTTRSKAQLIATFNHYKDANGISISKQLGQDRDANFTEAL

Gm13g26990 DEINPKLAQSEAEILHDAVKEKK-GSYEETIRVLTTRSRTQLVATFNRYREIHGTSISKKLVDEGSDEFQRAL

Gm15g38040 DEINPKLAQTEAEILHDAVKEKK-GSYEETIRVLTTRSRTQLVATFNCYREIHGTSISKKLVDEGSDEFQRAL

Mt8g038210 DEINPKLAQTEAGILHESVKEKK-GSHEEAIRILTTRSKTQLIATFNRYRETHGTSITKKLLDEGSDEFQKAL

Mt8g038220 AEINPKLAKTEADILHESIKEKK-GNHEEAIRILTTRSKTQLLATFNRYRDDHGISITKKLLDNASDDFHKAL

Gm11g21480 DEINVKFSQTEANVLHESVKEKK-GNSEEVIRILTTRSKTQLVATFNRYRDEHGISISKKLLDQTSDDFHKVL

Mt3g018780 DEINAKLAQTEANIIHESVKEKK-GNNEEVIRILTTRSKTQLVATFNRYRDEHGISISKKLLDQTSDDFQKTL

Gm04g27100 SEINAKLAQSEADALHEAIKNKN-KSNDEIIRILTTRSKTQLVATFNRYRDDHGIAITKKLSDEGSDEFHKAA

Mt8g038180 VEINPILAKHEADILHEAVKNKK-GNIEEVIRILITRSKTQLKATFNRYRDDHGFSISKKLLNEASDDFLKAV

Cp04842g00010 TEVDGRLANSEADILRDVIKDKE-YNHEEVVRIVSTRSKPQLLATFNRYREEPGTSITKELLGESDNELAVLL

Cp36671g00010 AETHGRLANSEAEILRDAIIDKE-YNHEEVIRIVSTRSKLQLLATFNRYREEHGTSITKELLDDSDNEFAMVL

Pt03g19020 DEINTRLTNSEADILHDAIKDKA-FNHEDVIRILTTRSKAQLMATFNRYRDDHGSSITKDLLDEPADEFKTVL

Mt3g018920 SEVNASLAQCEADMLHEAIKHKN-HNHEEVIRILTTRSKTQLVATFNCYR-----HFLKKLSDEGSDGFHKAV

Mt3g018790 SEVNASLAQCEADMLHEAIKNKN-YNHEEVIRILTTRSKTQLVATFNCYRHDHGIAITKKLSDEGSDGFHKAV

Cp00197g00010 NEINDKLASSEADILRDAIKDKV-YNHDEVVRIISTRSKAQLSATFNCHKEKQGAFISEELLGDLHNDLAVML

Cp00157g00670 SEVDEAVAHAEAITLHDEIQRNP-LKHEEIIRVLGTRSKAQLNATFNIYKDIYGTSITKNLLGDPGNEYLAVL

Cs138380 NEIDENMAELEANIIDDEIKGKG-LKNNEMIRIVSTRSKPQLHATFNRYRDIHATSITKGLIGDSSDEYLAAL

Pt01g27650 HEVDEDLAISEAGLLHDDVYGKA-FNHDELVRVLTTRSKAQLNATFNRYQDIHGKSITKGLLGDPIDEYLGAL

At5g12380 EEIDEMLAQSEAAILHDEILGKA-VDHEETIRVLSTRSSMQLSAIFNRYKDIYGTSITKDLLNHPTNEYLSAL

Vv06g10680 EEIDEGVAHSEANILGDEMQGGA-LKGEEIIRILSTRSKAQLIATFNNYKQIHGTSITKSLRGDPTEEFSAAL

Gm11g21460 SEINAKLAQSEDDALHEAIKNKNKIATGMIMALPSLRVVSFLTCSFLPWQKLFDE---------GSDEFHKAA

Gm13g27000 -------QNLKLIFFMRPSKT--KHHEEVIGGSLVQEARPNLWQLSTASK--MKITFLSKLLEETSDDFYKAV

Gm15g14350 DEFDENLAHLEANILHQVIENKA-FNDDEIIRILCTRSKKQLCATFSTFRNVYGTTITKGLSTNPNDEYMTAL

Os05g31750 PRVSEETARAEAKALVAAVKS--AVENDDVVRILTTRSKPHLVETFKHYKEIHGRHIEEDLGHEETL------

Bd2g26760 PKVNEDTAKAEAKALGAALKK--KVENGEVVRILTTRSKPHLVETFKHYKELHGKHIHEDLGSEETL-----I

Zm06g23270 SK------------------------------------APLLLT-----------------------------

Bd1g62130 ARVSEDLATEEANAISAKP-----GNNEVLARVLATRSKPQLRATFRIYREIHGKPLEEDLIAVGGIC----L

Zm01g15800 ARVNEDLATEEAKALAAAVRA--AVQNEQVVRVLVTRSKPQLGATFRVYMELHGKPLEEELPAEPCL------

Sb01g035050 ARVSEDLATEEAKALAAAVRA--AVQNEQVVRVLATRSKPQLRATFRVYMELHGKPLEEDLAAEPCL------

Vv00g25070 PKVNEEIAKSEAKTLFAAIKN--AIEDEEVVRILTTRSKPHLKAIFKHYKEINGKNIDEDLDDELSL------

Cs308100 PKYKEEIAKSEAKKFAHSIKE--AIEDEEIVRILSTRSKHFLHALHKHYNISAGRSIDEDLHGDLRL------

Gm13g27010 TKVKDDTAKSEAKTLSNAIKN--ANEDDEVIRILATRSKLHIQAVCKHYKEISGKNLDEDLDDLRF-------

Gm15g38060 TKVKDDTAKSEAKILSNAIKN--ANEDDEVIRILATRSKLHLQAVYKHYKEISGKNLDEDLDDLRF-------

Mt8g038170 TKVKDDTAKSEAKTLSNAIKN--AVEDDEVIRILATRSKLHLQAVYKHYKEISGKNLEEDLNDLRF-------

Pt01g06030_ PKVKEDAAKSEAKILANAIKN--GIEDEEVIRILSTRSKAHLKVVYKHYKEVSGNNIHEDLDASDLIL-----

At2g38750 NKVKDDSAKSDAKILAEAVAS--SVEKDEVVRILTTRSKLHLQHLYKHFNEIKGSDLLGGVSKSSLL------

Sb09g018980 ELVDDELARAEAAELHDAVVA--RPLHGDVVRIVSSRSKPQLKATFERYRQGHGKAIDEVLEEERRSDLAAVL

Bd2g26770 EHVDDELARAEAAELHGAVVA--QPLHGDVVRVISSRSKPQLKATFQHYKQHHGKSFDEVL-EGNRNDLSAML

Zm06g23280 ELVDDELARAEAAELHDAVVA--RPLHGDVVRVVSSRSKAQLKATFERYRLDHGKAVDEVL-EERRSDLAAVL

Zm08g13570 DLVDDELARAEAAELHDAVVA--RLLHGQVVRIVSSRSKQQLQATFERYRQDRGKAFDEVL-EERRSDLAAML

Os05g31760 GGVDGELAIAEAAELHDAVVG--RALHGDVVRIVGTRSKAQLAVTLERYRQEHGKGIDEVL-DGRRGDLAAVL

Bd1g62120 EAVDVGIARLEAAQLAEAIRK--KPHADEVVRIVSTRSKAQLRATFQCYKQDHGSYIEE-DINNCSSSFARML

Sb01g035040 ERVDEDVARMEAAQLAEAIRK--RPHGDEVARIVSTRSKHQLRATFQLYKQEHGTDVDE-DITKHSSSFAKIL

Sb02g041850 EHVDMDVAKLEAAQLSEAIRE--KLHGDEVARIISTRSKPQLRATFQQYKDDQGTDIVEDIGSSNCCGLAGML

Bd1g18990 DQVDEATAAAEAALLCEAVRR--KHGED-VVRVISTRSKAQLAATFGLYRAHHGTELVEDIESRCSSQFAGAL

Os07g46550 DEVDMDVVRMEASQLAEAIKK--KRGEDEVVRIVTTRSKSQLRATFQRYREDHGSDIAEDIDSHCIGQFGRML

Pt01g06020 ELVDTKVANAEAAKLHEVIKSKK-LDQDDIILILSTRNFHQLRATFACYNQNFGNSIDQDIKSCGKGDLESLL

Gm15g38070 VAVNLEVAKEEASKLHEAINSKQ-LDNDHIIWILSTRNLFQLRETFACYNNLYGNTLEQDIKKCGNGDLESLL

Gm13g27020 VAVNLEVAKEEASKLHEAINCKQ-LEDDHIIWILSTRNFFQLRETFACYNNLYGNTLEQDIK-CGNGDLESLL

Mt8g038150 VTVNLEVAKSEAEKLHEAINNNK-LDDDHFVWILSTRNVFQIRETFASYKQLYGKTFEEDIKTCGKGDLTSLL

Vv00g25060 EMVDLNVAKLEAAKLHEAIKKKQ-LDHDDVVWILSTRNVFQLQATFEFYKQNYGNSIDQDIKSFGIGDLASLL

Cp00197g00020 ELVDPNVANSEAVLLHEAIQKKK-LDLDHVLYILGTRNFYQLRETFKSYKEKFKNPVEKDIKNCGNGDLESLL

At2g38760 DRTDAEVATIEAAMLREAIEKKQ-LDHDHVLYILGTRSIYQLRETFVAYKKNYGVTIDKDVDGCPGDALRSLL

Vv00g00800 ELVDLNVAKSEATKLHEAIEKKQ-LDRDEVMWILSTRNFFQLRATFKHYKQNYQVPIYQAIMSSGSDDLGSLL

Vv03g02080 ELVDFNLAKSEADKIHEAIEKNQ-LDHDDVVWILTTRNFFQLRATFVCYKQSYEVAIDQAINSSGNGDLGSIL

Vv00g00650 ELVDFNLAKSEATKLHEAIEKKQ-LDHDDVVWIMTTRNFFQLRATFVCYKQSYEVAIDQAINSSGNGDLGSIL

Vv00g00720 ELVDFNLAKFEAAKLHEAIEKKQ-LDHDDVVWILTTKNFFQLRATFVCYKQSYEVAIDQAINSSGNGDLGSIL

Vv00g00760 ELVDFNLAKSEVAKLHEAIEKNQ-LDHDDVVWILTTRNFFQLKATFVCYKQSYEVAIDQAINSSGNGDLGSIL

Vv00g00750 KLVDLNVAKSEAAKLHEAIEKKQ-SDRDEVMWILSTRNFFQLRATFKHYK-----QNYQAIMSSGSDDLGSLL

Vv00g00710 ELVDLNVAKSEAAKLHEAIEKKQ-LDRDEVMWILSTRNFFQLRATFKHYKQNYQVPIYQAIMSSGSDDLGSLL

Vv00g00660 ELVDLNVAKSEAAKLHEAIEKKQ-LDGDEVMWILSTRNFFQLRATFKHYKQNYQVPIYQAIMSSGSDDLGSLL

Cs307980 NKEEEKIVMLDAETLAKAFND--KIENREIINILMYRSISHLRAVFEQCK-----------------------

Cs307970 IKGEEKCLRKDITTLQNATSG--EICIKHIVSILTQRSIGHLRNMYRFCQPEMRRQPKSSLWI----------

**Figure S2D**

Bd4g31920 KCIYSPSKYYCKLLQKSMQRPESNKRLVTRAILGSDDVGMDKIKLAFKSNFGRNLGDFIHESLQSDYRDFLW

Os09g27990 KCIYNPSMYFSKLLHRSLQCSATNKRLVTRAILGSDDVDMDKIKSVFKSSYGKDLEDFILESLENDYRDFLL

Zm02g31380 KCIYNPSKYYSKLLHRSMLPAATDTRMVTRAILGSDDVGIDEIRSAFQSSYGKSLAEYIQENLGSDYRDFLV

Sb02g026390 KCIYNPSKYYSKLLQRSMLSAATDKRMVTRAILGSDDVGIDEIRSAFKSSYGRNLADYIQENLESDYKDFLV

Cs340270 KCIRNPPKYFAKVLYKSIKG-GESDGALKRVMLSRAEVDLDEIQRAFKGRYGVQLTDAICERTCDDYRDFFV

Pt13g04990 KCMCNPPTYYAKVLYTSIKGTTADNGALARVMISRAEVDLYEIRSIFKRKYGMELKDAICERISGDYRDFLA

Cp00161g00040 KCMCNPLSYYAKELHGGIKGRGERGSSLTRVMVSRAEVDMNEIQKVFKKKYGVELRDSICESLSGDYTDFLL

Vv08g00710 KCMCSPAKYYAKTLHSSIKGSATDKGALAWVMASRAGVDVDELVRVFRKKYGMELKEAIYGSISGDLRD---

Gm13g26040 KCICNPAHYYAKILYSSIKGETRDRRVLARTLVSRAEIDIDEIRRVFKEKYGKELADAICEGFSGDYYRDFL

Os09g20330 RCAQLPERHFARAVRAALERAGADRRDARRRRVDAGERRPERVRQRRHREVGRRLDRRLARGV---------

Cp00042g00810 RCIQNPGKFFAKQLRMKS---GDGRELLIRVVVTRSGIDIKDINNAFTAKTGSSLENLVRREFNGIVAAILT

Os09g23160 RCAENPAKYFAKVLRKSMKGMGTDDSTLIRVVVTRTEIDMQYIKAEYYKKYKKSLAEAIHSETSGNYRTFLL

Bd4g29680 RCAENPAKYFAKVLRKSMKGLGTDDKTLIRVVVTRTEIDMQYIKAEYYKKYKKPLGDAIHSETSGGYRTFLL

Sb02g024090 RCAENPAKYFAKLLRKAMKGLGTDEKTLTRVVVTRTEIDMQYIKAEYFKKYKKPLAEAINSETSGNYRAFLL

Zm02g30240 RCAENPAKYFAKLLRKAMKGLGTDDMTLIRVVVTRTEIDMQYIKAEYLKKYKKPLAEAINSETSGNYRTFLL

Zm07g13390 RCAENPAKYFAKLLRKAMKGLGTDDKTLIRVVVTRTEIDMQYIKAEYFKKYKKPLAEAIHSETSGNYRTFLL

Bd3g36240 RCAESPAKYFAKVMHKAMKGLGTSDTTLIRVVVTRTEIDMQYIKAEYHKKYKRSLADAIHSETSGNYRTFLL

Zm04g13650 RCADSPARYFAKELHRAMKGLGTSDSVLIRVVVTRAEIDMQYIKAEYHSMYKRSLADAIHAETSGNYRTFLL

Sb07g020760 RCADSPARYFAGVLHKAMKGLGTSDSTLIRVVVTRAEIDMQYIKAEYHRMYKRSLADAIHAETSGNYRTFLL

Os08g32970 RCAESPAKYFAKVLHEAMKGLGTNDTTLIRVVTTRAEVDMQYIKAEYHRSYKRSLADAVHSETSGNYRTFLL

Cs234810 RCAENPALYFAKVLHKAMKGMGTDDSTLIRIIVTRTEIDMQYIKTEYQKKYKKTLHDAVHSETSGSYRDFLL

Cs234800 LCAENPGFYFAKVLRKAMKGMGTDDSTLIRVIVSRAEIDMQYIKAEYHKKYKKTLNKAVQSETSGSYKDFLL

Vv01g05380 QSAENSGKYFAKVLHKAMKGLGTDDTTLTRIIVTRAEIDLQYIKQEYRKKYGKTLNDAVHSETSGHYKAFLL

Gm08g06100 QCAENPAKYFAKVLRKAMKGLGTDDTKLIRVIVTRAEIDLQYIKAEYLKKYKKTLNDAVHSETSGHYRAFLL

Mt8g107640 QCAESPAKYFAKVLRKAMKGLGTDDTKLMRVIVTRSEIDLHYIKAEYLKKYKKTLNDAVHSETSGHYRAFLL

Gm07g12030 QCAVNPGKYFAKVLRKAMKGLGTDDSTLIRVIVTRTEVDMQYIKAAYLKKHKKTLNDEVHSETSGHYRTFLL

Gm09g30190 QCAVNPGKYFAKVLHKAMKGLGTDDSTLIRVVVTRTEVDMQYIKAAYLKKHKKTLNDEVHSETSSHYRTFLL

Pt15g04350 LCSENPANYFAKVLHKAMKGMGTNDTALIRVIVTRTEIDMHYIKAEYLKKYKKTLNDAVHSETSGNYQAFLL

Pt12g03690 QCSENPAKYFVKLLRKAMKGLGTNDTALIRVIVTRTEIDMQYIKAEYLKKYRKTLNDAVHSETSGHYRAFLL

Cp00003g03400 QCAHNPAKFFAKELYKAMKGLGTNDTTLIRIIVTRTEIDMQYIAAEYFKKYKETLNEAVHSETSGHYRTFLL

Gm07g28080 RCATDPAMYFAKILRKSMKGVGTDDSRLIRVIVTRTEIDMQFIKIAYYKKYGKPLTHAVKSDTSGHYKDLLL

Gm20g01460 RCATDPAMYFAKILRKSMKGVGTDDSRLIRVIVTRTEIDMHYIKITYYKKYGKPLTHAVKSDTSGHYKDFLL

Cp00002g01210 QCSENPAFYFAKVLRKAMKGLGTDDTTLIRIIMTRVEVDMKYIKAEYRKKYGKTLNDAVHSDTSGHYRTFLL

At1g68090 QCAENSCFYFAKALRKSMKGLGTDDTALIRIVVTRAEVDMQFIITEYRKRYKKTLYNAVHSDTTSHYRTFLL

Pt10g10090 EYAVDPTKHYATMLRKAMKGLGTDDSTLIRILATRAEIDLQKIKEDYLKRYKRPLVEVVHSDTSGYYRAFLL

Pt08g13700 QYAVDPTKHYATVLRKATKGLGTDDSTLIRILVTRAEIDLQRIEEEFLKKYKRPLPEVVHSETSGHYRAFLL

Sm271856 KSVTRPGRYFAKVLYDSMKRMGTDDSTLIRVVVTRAEQDMQYIKADFYQKYKKPLESMISGDTSGNYKHFLL

Sm167346 KSATRPGRYFAKVLYDSMKRMGTDDSTLIRVVVTRAEQDMQYIKADFYQKYKKPLESMISGDTSGNYKHFLL

Sm124402 KSVTRPGRYFAKVLYGSMKRMGTDDSTLIRVVVTRAEQDMQYIKADFYQKYKKPLESMISGDTSGNYRHFLL

Sm94768 KSATRPGRYFARVLYDSMKGMGTDDSTLIRVVVTRAEQDMQYIKADFYQKYKKPLESMISVDTSGNYKHFLL

Sm227533 KSVTRPGRYFAKVLYDSMKRMGTDDSTLIRVVVTRAEQDMQYIKADFYQKYKKPLESMISGDTSGNYRHFLL

Pp1s219_3V6 QCTCNPAKFFAQELHDAMKGYGTKDADLMRVITTRAEIDMYYIKQEFQAMFKKTLQEAIQSNTSGDYRHFLL

Pp1s37_276V6 QCTCYPARYFAQELYSSMKGLGTKDRDLIRIITTRAEIDMYYIKQEFQIMYGTTLEYMIAGDTSGDYRYFLL

Pp1s6_292V6 MCFRQPAKFYAEELHTALGGAGTDDDALIRVITTRAEVDMQYIKLEFANECKRSLEEMIANDTIGNYRYFLL

Pp1s102_141V6 MCYRQPAKFYAEELNAALGGAGTDDDALIRVVTTRAEVDMQYIKLEFANESKKKLEDMIANETSGNYRYFLL

Pp1s1_594V6 ICFRQPAKFYAEELCNALGAAGTDDDALIRVVTTRAEVDMQYIKLEFTNLSKRTLEEMVANDTAGTYRYFLL

Pp1s61_299V6 QCLRRPSEFYAEDIITALSKGNGDEDTLVQIITTRADVDMHMIRIEFMKECKRALEQVISERAMGVIGQFLV

Pp1s38_63V6 QSLRQPSKFYAEELSDALSGIGTDEETLVLIITTRAEVDMQFIKLEFMNECKRSLEDVVRDETIGKLRQLLL

Ot11g03220 ACISPKERTIARGMRQCIAGWFSTNKTGIMALLTHKDMVMPRLRKEFEKEFRQTLQNCIKKECAGEFEAALV

MRCC299_56760 GLLTNREEQIAIYLKEAFEGWFSDDWGLISMLVHRTPQEMELIRNAYTRVHGRDLIADIRKNCKGDYEKALV

PsABK21977 KCICYLAKYFSKVLRISLDQ--SEYAALTRVMVTRAEVDMEEIKATYREKYGISLEQAICKQTSGSYRDFLL

PsACN40166 -----------KVLRISLDQ--SEYAALTRVMVTRAEVDMEEIKATYREKYGISLEQAICKQTSGSYRDFLL

Cp00213g00130 KCLTCPEKYFEKLLRLAINKMGTDEWALTRVITTRAEIDMQRIKEEYQRRNSVPLDRAIAKDTTGNYEKMLL

Bd3g58830 RCFTCPDRYFEKIIRLALGGVGTDENSLTRIITTRAEVDLKLIKEAYQKRNSVPLEKAVSKDTTRDYEDMLL

Os02g51750 RCFTCPDRYFEKVIRLALGGMGTDENSLTRIITTRAEVDLKLIKEAYQKRNSVPLERAVAKDTTRDYEDILL

Sb04g027590 RCFTCPDRYFEKVIRLALGGVGTDEDALTRVITTRAEVDLKLIGEAYQKRNSVPLDRAVAKDTTRDYEDILL

Zm05g40790 RCFTCPDRYFEKVIRLALGGMGTDEDDLTRVVTTRAEVDLKLIKEAYQKRNSVPLERAVAKDTTRDYEDIML

Bd1g45487 RCFTCPDRYFEKVIRLAIAGTGTDENSLTRIITTRAEVDLKLIKEAYQKRNSVPLERAVAGDTSGDYESMLL

Sb10g007760 RCFSCPDRYFEKVARQAIAGLGTDENALTRVITTRAEVDLKLIKEAYQKRNSVPLERAVAGDTSGDYESMLL

Os06g11800 RCFCCPDRYFEKVIRLAIAGMGTDENSLTRIITTRAEVDLKLITEAYQKRNSVPLERAVAGDTSGDYERMLL

Zm06g16450 HCIHLPNL----------------------------------------------------------------

Cs273000 KSLTFPERHFAKILRLAINKLGTDEWALARVVASRAEIDMERIKEEYYRRNSVPLGRAIAKDTSGDYEKMLL

At5g65020 TCLTYPEKHFEKVLRLSINKMGTDEWGLTRVVTTRTEVDMERIKEEYQRRNSIPLDRAIAKDTSGDYEDMLV

Gm13g01870 KCLIRPEKYFEKVVRLAINKRGTDEGALTRVVATRAEVDLKNIADEYQRRSSVPLERAIVKDTTGDYEKMLV

Mt5g063670 KCLVFPERYFAKIIREAINKRGTDEGALTRVVATRAEIDLKIIAEEYQRRNSIPLDRAIVKDTTGDYEKMLL

Vv18g03470 KCLTRPEKYFEKVLRLAINKRGTDEGALTRVVTTRAEIDMKIIKEEYHKRNSVTLDHAIGKDTTGDYEKMLL

Pt02g09420 KCLTRPEKYFEKVLRLAINKRGTNEGALTRVVATRAEIDMKLIKDEYQRRNSIPLDRAIVKDTDGEYEKLLL

Pt07g05300 KCLTYPEKYFEKLLRLSIKKLGTDERALTRVVTTRAEVDMERIKEEYHRRNSVTLERDIAGDTSGDYERMLL

Pt05g07550 KCLTYPEKYFEKLLRLAIKKIGTDEGALTRVVTTRAEVDMERIKEEYHRRNSVTLDHDIAGEASGDYERMLL

Gm05g31250 KCLTYPEKYFAKVLRMAINKLGTDEGALTRVVTTRAEVDLQRIAEEYQRRNSIPLDRAIANDTSGDYQSILL

Gm08g14460 KCLTYPEKYFAKVLRLAINKLGTDEGALTRVVTTRAEVDLQRIAEEYQRRNSIPLDRAIASDTSGDYQSILL

At1g35720 QCLTRPELYFVDVLRSAINKTGTDEGALTRIVTTRAEIDLKVIGEEYQRRNSIPLEKAITKDTRGDYEKMLV

At5g10230 KCLTYPEKYFEKVLRQAINKLGTDEWGLTRVVTTRAEFDMERIKEEYIRRNSVPLDRAIAKDTHGDYEDILL

At5g10220 KCLTYPEKYFEKVLRRAINRMGTDEWALTRVVTTRAEVDLERIKEEYLRRNSVPLDRAIANDTSGDYKDMLL

Cp00036g01250 KCLTRPEKYFEKVLRLAINRQGTDEGALTRVVATRAEVDMKLITEEYQQRSSVPLDRAITKDTHGDYEKMLL

Cs217870 KCLTWSEKYFEKVLRLAIKGLGTDEEALTRVVVTRAEVDMKRIAEEYYRRNSVPLGQAIKGDTSGDYESMLL

Cp00042g00660 KCLTYPEKYFEEVLRLSVKGLGIDEEALTRVVVTRADVDMKHIKEEYYRRNSEPLDAAIKGDNSGNYERMLL

PsABK22223 KCICFPERYFAKVLRLAIDKLGTEEEALIRVVVTRAETDMNNIKEEYHKRTSKTLEHAIAADTSGYYEEFLL

Zm03g04200 RCVADPSKYFAKVLRSATREAGTDEDSLARVVLLHAEDDMGAICAAFLKRASCTLEQAVAKETSGDYRSFLL

Bd2g13620 ------------VLRNAMHEAGTDEDSLTRVVVTHAEKDLRDIKDVFRKTTSVALEQAIAKETSGDYKTFIV

Os01g31270 RCISDANKYFVKVLRNAMHKSGTNEDSLTRVIVLHAEKDLKGIKDAFQKRASVALEKAIGNDTSGDYKSFLM

Zm08g03950 RCVADPTKYFAKVLRNATREAGTDEDSLTRVVVLHAEDDMGAICGAFQKRASCTLQQAIAKETSGDYSSFLL

Sb03g004990 RCVADPSKYFAKVLRHATREAGTDEDSLTRVVVVHAEDDMGAICAAFQKRASCTLEQAIAKETSGDYRSFLL

Gm13g26960 RCINDHKKYYEKVLRNAIKGVGTDEDALTRVVVSRAEKDLRDIKELYYKRNSVHLEDAVAKEISGDYKKFIL

Gm15g38010 RCINDHKKYYEKVLRNALKNVGTDEDALTRVVVSRAEKDLRDIKERYYKRNSVHLEDAVAKEISGDYKKFIL

Cs308090 ECIDDPYQYYEKVVRNAIKRVGKDEDALTRVVVSRAEKDLRQIKEAYHKRNSVTLDDAVSKETSGDYKRFIL

Cs308080 RCINDPVKYYEKVVRNAIKKVGKDEDALTRVVVTRAEKDLRQIKEAYHKRNSVTLDDAVKKETSGDYERFIL

Gm13g26990 RAINDPIKYYEKVVRNAIKKVGTDEDALTRVVVSRAEKDLKIISEVYYKRNSVLLEHAIAKEISGDYKKFLL

Gm15g38040 RGIKDPIKYYEKVVRNAIKKVGTDEDALTRVVVSRAEKDLKIISEVYYKRNSVLLEHAIAKETSGDYKKFLL

Mt8g038210 RSFNDHVKYYEKVVRDAIKKVGTDEDALTRVIVSRAQHDLKVISDVYYKRNSVLLEHVVAKETSGDYKKFLL

Mt8g038220 RCINDHKKYYEKILRGALKRVGTDEDGLTRVVVTRAEKDLKDIKELYYKRNSVHLEDAVAKEISGDYKKFIL

Gm11g21480 RCINDHKKYYEKVLRNAVKKFGTDEDGLSRVIVTRAEKDLKDIKELYYKRNSVHLEDEVSKETSGDYKKFLL

Mt3g018780 RCINDHKKYYEKVLRNAIKKFGTDEDGLSRVIVTRAEKDLRDIKELYYKRNSVHLEDEVSKETSGDYKKFIL

Gm04g27100 SCINDHKKYYEKVLRNAMEHLGTAEDALTRVIVTRAEKDLKEIKEVYYKRNSVHLEHAVAKETSGDYKKFLL

Mt8g038180 RCIDDHKKYYEKVLRGALKRIGTDEDGLTRVVITRAEKDLKDIKELYYKRNSVHLEDTVAKEISGDYKKFLL

Cp04842g00010 RCLVDPIKYFEKVLRNSIKKIGTDEDALTRVIVTRAEKDLQDIKDLYYKKNSELLEHAVAKDTTGDYKHFLL

Cp36671g00010 RCLVDPKKYFEKVLRNSIKKLGTDEDALTRVIVTRAEVDLQDIKELYYKKNSELLEHAVAKDTSGDYKHFLL

Pt03g19020 RCLNDHKKYYEKILRNAIKKVGTDEDALTRVIVTRAEKDLNDIKEIYYKRNSVPLDQAVANDTSGDYKAFLL

Mt3g018920 SCINDHNKYYEKVLRNAMEIVGINEDALTRVIVTRAEKDLEDIKKVYYKRNSVQLEHAVAKKTSGDYKKFLL

Mt3g018790 SCINDHNKYYEKVLRNAMETVGTDEDALTRVIVTRAEKDLEDIKKVYYKRNSVQLEHAVAKKTSGDYKNFLR

Cp00197g00010 LCLIDPKEYFEWVLHKSMQGIGTDEDALTRVIITRAEKDLLYIKELYHKKNGKLLAHDVAGDTSRHYKHFLL

Cp00157g00670 RCIKNSKKYFAKVLRNAINTVGTDEDALSRVIVTRAEKDLKDIMELYLKRNNISLEQAVARDTSGDYKAFLL

Cs138380 RCIRDPKKYYAKVLRNAMNTDRVDKDGISRVIVTRAEKDLKEIMEMYLKRNNISLEEAVSREIGGDYKAFLL

Pt01g27650 RCIRDPRKYFVKVLRRAVHKEDTDEDALSRVIVTRAEKDLKEIKELYLKRNNISLDQAVAVDTHGEYKEFLL

At5g12380 RCIKNPTRYYAKVLRNSINTVGTDEDALNRVIVTRAEKDLTNITGLYFKRNNVSLDQAIAKETSGDYKAFLL

Vv06g10680 RCIRNPKKYLQKLLCNVINNMGTDEDTLSRVIITRAEKDLKEMKELYLERNSRSLEDAVSSETTGDYKAFLL

Gm11g21460 SCINDHKKYCQKVLCNAMEHVGTDEDALTRVIVTRAEKDLKEIKEMYYKRNIVHLEHVAAKET---------

Gm13g27000 HCINDHKKYYEKVLRNAIKGVGNNEDGQTRVFVTRAEKDLKDIKELYYKKNSVHLEDTMAKENSGYYKKFLL

Gm15g14350 RCIKNPRRYLAKVLCYALNELIAEEHELSRVIITRAERDLNEINDLYFKRNGVTLDSSVAKKTSGNYKNFLL

Os05g31750 LCLATPARYFSEVVAAAVSDGADAKEALTRVAVTRADVDMDAIRAAYHEQFGGRLEDAVAGKAHGYYRDALL

Bd2g26760 QCLAAPEMYFSQVMEAALREGADGKEALARVAVTRSDVDMDGIRAAYQEQFGARLEDAVAACAHGHFKDALL

Zm06g23270 ------------------------------------------------------------------------

Bd1g62130 RCLDAPAKYFGEVIAGAFKEGADAKAALTRVVVSRSEADMEEIKEAYVKQHGAKLVDAVAKNTHGHYRDALL

Zm01g15800 RCLDSPPKYFSEVIHRAFSDDADAKAALTRVLVSRADTDMEDIKDAYTRQYGTKLADAVAKNTHGHYKEALL

Sb01g035050 KCLDSPPRYFSEVISRAFRDDADAKAALTRVVVSRADTDMEDIKDAYARQYGAKLADAVAKNTHGHYKDALL

Vv00g25070 QCLCTPQTYFSKVLGAAFQNDADAKEALTRVIVTRADDDMKEIKEEYQKKYGVSLSKKIEDAVNGNYKDFLL

Cs308100 LCLTNPVKYFTQLLNVSLKADADIKKVLTRIVVTRADNDMKEIKVEFKKQFGISLAEKIGSVCNGSYKDFLI

Gm13g27010 QCLCTPQIYFSKVLNAALKIDVDTKKSLTRVIVTRADIDMKDIKADYHNLYGVSLPQKVEEVARGSYKDFLL

Gm15g38060 QCLCTPQTYFSKVLNAALRIDVDTKKSLTRVVVTRADIDMKDIKAEYHNLYGVSLPQKVEEVARGSYKDFLL

Mt8g038170 QCLCTPQVYFSKVLDAALKNDVNIKKSLTRVIVTRADIDMKEIKAEYNNLYGVSLPQKIEETAKGNYKDFLL

Pt01g06030_ ECLCTPHAYFSKVLDEAMSSDAHTKKGLTRVIVTRADVDMKEIKEEYMNLFGVSLSKKIEEKANGNYRDFLV

At2g38750 ICLLKPALYFSKILDASLNKDKTTKKWLTRVFVTRADDEMNEIKEEYNNLYGETLAQRIQEKIKGNYRDFLL

Sb09g018980 WCLTSPEKHFAEVIRSSIVGLGTDEESLTRAIVSRAEIDMKKVKEEYKARYRKTVTSDVNGDTSGYYNGILL

Bd2g26770 WCLTTPEKHFAEVIRNSIVGLGTDEESLTRGIVSRAEIDMKKVKEEYKARFKTTVTNDIIGDTSGYYKDILL

Zm06g23280 WCLTSPEKHFAEVIRSSIVGLGTDEESLTRAIVSRAEIDMKKVKEEYRARYRTTVTSDVNGDTSGYYNVILL

Zm08g13570 WCLTSPEKHFAEVIRRSIVGLGTDEESLTRVIVSRAEIDMKKVKEEYKVRYRTTVTSDVNGDTSGYYNSILL

Os05g31760 WCLTSPEKHFAEVIRTSILGLGTDEEMLTRGIVSRAEVDMEKVKEEYKVRYNTTVTADVRGDTSGYYMNTLL

Bd1g62120 WCLTSPEKHFAEVIRYSILGIGTDEDALTRAIVSRAEIDMEKIKQEYKVRLKSTVTNDVIGDTSGYYMDILL

Sb01g035040 WCLTSPEKHFAEAIRYSILGFGTDEDTLTRAIISGSEIGMNKIKEEYKVRFKTTVTSDVVGDTSGYYKDFLL

Sb02g041850 LCLASPEKHFAEVIRYSILGLGTYEDMLTRVIVSRAEVDMEQIKEEYRARYGSAVSLDVAGDTSFGYRDMLL

Bd1g18990 WCLTSPEKHFAEVIRNAVEGLGTYEDVLTRAVVSRAEVDMASVRAEYRARFGVTVASDIADDTSFGYRDVLL

Os07g46550 WCLTSPEKHFAEVIRHSILGLGTYEDMLTRVIVSRAEIDMRHIREEYKVRYKTTVTRDVVGDTSFGYKGFLL

Pt01g06020 KCIDTPEKHFAEVIGEAIIGFGTDEDSLTRAIVARAEIDTMKIRGEYFNIFKTNLDGAVTGDTSGDYKDFLM

Gm15g38070 WCIDCPEKHFAKVVRDSIVGFGTDEDSLNRAIVTRAEIDLLNVRFEYANVYKSSLDDDVIGDTSGYYKDFLM

Gm13g27020 WCIDCPEKHFAKVVRDSIVGFGTDEDSLNRAIVTRAEIDLLKVRFEYANVYKTSLDDDVIGDTSGNYRDFLM

Mt8g038150 WCIECPEKHFAKVIRDSIVGLGTDEDSLNRAIVTRAEIDLLKVRFEYANMYKSSLDDDVIGDTSGDYMEFLL

Vv00g25060 WCIDSPEKHFAEVIRASIVGLGTDEDSLTRAIVTRAEIDMMRSEG----IFQHKQDQPGQCS----------

Cp00197g00020 SCIDCPEKHFAEVIGTSVIGFGTDEDSLTRAIVSRAEIDMMKIRGEYFNLYKTNLDGAVIDDTSGDYRDFLM

At2g38760 FCIDTPEKHFAKVVRDSIEGFGTDEDSLTRAIVTRAEIDLMKVRGEYFNMYNTSMDNAITGDISGDYKDFII

Vv00g00800 LCIDAPEKHFAEVIRASLSGHRTDVHSLARAILARVEIDMMKIKEEYFNMNKVSLDDAVVGKTSGGYKDFLM

Vv03g02080 LCIVSPEKHFAEVIKASTVGYWTDEDSLTRAIVTRAEIDMTKIKGEYFKMNNTNLDDVVRRDTSGVYKSFLM

Vv00g00650 LCIVSPEKHFVEVIRASTIGYWTDEDSLTRAIVTRAEIDMTKIKEEYFKMNNTNLDDVVRRDASGVYKSFLM

Vv00g00720 WCIVSPEKHFAEVIKASTVGYWTDEDSLTRAIVTWAEIDMTKIKGDYFKMNNTNLDDVVRHDALGVYKSFLM

Vv00g00760 LCIVSPEKHFAEVIRASTVGYWTDEDSLTRAIVTQAEIDMTKIKGEYFKMNNTSLDDVVRRDASGVYKSFLM

Vv00g00750 LCIDAPEKHFAEVVEI--------------------------------------------------YK----

Vv00g00710 LCIDAPEKHFAEVIRASLSGHRTDVHSLARAILARVEIDMMKIKEEYFNMNKVSLDDAVVRKTSGGYKDFLT

Vv00g00660 LCIDAPEKHFAEVVEI--------------------------------------------------YK----

Cs307980 ------------------KIGVTPKDSLSRIIITCPKVDLDKIKIKFKETSKITLQERIRLVCKGSYKDLLL

Cs307970 LCLVDPIEYFYQVLSNSIDSSPSCLDSISRIIMTRRGVDLDEINTKFRMFDELSLQDRIKLYCKGTYQKLLL
