# Supplementary material for: Genome-wide Comparative Analysis of Annexin Superfamily in Plants
Source: PLoS One. 2012 Nov 2;7(11):e47801. doi: 10.1371/journal.pone.0047801 (PMC3487801; doi:10.1371/journal.pone.0047801)
Supplement: Table S1 — Summary of 149 annexin genes identified in Viridiplantae and their sequence features. (DOC) [file pone.0047801.s003.doc]

**Table S1 Summary of 149** annexin genes identified in Viridiplantae and their sequence features.

| **Gene ID** | **Chr**^a^ | **Str**^b^ | **Coordinates** | **Exons** | **AA**^c^ | **Protein ID** | **MW/p*I***^d^ | **Anx**  **repeats** | **Ca^+2^ sites** |
| --- | --- | --- | --- | --- | --- | --- | --- | --- | --- |
| ***Micromonas sp* RCC299** |  |  |  |  |  |  |  |  |  |
| MRCC299_03g01870 | 3 | + | 374641 - 377124 | 1 | 827 | 56760 | 93.1/5.73 | 5 | 3 |
| ***O. tauri*** |  |  |  |  |  |  |  |  |  |
| estExt_fgenesh1_pg.C_Chr_11.00010224 | 11 | + | 518457 - 519807 | 1 | 432 | 24272 | 47.7/5.78 | 3 | 2 |
| ***P. patens*** |  |  |  |  |  |  |  |  |  |
| Pp1s1_594V6 | Scaffold_1 | + | 3426862 -3430030 | 6 | 403 | Pp1s1_594V6 | 45.5/9.04 | 4 | 1 |
| Pp1s6_292V6 | Scaffold_6 | - | 2447137 - 2450077 | 6 | 396 | Pp1s6_292V6 | 45.0/8.52 | 4 | 1 |
| Pp1s37_276V6 | Scaffold_37 | - | 1894471 - 1897986 | 6 | 314 | Pp1s37_276V6 | 35.6/7.18 | 4 | 4 |
| Pp1s38_63V6 | Scaffold_38 | - | 326018 – 328763 | 6 | 395 | Pp1s38_63V6 | 44.3/8.83 | 4 | 1 |
| Pp1s61_299V6 | Scaffold_61 | + | 1644358 – 1647282 | 7 | 486 | Pp1s61_299V6 | 54.6/9.59 | 3 | 0 |
| Pp1s102_141V6 | Scaffold_102 | - | 924459 - 928362 | 7 | 395 | Pp1s102_141V6 | 44.3/8.98 | 3 | 1 |
| Pp1s219_3V6 | Scaffold_219 | + | 22582 - 25378 | 6 | 314 | Pp1s219_3V6 | 35.4/6.64 | 4 | 3 |
| ***S. mollendorffii*** |  |  |  |  |  |  |  |  |  |
| estExt_fgenesh1_kg.C_740007 | Scaffold_74 | + | 510715 - 512114 | 6 | 315 | 271856 | 36.1/9.00 | 4 | 4 |
| estExt_Genewise1Plus.C_50869 | Scaffold_5 | + | 2598850 - 2600250 | 6 | 315 | 167346 | 36.1/9.00 | 4 | 4 |
| e_gw1.74.90.1 | Scaffold_74 | - | 517917-519173 | 6 | 315 | 124402 | 36.1/9.29 | 4 | 4 |
| e_gw1.16.385.1 | Scaffold_16 | + | 1958131 - 1959401 | 6 | 315 | 94768 | 36.1/9.01 | 4 | 4 |
| fgenesh1_kg.C_scaffold_5000033 | Scaffold_5 | - | 2606098 - 2607448 | 6 | 315 | 227533 | 36.3/9.21 | 4 | 4 |
| ***P. sitchensis*** |  |  |  |  |  |  |  |  |  |
| PsABK22223 |  |  |  |  | 316 | ABK22223 | 36.8/6.2 | 4 | 2 |
| PsABK21977 |  |  |  |  | 320 | ABK21977 | 36.4/8.58 | 4 | 1 |
| PsACN40166 |  |  |  |  | 290 | ACN40166 | 32.8/7.50 | 3 | 0 |
| 1. ***thaliana*** |  |  |  |  |  |  |  |  |  |
| At1g35720 (*AnnAt1*) | 1 | + | 13225197 - 13227195 | 3 | 317 | At1g35720 | 36.2/5.01 | 4 | 2 |
| At5g65020 (*AnnAt2*) | 5 | + | 25973815 - 25975726 | 5 | 317 | At5g65020 | 36.2/5.96 | 4 | 2 |
| At2g38760 (*AnnAt3*) | 2 | + | 16200994 - 16202669 | 6 | 321 | At2g38760 | 36.2/6.38 | 4 | 2 |
| At2g38750 (*AnnAt4*) | 2 | - | 16196265 - 16198491 | 6 | 319 | At2g38750 | 36.2/7.41 | 2 | 0 |
| At1g68090 (*AnnAt5*) | 1 | - | 25519442 - 25520774 | 6 | 316 | At1g68090 | 36.0/9.92 | 4 | 2 |
| At5g10220 (*AnnAt6*) | 5 | - | 3206868 - 3208825 | 4 | 318 | At5g10220 | 36.5/8.19 | 4 | 2 |
| At5g10230 (*AnnAt7*) | 5 | - | 3209540 - 3211423 | 4 | 316 | At5g10230 | 36.5/7.67 | 4 | 2 |
| At5g12380 (*AnnAt8*) | 5 | + | 4009223 - 4010687 | 6 | 316 | At5g12380 | 35.7/6.79 | 4 | 2 |
| ***M. truncatula*** |  |  |  |  |  |  |  |  |  |
| Mt3g018780 | 3 | + | 4700877 - 4704245 | 6 | 314 | Medtr3g018780.1 | 36.1/7.83 | 4 | 2 |
| Mt3g018790 | 3 | + | 4705923 - 4712204 | 6 | 314 | Medtr3g018790.1 | 35.8/6.47 | 4 | 1 |
| Mt3g018920 | 3 | + | 4759547 - 4768903 | 7 | 212 | Medtr3g018920.1 | 24.5/8.97 | 1 | 0 |
| Mt5g063670 | 5 | - | 25678682 - 25680865 | 5 | 316 | Medtr5g063670.1 | 36.1/7.14 | 4 | 2 |
| Mt8g038150 | 8 | - | 8889398 - 8892684 | 6 | 321 | Medtr8g038150.1 | 36.4/5.66 | 4 | 2 |
| Mt8g038170 | 8 | + | 8900995 - 8904564 | 6 | 314 | Medtr8g038170.1 | 36.0/8.64 | 3 | 0 |
| Mt8g038180 | 8 | - | 8905276 - 8907453 | 6 | 314 | Medtr8g038180.1 | 36.0/8.80 | 4 | 2 |
| Mt8g038210 | 8 | - | 8919904 - 8924676 | 8 | 339 | Medtr8g038210.1 | 36.6/6.89 | 4 | 2 |
| Mt8g038220 | 8 | - | 8926944 - 8929302 | 8 | 373 | Medtr8g038220.1 | 42.3/8.89 | 4 | 2 |
| Mt8g107640 | 8 | - | 32404198 - 32405928 | 5 | 315 | Medtr8g107640.1 | 35.6/9.25 | 4 | 3 |
| ***P. trichocarpa*** |  |  |  |  |  |  |  |  |  |
| Pt01g06020 | 1 | - | 4794487 - 4797242 | 6 | 318 | 17345417 | 35.5/5.69 | 4 | 2 |
| Pt01g06030 | 1 | + | 4801792 - 4803976 | 6 | 312 | 17327588 | 35.6/8.18 | 3 | 0 |
| Pt01g27650 | 1 | + | 27208041 - 27209860 | 6 | 315 | 17321541 | 35.7/6.10 | 4 | 2 |
| Pt02g09420 | 2 | - | 6883569 - 6885764 | 5 | 316 | 17311529 | 35.9/6.15 | 4 | 2 |
| Pt03g19020 | 3 | + | 18610898 - 18612729 | 5 | 301 | 17302396 | 34.3/5.80 | 4 | 2 |
| Pt05g07550 | 5 | + | 5300168 - 5302388 | 5 | 316 | 17329741 | 36.0/6.18 | 4 | 2 |
| Pt07g05300 | 7 | + | 3575603 - 3577788 | 5 | 316 | 17311952 | 36.1/6.34 | 4 | 2 |
| Pt08g13700 | 8 | - | 9153139 - 9155200 | 6 | 316 | 17333691 | 35.7/9.18 | 4 | 3 |
| Pt10g10090 | 10 | + | 11547874 - 11549934 | 6 | 316 | 17328851 | 35.9/9.22 | 4 | 3 |
| Pt12g03690 | 12 | + | 3107454 - 3110224 | 6 | 316 | 17327501 | 35.7/9.23 | 4 | 3 |
| Pt13g04990 | 13 | - | 3663210 - 3664596 | 4 | 329 | 17324110 | 37.5/9.00 | 3 | 0 |
| Pt15g04350 | 15 | + | 5663783 - 5668049 | 6 | 316 | 17306301 | 35.6/8.55 | 4 | 3 |
| ***V. vinifera*** |  |  |  |  |  |  |  |  |  |
| Vv00g00650 | Chr Un | - | 2170761 - 2173434 | 6 | 321 | GSVIVG01000282001 | 36.7/8.52 | 4 | 0 |
| Vv00g00660 | Chr Un | - | 2246316 - 2251401 | 5 | 257 | GSVIVG01000284001 | 29.0/7.64 | 3 | 0 |
| Vv00g00710 | Chr Un | - | 2365748 - 2374087 | 6 | 320 | GSVIVG01000289001 | 36.1/8.76 | 4 | 0 |
| Vv00g00720 | Chr Un | - | 2379078 - 2380472 | 4 | 176 | GSVIVG01000290001 | 20.0/6.20 | 2 | 0 |
| Vv00g00750 | Chr Un | - | 2628682 - 2638367 | 5 | 247 | GSVIVG01000299001 | 28.1/8.92 | 2 | 0 |
| Vv00g00760 | Chr Un | - | 2643334 - 2645156 | 4 | 208 | GSVIVG01000300001 | 23.2/6.65 | 2 | 0 |
| Vv00g00800 | Chr Un | - | 2851713 - 2854530 | 6 | 320 | GSVIVG01000307001 | 36.3/9.25 | 4 | 0 |
| Vv00g25060 | Chr 13_random | - | 2645551 - 2649242 | 7 | 297 | GSVIVG01000828001 | 33.7/6.16 | 3 | 1 |
| Vv00g25070 | Chr 13_random | + | 2655022 - 2657830 | 6 | 314 | GSVIVG01000829001 | 36.1/6.16 | 3 | 0 |
| Vv01g05380 | 1 | - | 6088812 - 6096126 | 6 | 316 | GSVIVG01011598001 | 35.2/9.04 | 4 | 3 |
| Vv03g02080 | 3 | - | 17907034 - 7917706 | 9 | 520 | GSVIVG01015823001 | 58.6/9.31 | 4 | 0 |
| Vv06g10680 | 6 | + | 16907788 - 16910143 | 6 | 321 | GSVIVG01037250001 | 36.4/5.61 | 4 | 2 |
| Vv08g00710 | 8 | + | 3372322 - 3373625 | 4 | 301 | GSVIVG01022465001 | 33.7/8.20 | 2 | 1 |
| Vv18g03470 | 18 | - | 4338954 - 4341582 | 6 | 281 | GSVIVG01009021001 | 32.0/6.34 | 4 | 2 |
| ***C. papaya*** |  |  |  |  |  |  |  |  |  |
| Cp00213g00130 | supercontig_213 | + | 378790-381648 | 5 | 316 | evm.TU.supercontig_213.13 | 36.1/6.68 | 4 | 2 |
| Cp00002g01210 | supercontig_2 | + | 2025542-2027782 | 6 | 316 | evm.TU.supercontig_2.121 | 35.7/9.25 | 4 | 3 |
| Cp00003g03400 | supercontig_3 | + | 2356147-2358553 | 6 | 314 | evm.TU.supercontig_3.340 | 35.7/7.75 | 4 | 3 |
| Cp36671g00010 | contig_36671 | - | 662-2230 | 6 | 315 | evm.TU.contig_36671.1 | 36.3/5.74 | 4 | 2 |
| Cp00157g00670 | supercontig_157 | - | 436263-438507 | 6 | 315 | evm.TU.supercontig_157.67 | 35.5/6.05 | 4 | 2 |
| Cp00197g00020 | supercontig_197 | + | 23072-25490 | 6 | 318 | evm.TU.supercontig_197.2 | 35.8/5.80 | 4 | 2 |
| Cp00197g00010 | supercontig_197 | + | 13334-15207 | 6 | 293 | evm.TU.supercontig_197.1 | 33.5/6.32 | 2 | 2 |
| Cp00036g01250 | supercontig_36 | - | 996165-999052 | 7 | 290 | evm.TU.supercontig_36.125 | 33.2/5.81 | 4 | 2 |
| Cp00161g00040 | supercontig_161 | + | 37817 - 39348 | 4 | 325 | evm.TU.supercontig_161.4 | 36.8/8.83 | 4 | 1 |
| Cp04842g00010 | supercontig_4842 | - | 1348-2891 | 6 | 236 | evm.TU.supercontig_4842.1 | 26.9/5.09 | 2 | 2 |
| Cp00042g00810 | supercontig_42 | - | 728558-729526 | 3 | 255 | evm.TU.supercontig_42.81 | 28.5/9.92 | 3 | 1 |
| Cp00042g00660 | supercontig_42 | - | 603606-605037 | 2 | 137 | evm.TU.supercontig_42.66 | 15.5/5.56 | 1 | 1 |
| ***G. max*** |  |  |  |  |  |  |  |  |  |
| Gm04g27100 | 4 | + | 31032214 - 31034316 | 6 | 291 | 16256359 | 33.1/7.73 | 4 | 2 |
| Gm05g31250 | 5 | + | 36389317 - 36392416 | 5 | 315 | 16260268 | 35.6/5.89 | 4 | 2 |
| Gm07g12030 | 7 | + | 10292347 - 10296079 | 6 | 317 | 16267026 | 35.5/8.87 | 4 | 3 |
| Gm07g28080 | 7 | - | 32034631-32045326 | 6 | 316 | 16268109 | 35.7/9.41 | 4 | 3 |
| Gm08g06100 | 8 | - | 4327773 - 4329920 | 5 | 315 | 16270149 | 35.4/9.28 | 4 | 2 |
| Gm08g14460 | 8 | + | 10496668 - 10499242 | 5 | 315 | 16271133 | 35.6/6.06 | 4 | 2 |
| Gm09g30190 | 9 | - | 37071236 - 37075086 | 6 | 317 | 16276694 | 35.5/8.85 | 4 | 3 |
| Gm11g21460 | 11 | - | 18477409-18480015 | 9 | 330 | 16284567 | 37.5/8.96 | 2 | 1 |
| Gm11g21480 | 11 | - | 18484799 - 18491438 | 7 | 346 | 16284569 | 39.7/7.74 | 4 | 2 |
| Gm13g01870 | 13 | + | 1567448 - 1570329 | 5 | 316 | 16289380 | 35.9/6.48 | 4 | 2 |
| Gm13g26040 | 13 | - | 29263589-29265008 | 5 | 346 | 16291599 | 38.9/8.81 | 2 | 0 |
| Gm13g26960 | 13 | + | 30149473 - 30153870 | 6 | 314 | 16291697 | 35.7/7.75 | 4 | 2 |
| Gm13g26990 | 13 | + | 30172677 - 30174813 | 6 | 313 | 16291702 | 35.7/6.79 | 4 | 3 |
| Gm13g27000 | 13 | + | 30178813-30183375 | 7 | 295 | 16291703 | 34.1/9.41 | 2 | 2 |
| Gm13g27010 | 13 | - | 30184910 - 30187866 | 6 | 314 | 16291704 | 36.1/8.18 | 3 | 0 |
| Gm13g27020 | 13 | + | 30194052-30198241 | 6 | 320 | 16291705 | 36.1/5.50 | 4 | 2 |
| Gm15g14350 | 15 | - | 10814697-10817846 | 6 | 313 | 16298758 | 35.7/6.35 | 4 | 1 |
| Gm15g38010 | 15 | + | 44068082 - 44073057 | 6 | 314 | 16300371 | 35.8/8.55 | 4 | 2 |
| Gm15g38040 | 15 | + | 44113328 - 44115512 | 6 | 313 | 16300376 | 35.7/7.11 | 4 | 3 |
| Gm15g38060 | 15 | - | 44141240 - 44144187 | 6 | 314 | 16300378 | 36.1/8.51 | 3 | 0 |
| Gm15g38070 | 15 | + | 44158432 - 44162532 | 6 | 320 | 16300381 | 36.0/5.78 | 4 | 2 |
| Gm20g01460 | 20 | + | 992584-997275 | 6 | 313 | 16315156 | 35.5/9.36 | 4 | 3 |
| ***C. sativus*** |  |  |  |  |  |  |  |  |  |
| Cs138380 | scaffold01063 | - | 31177 - 33678 | 6 | 321 |  | 36.3/5.98 | 4 | 1 |
| Cs217870 | scaffold01658 | + | 96448 - 98870 | 5 | 314 |  | 35.6/6.12 | 4 | 2 |
| Cs234800 | scaffold02004 | + | 10347 - 14878 | 6 | 316 |  | 35.6/9.21 | 4 | 3 |
| Cs234810 | scaffold02004 | + | 23370 - 25184 | 5 | 293 |  | 33.5/8.89 | 4 | 3 |
| Cs273000 | scaffold02511 | - | 6093 - 8719 | 5 | 321 |  | 36.5/6.73 | 4 | 2 |
| Cs307970 | scaffold02978 | + | 256856 - 259078 | 7 | 397 |  | 45.6/5.18 | 1 | 0 |
| Cs307980 | scaffold02978 | - | 260327 - 262347 | 5 | 246 |  | 28.4/9.36 | 1 | 0 |
| Cs308080 | scaffold02978 | + | 316729 - 319436 | 6 | 318 |  | 36.3/6.49 | 4 | 1 |
| Cs308090 | scaffold02978 | + | 321938 - 324789 | 6 | 315 |  | 36.0/6.43 | 4 | 1 |
| Cs308100 | scaffold02978 | - | 325202 - 328147 | 6 | 318 |  | 36.5/8.53 | 2 | 0 |
| Cs340270 | scaffold03356 | - | 2757084 - 2758487 | 4 | 318 |  | 36.6/8.70 | 2 | 0 |
| ***O. sativa*** |  |  |  |  |  |  |  |  |  |
| Os01g31270 | 1 | - | 17110230 - 17105524 | 6 | 289 | Os01g31270 | 31.9/5.74 | 3 | 2 |
| Os02g51750 | 2 | - | 31700503 - 31698161 | 5 | 314 | Os02g51750 | 35.5/6.78 | 4 | 2 |
| Os05g31750 | 5 | - | 18496597 - 18494331 | 5 | 319 | Os05g31750 | 35.3/6.47 | 3 | 0 |
| Os05g31760 | 5 | - | 18500678 - 18503121 | 4 | 372 | Os05g31760 | 40.2/5.62 | 3 | 2 |
| Os06g11800 | 6 | + | 6250717 - 6253708 | 5 | 317 | Os06g11800 | 35.7/6.21 | 4 | 2 |
| Os07g46550 | 7 | - | 27802411 - 27800556 | 6 | 304 | Os07g46550 | 35.0/9.31 | 3 | 1 |
| Os08g32970 | 8 | + | 20459698 - 20461621 | 3 | 321 | Os08g32970 | 35.4/9.44 | 4 | 3 |
| Os09g20330 | 9 | + | 12196141 - 12197683 | 4 | 363 | Os09g20330 | 40.1/10.86 | 1 | 0 |
| Os09g23160 | 9 | + | 13706488 - 13711511 | 6 | 315 | Os09g23160 | 35.6/8.89 | 4 | 3 |
| Os09g27990 | 9 | - | 17001374 - 16999259 | 5 | 319 | Os09g27990 | 36.5/8.65 | 3 | 0 |
| ***S. bicolor*** |  |  |  |  |  |  |  |  |  |
| Sb01g035040 | 1 | - | 58570198 - 58571233 | 4 | 246 | 5049403 | 27.7/5.86 | 3 | 1 |
| Sb01g035050 | 1 | + | 58572276 - 58573720 | 4 | 370 | 5029924 | 41.0/8.91 | 3 | 0 |
| Sb02g024090 | 2 | + | 58105800 - 58109583 | 6 | 316 | 5051146 | 35.8/8.89 | 4 | 3 |
| Sb02g026390 | 2 | - | 61478981 - 61480372 | 5 | 336 | 5033923 | 37.5/8.48 | 3 | 0 |
| Sb02g041850 | 2 | - | 75608036 - 75610408 | 6 | 333 | 5052186 | 36.9/7.01 | 3 | 1 |
| Sb03g004990 | 3 | + | 5221375 - 5223060 | 5 | 322 | 5035194 | 35.1/6.09 | 3 | 2 |
| Sb04g027590 | 4 | + | 57499865 - 57502059 | 5 | 314 | 5055533 | 35.2/6.78 | 4 | 2 |
| Sb07g020760 | 7 | + | 53640962 - 53641924 | 1 | 320 | 5058738 | 35.5/9.38 | 4 | 3 |
| Sb09g018980 | 9 | + | 47378859 - 47380686 | 5 | 361 | 5060424 | 39.8/5.99 | 3 | 2 |
| Sb10g007760 | 10 | + | 7611098 - 7614387 | 5 | 314 | 5061529 | 35.4/7.16 | 4 | 2 |
| ***Z. mays*** |  |  |  |  |  |  |  |  |  |
| Zm01g15800 | 1 | - | 61621307-61622780 | 4 | 365 | AC204530.4_FGP003 | 40.5/8.25 | 3 | 0 |
| Zm02g30240 | 2 | - | 178875271 - 178879399 | 6 | 316 | GRMZM2G009136_P01 | 35.8/8.75 | 4 | 3 |
| Zm03g04200 | 3 | + | 14225524 - 14227097 | 5 | 320 | GRMZM2G031040_P01 | 34.6/6.11 | 3 | 2 |
| Zm08g13570 | 8 | + | 91421518 - 91423109 | 5 | 394 | GRMZM2G034229_P01 | 43.1/8.80 | 2 | 1 |
| Zm08g03950 | 8 | - | 15981355 - 15982854 | 5 | 328 | GRMZM2G048763_P01 | 35.9/6.09 | 3 | 2 |
| Zm05g40790 | 5 | + | 208194968-208197531 | 5 | 314 | GRMZM2G061950_P01 | 35.3/7.16 | 4 | 2 |
| Zm06g16450 | 6 | + | 112137446 -112140696 | 4 | 285 | GRMZM2G064993_P01 | 32.2/8.63 | 2 | 1 |
| Zm07g13390 | 7 | + | 106994728 - 106998257 | 7 | 316 | GRMZM2G067752_P01 | 35.8/8.89 | 4 | 3 |
| Zm06g23280 | 6 | + | 142656108 - 142657674 | 6 | 391 | GRMZM2G132442_P01 | 42.9/6.19 | 2 | 2 |
| Zm06g23270 | 6 | - | 142647642 - 142648278 | 2 | 178 | GRMZM2G132461_P01 | 19.9/6.93 | 3 | 0 |
| Zm02g31380 | 2 | - | 185377488 - 185378849 | 5 | 324 | GRMZM2G134502_P01 | 35.8/9.12 | 3 | 0 |
| Zm04g13650 | 4 | + | 79758223 - 79759176 | 1 | 317 | GRMZM2G172834_P01 | 35.9/9.48 | 4 | 3 |
| ***B. distachyon*** |  |  |  |  |  |  |  |  |  |
| Bd1g18990 | 1 | + | 15199470-15201246 | 3 | 306 | BRADI1G18990.1 | 33.1/4.98 | 3 | 1 |
| Bd1g45487 | 1 | - | 43716844-43719610 | 5 | 315 | BRADI1G45487.1 | 35.2/6.53 | 4 | 2 |
| Bd1g62120 | 1 | - | 61429438-61430589 | 4 | 196 | BRADI1G62120.1 | 21.7/5.49 | 2 | 1 |
| Bd1g62130 | 1 | + | 61435902-61437122 | 2 | 319 | BRADI1G62130.1 | 35.4/8.84 | 3 | 0 |
| Bd2g13620 | 2 | + | 12088168-12089489 | 4 | 289 | BRADI2G13620.1 | 31.7/4.94 | 4 | 2 |
| Bd2g26760 | 2 | - | 25498772-25500154 | 3 | 316 | BRADI2G26760.1 | 35.0/6.44 | 3 | 0 |
| Bd2g26770 | 2 | + | 25504383-25506587 | 6 | 369 | BRADI2G26770.1 | 40.9/5.39 | 3 | 2 |
| Bd3g36240 | 3 | + | 38514251-38515498 | 5 | 308 | BRADI3G36240.1 | 34.8/9.74 | 4 | 3 |
| Bd3g58830 | 3 | + | 58187798-58190196 | 5 | 315 | BRADI3G58830.1 | 35.4/6.12 | 4 | 2 |
| Bd4g29680 | 4 | + | 35210432-35215584 | 6 | 315 | BRADI4G29680.1 | 35.4/8.85 | 4 | 3 |
| Bd4g31920 | 4 | - | 37663951-37665383 | 5 | 314 | BRADI4G31920.1 | 36.3/8.93 | 2 | 1 |

^a^ represents the chromosomal location of a gene, ^b^ type of strand, ^c^ AA means number of amino acids, ^d^ molecular weight and isoelectric point calculated at the Expasy proteomics server (<http://ca.expasy.org/>). The genomic co-ordinates and ORF regions were obtained from their respective genome sequence browsers as described in materials and methods in the text.
