# Supplementary material for: Genome-wide Comparative Analysis of Annexin Superfamily in Plants
Source: PLoS One. 2012 Nov 2;7(11):e47801. doi: 10.1371/journal.pone.0047801 (PMC3487801; doi:10.1371/journal.pone.0047801)
Supplement: Table S2 — Gene structure organizations in Spike moss, moss, Arabidopsis and rice showing the total number of exons. (DOC) [file pone.0047801.s004.doc]

**Table S2 Gene structure organizations in Spike moss, moss, *Arabidopsis*** and rice showing the total number of exons.

|  | **Exon 1** | | | **Exon 2** | | | **Exon 3** | | | **Exon 4** | | | **Exon 5** | | | **Exon 6** | | | **Exon 7** | |  | |
| --- | --- | --- | --- | --- | --- | --- | --- | --- | --- | --- | --- | --- | --- | --- | --- | --- | --- | --- | --- | --- | --- | --- |
| **Gene ID** | length^a^ | AA^b^ | Intron phase^c^ | length^a^ | AA^b^ | Intron phase^c^ | length^a^ | AA^b^ | Intron phase^c^ | length^a^ | AA^b^ | Intron phase^c^ | length^a^ | AA^b^ | Intron phase^c^ | length^a^ | AA^b^ | Intron phase^c^ | length^a^ | AA^b^ | | No. of Introns^c^ |
| ***Spike moss*** |  |  |  |  |  |  |  |  |  |  |  |  |  |  |  |  |  |  |  |  | |  |
| estExt_fgenesh1_kg.C_740007 | 76 | K/G | 1 | 146 | E/Q | 0 | 219 | K/L | 0 | - | - | - | 216 | K/A | 0 | 90 | K/V | 0 | 201 | - | | 5 |
| estExt_Genewise1Plus.C_50869 | 76 | K/G | 1 | 146 | E/Q | 0 | 219 | K/L | 0 | - | - | - | 216 | K/A | 0 | 90 | K/V | 0 | 201 | - | | 5 |
| e_gw1.74.90.1 | 76 | K/G | 1 | 146 | E/Q | 0 | 219 | K/L | 0 | - | - | - | 216 | K/A | 0 | 90 | R/V | 0 | 201 | - | | 5 |
| e_gw1.16.385.1 | 76 | K/G | 1 | 146 | E/Q | 0 | 219 | K/L | 0 | - | - | - | 216 | K/A | 0 | 90 | K/V | 0 | 201 | - | | 5 |
| fgenesh1_kg.C_scaffold_5000033 | 76 | K/G | 1 | 146 | E/Q | 0 | 219 | K/L | 0 | - | - | - | 216 | K/A | 0 | 90 | Q/E | 0 | 201 | - | | 5 |
| ***Moss*** |  |  |  |  |  |  |  |  |  |  |  |  |  |  |  |  |  |  |  |  | |  |
| Pp1s37_276V6 | 73 | R/G | 1 | 146 | E/K | 0 | 219 | K/L | 0 | - | - | - | 216 | K/S | 0 | 90 | Q/E | 0 | 201 | - | | 5 |
| Pp1s219_3V6 | 73 | K/G | 1 | 146 | E/E | 0 | 219 | K/L | 0 | - | - | - | 216 | K/A | 0 | 90 | E/E | 0 | 363 | - | | 5 |
| Pp1s6_292V6 | 73 | K/G | 1 | 146 | E/K | 0 | 231 | K/L | 0 | - | - | - | 213 | E/S | 0 | 90 | E/E | 0 | 363 |  | | 5 |
| Pp1s1_594V6 | 73 | K/G | 1 | 146 | E/K | 0 | 231 | K/L | 0 | - | - | - | 219 | Q/S | 0 | 90 | E/E | 0 | 378 | - | | 5 |
| Pp1s102_141V6 | 73 | K/G | 1 | 146 | E/K | 0 | 228 | K/S | 0 | 75 | K/L | 0 | 213 | Q/S | 0 | 90 | E/E | 0 | 363 | - | | 6 |
| Pp1s38_63V6 | 76 | R/G | 1 | - | - | - | 377 | K/S | 0 | 75 | K/L | 0 | 213 | K/V | 0 | 90 | E/E | 0 | 357 | - | | 5 |
| Pp1s61_299V6 | 76 | S/G | 1 |  |  |  | 377 | K/R | 0 | 75 | K/L | 0 | 213 | K/A | 0 | 90 | E/D | 0 | 396 | I/K | | 6 |
| ***Arabidopsis*** |  |  |  |  |  |  |  |  |  |  |  |  |  |  |  |  |  |  |  |  | |  |
| At1g35720 (*AnnAt1*) | 76 | E/G | 1 | 146 | E/R | 0 | - | - | - | - | - | - | - | - | - | - | - | - | 741 | - | | 2 |
| At5g65020 (*AnnAt2*) | 76 | S/G | 1 | 146 | E/R | 0 | 219 | K/L | 0 | - | - | - | 213 | K/N | 0 |  |  |  | 300 | - | | 4 |
| At2g38760 (*AnnAt3*) | 76 | R/G | 1 | 146 | M/K | 0 | 234 | K/L | 0 | - | - | - | 213 | K/D | 0 | 93 | K/V | 0 | 204 | - | | 5 |
| At2g38750 (*AnnAt4*) | 49 | A/G | 1 | 182 | N/T | 0 | 219 | K/L | 0 | - | - | - | 222 | G/G | 0 | 72 | K/I | 0 | 216 | - | | 5 |
| At1g68090 (*AnnAt5*) | 76 | K/G | 1 | 146 | K/K | 0 | 219 | R/V | 0 | - | - | - | 216 | K/A | 0 | 90 | K/A | 0 | 204 | - | | 5 |
| At5g10220 (*AnnAt6*) | 76 | K/G | 1 | 146 | E/R | 0 | 219 | K/L | 0 | - | - | - | - | - | - | - | - | - | 516 | - | | 3 |
| At5g10230 (*AnnAt7*) | 76 | K/G | 1 | 146 | E/R | 0 | 219 | K/L | 0 | - | - | - | - | - | - | - | - | - | 510 | - | | 3 |
| At5g12380 (*AnnAt8*) | 73 | Q/G | 1 | 146 | E/R | 0 | 219 | R/L | 0 | - | - | - | 213 | K/D | 0 | 90 | K/V | 0 | 210 | - | | 5 |
| ***Rice*** |  |  |  |  |  |  |  |  |  |  |  |  |  |  |  |  |  |  |  |  | |  |
| Os01g31270 | 76 | Q/G | 1 | 146 | E/R | 0 | 138 | E/L | 0 | - | - | - | 213 | K/A | 0 | 93 | K/V | 0 | 204 | - | | 5 |
| Os02g51750 | 76 | K/G | 1 | 146 | E/R | 0 | 219 | K/L | 0 | - | - | - | 213 | K/D | 0 | - | - | - | 291 | - | | 4 |
| Os05g31750 | 40 | S/G | 1 | 188 | K/N | 0 | 219 | G/L | 0 | - | - | - | 231 | E/D | 0 | - | - | - | 282 | - | | 4 |
| Os05g31760 | 223 | Q/G | 1 | 146 | R/S | 0 | - | - | - | - | - | - | 546 | E/V | 0 | - | - | - | 204 | - | | 3 |
| Os06g11800 | 76 | Q/G | 1 | 146 | E/R | 0 | 225 | K/L | 0 | - | - | - | 213 | K/D | 0 | - | - | - | 294 | - | | 4 |
| Os07g46550 | 76 | Q/V | 1 | 206 | W/K | 0 | 114 | E/M | 0 | - | - | - | 225 | E/D | 0 | 90 | K/V | 0 | 204 | - | | 5 |
| Os08g32970 | - | - | - | - | - | - | - | - | - | - | - | - | 672 | K/A | 0 | 90 | K/V | 0 | 204 | - | | 2 |
| Os09g20330 | 70 | S/G | 1 | 215 | I/R | 0 | 219 | E/V | 0 | - | - | - | - | - | - | - | - | - | 588 | - | | 3 |
| Os09g23160 | 76 | K/G | 1 | 146 | K/K | 0 | 219 | K/L | 0 | - | - | - | 216 | K/V | 0 | 90 | K/V | 0 | 201 | - | | 5 |
| Os09g27990 | - | - | - | 210 | E/L | 0 | 227 | R/L | 0 | - | - | - | 220 | K/A | 0 | 90 | K/L | 0 | 213 | - | | 4 |

^a^ represents the length of each exon in base pairs (bp), ^b^ AA type of amino acid between the two exons, ^c^ numbers at the exon-intron junctions as intron phases, intron phase 1 (after the first base of a codon) or intron phase 0 (between codons).
